# Fundamental noisy multiparameter quantum bounds

**DOI:** 10.1038/s41598-018-37583-7

**Published:** 2019-01-31

**Authors:** Shibdas Roy

**Affiliations:** Department of Physics, University of Warwick, Coventry, CV4 7AL United Kingdom

## Abstract

Quantum multiparameter estimation involves estimating multiple parameters simultaneously and can be more precise than estimating them individually. Our interest here is to determine fundamental quantum limits to the achievable multiparameter estimation precision in the presence of noise. We first present a lower bound to the estimation error covariance for a noisy initial probe state evolving through a noiseless quantum channel. We then present a lower bound to the estimation error covariance in the most general form for a noisy initial probe state evolving through a noisy quantum channel. We show conditions and accordingly measurements to attain these estimation precision limits for noisy systems. We see that the Heisenberg precision scaling of 1/*N* can be achieved with a probe comprising *N* particles even in the presence of noise. In fact, some noise in the initial probe state or the quantum channel can serve as a feature rather than a bug, since the estimation precision scaling achievable in the presence of noise in the initial state or the channel in some situations is impossible in the absence of noise in the initial state or the channel. However, a lot of noise harms the quantum advantage achievable with *N* parallel resources, and allows for a best precision scaling of ${\bf{1}}/\sqrt{{\boldsymbol{N}}}$. Moreover, the Heisenberg precision limit can be beaten with noise in the channel, and we present a super-Heisenberg precision limit with scaling of 1/*N*^2^ for optimal amount of noise in the channel, characterized by one-particle evolution operators. Furthermore, using *γ*-particle evolution operators for the noisy channel, where *γ* > 1, the best precision scaling attainable is 1/*N*^2*γ*^, which is otherwise known to be only possible using 2*γ*-particle evolution operators for a noiseless channel.

## Introduction

Studying quantum multiparameter estimation has recently been of significant interest^1–14^. While quantum resources allow for surpassing measurement limits set by classical physics^15–17^, it is important to consider fundamental measurement limits set by quantum mechanics. Although quantum estimation of a single parameter captures many scenarios^18^, the practically more relevant problem of estimating multiple parameters simultaneously has started drawing more attention, mainly because unlike in quantum single-parameter estimation case, quantum measurements required to attain multiparameter bounds do not necessarily commute^5,19–21^.

Multiparameter estimation using a pure (i.e. noiseless) probe state under unitary (i.e. noiseless) evolution has been studied, e.g. in ref.^5^. This work, like most in the literature, used symmetric logarithmic derivatives (SLDs), as used by Helstrom^19^, to define the quantum Fisher information matrix (QFIM)^22^. Then, the estimation error covariance (that is the multiparameter counterpart to the mean-squared estimation error in single parameter estimation) is lower-bounded by the inverse of the QFIM and the bound is called a quantum Cramér-Rao bound (QCRB)^23^. Such a QFIM for a probe with multiple particles under unitary evolution via one particle Hamiltonians^5,23,24^ was shown to depend only on the one- and two-particle reduced density operators^24^ of the probe state. However, when the initial probe state is mixed (i.e. noisy) but the quantum channel is unitary, even for single parameter estimation, only an upper bound to such an SLD-based QFIM (and therefore, a lower bound to the corresponding QCRB) can be explicitly established in general^25,26^. Although noiseless quantum parameter estimation has been studied extensively and is well understood, it is important to study and better understand fundamental quantum estimation limits in more practical noisy situations^2,22,26–50^.

In this article, we present a multiparameter QCRB for a noisy initial state evolving unitarily, based on anti-symmetric logarithmic derivatives (ALDs)^51,52^, that lend a convenient way to study noisy quantum metrology. Moreover, we use a similar ALD-approach to present an upper bound to the QFIM (like in refs ^22,26^) for the case of impure initial states under arbitrary evolution. That is, we consider a noisy quantum channel and a mixed intial probe state and define a quantum lower bound for the estimation error covariance in this general-most case. Such bounds for an *N*-particle probe state depend on the one- and two-particle reduced density operators only, similar to the case of pure state evolving unitarily in ref. ^5^. We also provide conditions and accordingly measurements that allow to attain these bounds.

Our results here are fundamentally profound because of several reasons. Firstly, the tight bounds presented here are explicitly computable (e.g. in terms of the Kraus operators of a noisy channel), without any knowledge of the eigenvalues and the eigenvectors of the evolved probe state^20,27^ and are not known to be possible for these most general noisy cases using the conventional SLD-approach. A similar bound with SLDs was obtained for single parameter estimation earlier^26,31,53^, but it was not considered tight being an upper bound to the SLD QFI, and accordingly a tighter bound, linear in the number *N* of resources was considered. Secondly, our bounds are such that the quantum enhancement to the estimation precision is provided by the two-particle reduced density matrices of the probe state and the attainability of the quantum enhancement is determined solely by the one-particle reduced density matrices of the probe state, when the channel is characterized by one-particle evolution operators, even in the presence of noise, similar to the noiseless case from ref. ^5^. Thirdly, the results here suggest that the Heisenberg scaling of 1/*N* in the estimation precision, with *N* number of resources, is achievable even in the presence of noise. Moreover, some noise in the quantum channel or the initial probe state can act as a feature rather than a bug, since we see that there are situations when it is not possible to attain the Heisenberg limit in the absence of noise in the channel or the initial state, but it is possible in the presence of noise in the channel or the initial state. However, too much noise in the initial state or the channel harms the quantum advantage achievable with *N* parallel resources.

Furthermore, we show that the Heisenberg precision limit can be beaten with noise in the quantum channel. The best achievable precision limit for non-unitary channel is then determined by two-particle reduced density operators of the evolved probe state being maximally entangled and one-particle reduced density operators being maximally mixed, and corresponds to a precision scaling of 1/*N*^2^, attained with one-particle evolution operators for the channel. Further, using *γ*-particle (instead of one-particle) evolution operators for a noisy channel, where *γ* > 1, the best precision scaling achievable is 1/*N*^2*γ*^, that is otherwise known as achievable with 2*γ*-particle evolution operators of a noiseless channel.

Before we proceed, it is important to explicitly point out why the non-standard ALD-approach instead of the standard SLD-approach is adopted in this paper. The way we choose the ALDs in this article, it turns out that the ALD-based QFIM is an upper bound to the standard SLD-based QFIM for the multiparameter noiseless channel case. As already pointed out, such an upper bound to the SLD QFIM for single parameter estimation has been obtained earlier, but it was not considered a tight bound, since beating the SLD QFIM would mean that the Heisenberg limit would be beaten. However, we show here that such an upper bound to the SLD QFIM can be tight too, but the use of ALD-approach indicates that the Heisenberg limit is not beaten for the noiseless channel case. Thus, the QFIM obtained here for the noiseless channel case cannot be obtained using the SLD-approach and the corresponding equivalent bound obtained using SLDs would seem to beat the Heisenberg limit. Moreover, for the multiparameter noisy channel case, the upper bound to the ALD QFIM here cannot be obtained using SLDs, since it would be an upper bound to the aforementioned upper bound to the SLD QFIM, which, therefore, for single parameter estimation^26^ was considered even looser. We show that such an upper bound to the ALD QFIM can also be tight, implying that the Heisenberg limit can be beaten. It is unlikely that there exists some other logarithmic derivative for which the QFIM would be the upper bound to the ALD QFIM, suggesting that the Heisenberg limit is still not beaten.

## Multiparameter Quantum Cramér-Rao Bound

An experiment for estimation of some unknown parameters corresponding to a quantum process involves three stages. First, a probe state is prepared in an initial state, comprising *N* number of resources, and evolves under the action of the quantum process. The second stage involves choosing a suitable measurement, applied to the evolved probe state. The final step involves associating, through an estimator, each experimental result with an estimation of the parameters^26^. The Heisenberg limit to the estimation precision is then the precision scaling of 1/*N*.

Consider that a probe state $\hat{\rho }$ acquires *q* number of parameters ${\boldsymbol{\theta }}={[{\theta }_{1}{\theta }_{2}\ldots {\theta }_{q}]}^{T}$ via a unitary transformation $\hat{U}({\boldsymbol{\theta }})$, and we seek the best quantum strategy to estimate the parameters from the evolved probe state, $\hat{\rho }({\boldsymbol{\theta }})=\hat{U}({\boldsymbol{\theta }})\hat{\rho }{\hat{U}}^{\dagger }({\boldsymbol{\theta }})$. Let a measurement performed on the evolved state $\hat{\rho }({\boldsymbol{\theta }})$ be given by some positive operator valued measure (POVM) $\{{\hat{P}}_{m}\}$. The conditional probability to obtain the outcome *m* given the parameters have the value ***θ*** is $p(m|{\boldsymbol{\theta }})={\rm{Tr}}\,({\hat{P}}_{m}\hat{\rho }({\boldsymbol{\theta }}))$. The estimates $\mathop{{\boldsymbol{\theta }}}\limits^{ \sim }(m)={[{\mathop{\theta }\limits^{ \sim }}_{1}(m){\mathop{\theta }\limits^{ \sim }}_{2}(m)\ldots {\mathop{\theta }\limits^{ \sim }}_{q}(m)]}^{T}$ are unbiased if

1 $$\sum _{m}\,p(m|{\boldsymbol{\theta }}){\mathop{\theta }\limits^{ \sim }}_{j}(m)={\theta }_{j}\,{\rm{\forall }}j.$$

Then, the estimation error covariance is

2 $$V\,[\mathop{{\boldsymbol{\theta }}}\limits^{ \sim }(m)]=\sum _{m}\,p(m|{\boldsymbol{\theta }})\,(\mathop{{\boldsymbol{\theta }}}\limits^{ \sim }(m)-{\boldsymbol{\theta }})\,{(\mathop{{\boldsymbol{\theta }}}\limits^{ \sim }(m)-{\boldsymbol{\theta }})}^{T}.$$

Then, for unbiased estimators, the above covariance satisfies the Cramér-Rao inequality:

3 $$\nu V\,[\mathop{{\boldsymbol{\theta }}}\limits^{ \sim }(m)]\ge {[{J}_{C}({\boldsymbol{\theta }})]}^{-1},$$

where *ν* is the number of times the overall experiment is repeated and *J*_*C*_(***θ***) is the classical Fisher Information Matrix (FIM), given by

4 $${J}_{C}^{jk}=\sum _{m}\,\frac{1}{p(m|{\boldsymbol{\theta }})}\frac{\partial }{\partial {\theta }_{j}}p(m|{\boldsymbol{\theta }})\frac{\partial }{\partial {\theta }_{k}}p(m|{\boldsymbol{\theta }}).$$

Further, the maximisation of the FIM over all possible POVMs yields the quantum Fisher Information Matrix (QFIM), *J*_*Q*_(***θ***), which is determined from^51,52^:

5 $$\frac{1}{2}\,({\hat{L}}_{k}\hat{\rho }({\boldsymbol{\theta }})+\hat{\rho }({\boldsymbol{\theta }}){\hat{L}}_{k}^{\dagger })=\frac{{\rm{\partial }}}{{\rm{\partial }}{\theta }_{k}}\hat{\rho }({\boldsymbol{\theta }}).$$

where ${\hat{L}}_{k}$ is an operator. The QFIM *J*_*Q*_(***θ***) is then^51^:

6 $${J}_{Q}^{jk}=\frac{1}{2}{\rm{T}}{\rm{r}}\,[({\hat{L}}_{j}^{\dagger }{\hat{L}}_{k}+{\hat{L}}_{k}^{\dagger }{\hat{L}}_{j})\,\hat{\rho }({\boldsymbol{\theta }})],$$

Then, we have

7 $$\nu V\,[\mathop{{\boldsymbol{\theta }}}\limits^{ \sim }(m)]\ge {[{J}_{C}({\boldsymbol{\theta }})]}^{-1}\ge {[{J}_{Q}({\boldsymbol{\theta }})]}^{-1},$$

where ${\hat{L}}_{k}$ was taken to be Hermitian by Helstrom^19^, in which case it is called the symmetric logarithmic derivative (SLD). In general, ${\hat{L}}_{k}$ need not be Hermitian. We assume that ${\hat{L}}_{k}$ is anti-Hermitian, such that ${\hat{L}}_{k}^{\dagger }=-\,{\hat{L}}_{k}$^51,52^, in which case it is called the anti-symmetric logarithmic derivative (ALD). Thus, (6) defines a certain family of logarithmic derivatives, satisfying ${\rm{Tr}}\,[\hat{\rho }({\boldsymbol{\theta }}){\hat{L}}_{k}]=0$, such that a Hermitian ${\hat{L}}_{k}$ is an SLD and an anti-Hermitian ${\hat{L}}_{k}$ is an ALD^52^. Although ref. ^51^ considered a different (Bayesian waveform-) estimation problem, (7) can be similarly proven here. See Methods section.

Although the classical Cramér-Rao bound (i.e. the first inequality in (7)) can always be saturated, e.g. by a maximum likelihood estimator^54^, the QCRB (i.e. the second inequality in (7)) for SLDs are not known to be attainable in general. We claim that an ALD-based QCRB of the form (7) can be saturated (i.e. attained), when the QFIM is not rank deficient and the expectation of the commutator of every pair of the ALDs vanishes, similar to the case of SLD-based QCRB^5,55,56^:

8 $${\rm{T}}{\rm{r}}\,[({\hat{L}}_{j}^{\dagger }{\hat{L}}_{k}-{\hat{L}}_{k}^{\dagger }{\hat{L}}_{j})\,\hat{\rho }({\boldsymbol{\theta }})]={\rm{T}}{\rm{r}}\,([{\hat{L}}_{j},{\hat{L}}_{k}]\,\hat{\rho }({\boldsymbol{\theta }}))=0.$$

See Methods section. The above condition is trivially true for single parameter estimation. Then, the set of POVMs of cardinality *q* + 2, comprising the following *q* + 1 elements,

9 $$\begin{array}{ccc}{\hat{P}}_{0} & = & \hat{\rho }({\boldsymbol{\theta }})=\hat{U}({\boldsymbol{\theta }})\hat{\rho }{\hat{U}}^{\dagger }({\boldsymbol{\theta }}),\\ {\hat{P}}_{m} & = & \frac{{\rm{\partial }}\hat{\rho }({\boldsymbol{\theta }})}{{\rm{\partial }}{\theta }_{m}}=\frac{{\rm{\partial }}\hat{U}({\boldsymbol{\theta }})}{{\rm{\partial }}{\theta }_{m}}\hat{\rho }{\hat{U}}^{\dagger }({\boldsymbol{\theta }})+\hat{U}({\boldsymbol{\theta }})\hat{\rho }\frac{{\rm{\partial }}{\hat{U}}^{\dagger }({\boldsymbol{\theta }})}{{\rm{\partial }}{\theta }_{m}}\,{\rm{\forall }}m=1,\ldots ,q,\end{array}$$

along with one normalising element, saturates the QCRB (see Methods section). For pure states |*ψ*〉, the *q* + 1 projectors

10 $$\begin{array}{ccc}{\hat{P}}_{0} & = & \hat{\rho }({\boldsymbol{\theta }})=\hat{U}({\boldsymbol{\theta }})|\psi \rangle \langle \psi |{\hat{U}}^{\dagger }({\boldsymbol{\theta }}),\\ {\hat{P}}_{m} & = & \frac{{\rm{\partial }}\hat{U}({\boldsymbol{\theta }})}{{\rm{\partial }}{\theta }_{m}}|\psi \rangle \langle \psi |\frac{{\rm{\partial }}{\hat{U}}^{\dagger }({\boldsymbol{\theta }})}{{\rm{\partial }}{\theta }_{m}}\,{\rm{\forall }}m=1,\ldots ,q,\end{array}$$

along with one normalising element, saturates the QCRB. This follows from refs ^4,5^ (see Methods section).

## The QFIM for One-Particle Hamiltonians

Let us now consider that the unitary evolution is $\hat{U}({\boldsymbol{\theta }})={e}^{-i\hat{H}({\boldsymbol{\theta }})}$ and that the probe state $\hat{\rho }$ comprises *N* particles evolving under the one-particle Hamiltonian ${\hat{h}}^{[n]}={\sum }_{k=1}^{q}\,{\theta }_{k}{\hat{h}}_{k}^{[n]}$ for *n* = 1, …, *N*, such that^5^:

11 $$\hat{H}({\boldsymbol{\theta }})=\sum _{n=1}^{N}\,{\hat{h}}^{[n]}=\sum _{k=1}^{q}\,{\theta }_{k}\,\sum _{n=1}^{N}\,{\hat{h}}_{k}^{[n]}\equiv \sum _{k=1}^{q}\,{\theta }_{k}{\hat{H}}_{k}.$$

The generators ${\hat{H}}_{k}$ are assumed to not depend on ***θ*** and do not generally commute with each other. Then, as employed by ref. ^5^, we have^57^:

12 $$\frac{\partial \hat{U}({\boldsymbol{\theta }})}{\partial {\theta }_{k}}=-\,i\,{\int }_{0}^{1}\,d\alpha {e}^{-i(1-\alpha )\hat{H}(\theta )}\frac{\partial \hat{H}({\boldsymbol{\theta }})}{\partial {\theta }_{k}}{e}^{-i\alpha \hat{H}(\theta )}.$$

Then, we have

13 $$\frac{{\rm{\partial }}}{{\rm{\partial }}{\theta }_{k}}\hat{\rho }({\boldsymbol{\theta }})=\frac{{\rm{\partial }}}{{\rm{\partial }}{\theta }_{k}}(\hat{U}({\boldsymbol{\theta }})\hat{\rho }{\hat{U}}^{\dagger }({\boldsymbol{\theta }}))=-\,i\,[{\hat{M}}_{k}({\boldsymbol{\theta }}),\hat{\rho }({\boldsymbol{\theta }})],$$

where

14 $${\hat{M}}_{k}({\boldsymbol{\theta }})=i\frac{\partial \hat{U}({\boldsymbol{\theta }})}{\partial {\theta }_{k}}{\hat{U}}^{\dagger }({\boldsymbol{\theta }})=\hat{U}({\boldsymbol{\theta }}){\hat{A}}_{k}({\boldsymbol{\theta }}){\hat{U}}^{\dagger }({\boldsymbol{\theta }}),$$

with ${\hat{A}}_{k}({\boldsymbol{\theta }})={\int }_{0}^{1}\,d\alpha {e}^{i\alpha \hat{H}({\boldsymbol{\theta }})}{\hat{H}}_{k}{e}^{-i\alpha \hat{H}({\boldsymbol{\theta }})}$. We choose the operator ${\hat{L}}_{k}$ as the anti-Hermitian, ${\hat{L}}_{k}=-\,2i{\rm{\Delta }}{\hat{M}}_{k}$, where

15 $${\rm{\Delta }}{\hat{M}}_{k}\equiv {\hat{M}}_{k}({\boldsymbol{\theta }})-{\rm{T}}{\rm{r}}\,({\hat{M}}_{k}({\boldsymbol{\theta }})\hat{\rho }({\boldsymbol{\theta }})).$$

The QFIM from (6) then takes the form:

16 $${J}_{Q}^{jk}=2{\rm{T}}{\rm{r}}\,[({\rm{\Delta }}{\hat{A}}_{j}{\rm{\Delta }}{\hat{A}}_{k}+{\rm{\Delta }}{\hat{A}}_{k}{\rm{\Delta }}{\hat{A}}_{j})\,\hat{\rho }],$$

where

17 $$\begin{array}{ccc}{\rm{\Delta }}{\hat{A}}_{k} & = & {\hat{A}}_{k}({\boldsymbol{\theta }})-{\rm{T}}{\rm{r}}\,({\hat{A}}_{k}({\boldsymbol{\theta }})\hat{\rho })=\sum _{n}\,({\hat{b}}_{k}^{[n]}-{\rm{T}}{\rm{r}}\,({\hat{b}}_{k}^{[n]}{\hat{\rho }}^{[n]}))\equiv \sum _{n}\,{\hat{c}}_{k}^{[n]},\end{array}$$

with ${\hat{b}}_{k}^{[n]}={\int }_{0}^{1}\,d\alpha {e}^{i\alpha {\hat{h}}^{[n]}}{\hat{h}}_{k}^{[n]}{e}^{-i\alpha {\hat{h}}^{[n]}}$. Thus, (16) becomes:

$$\begin{array}{ccc}{J}_{Q}^{jk} & = & 2\,\sum _{n}\,{\rm{T}}{\rm{r}}\,[({\hat{c}}_{j}^{[n]}{\hat{c}}_{k}^{[n]}+{\hat{c}}_{k}^{[n]}{\hat{c}}_{j}^{[n]})\,{\hat{\rho }}^{[n]}]\,+\,2\,\sum _{n\ne m}\,{\rm{T}}{\rm{r}}\,[({\hat{c}}_{j}^{[n]}\otimes {\hat{c}}_{k}^{[m]}+{\hat{c}}_{k}^{[n]}\otimes {\hat{c}}_{j}^{[m]})\,{\hat{\rho }}^{[n,m]}]\\  & = & 4\,\sum _{n}\,{\rm{R}}{\rm{e}}\,[{\rm{T}}{\rm{r}}\,[{\hat{\rho }}^{[n]}{\hat{b}}_{j}^{[n]}{\hat{b}}_{k}^{[n]}]-{\rm{T}}{\rm{r}}\,[{\hat{\rho }}^{[n]}{\hat{b}}_{j}^{[n]}]\,{\rm{T}}{\rm{r}}\,[{\hat{\rho }}^{[n]}{\hat{b}}_{k}^{[n]}]]\,+\,4\,\sum _{n\ne m}\,{\rm{R}}{\rm{e}}\,[{\rm{T}}{\rm{r}}\,[{\hat{\rho }}^{[n,m]}\,({\hat{b}}_{j}^{[n]}\otimes {\hat{b}}_{k}^{[m]})]\,-\,{\rm{T}}{\rm{r}}\,[{\hat{\rho }}^{[n]}{\hat{b}}_{j}^{[n]}]\,{\rm{T}}{\rm{r}}\,[{\hat{\rho }}^{[m]}{\hat{b}}_{k}^{[m]}]]\\  & = & \sum _{n}\,{J}_{Q}^{jk,[1]}\,({\hat{\rho }}^{[n]})+\sum _{n\ne m}\,{J}_{Q}^{jk,[2]}\,({\hat{\rho }}^{[n,m]}),\end{array}$$

where ${J}_{Q}^{jk,[1]}$ depends only on one-particle reduced density matrix on subsystem *n* and ${J}_{Q}^{jk,[2]}$ depends on two-particle reduced density matrix on subsystems *n*, *m*.

We now restrict to permutationally invariant states^5^, i.e. states that are invariant under any permutation of its constituents: $\hat{\rho }={\hat{O}}_{\pi }\hat{\rho }{\hat{O}}_{\pi }^{\dagger }$ for all possible *π*, where ${\hat{O}}_{\pi }$ is the unitary operator for the permutation *π*. Then,

18 $${J}_{Q}^{jk}=N{J}_{Q}^{jk,[1]}\,({\hat{\rho }}^{[1]})+N(N-1){J}_{Q}^{jk,[2]}\,({\hat{\rho }}^{[1]},{\hat{\rho }}^{[2]}),$$

where

$${J}_{Q}^{jk,[1]}=4{\rm{R}}{\rm{e}}\,[{\rm{T}}{\rm{r}}\,[{\hat{\rho }}^{[1]}{\hat{b}}_{j}{\hat{b}}_{k}]-{\rm{T}}{\rm{r}}\,[{\hat{\rho }}^{[1]}{\hat{b}}_{j}]\,{\rm{T}}{\rm{r}}\,[{\hat{\rho }}^{[1]}{\hat{b}}_{k}]]$$

only depends on the first order reduced density matrix,

$${J}_{Q}^{jk,[2]}=4{\rm{R}}{\rm{e}}\,[{\rm{T}}{\rm{r}}\,[{\hat{\rho }}^{[2]}\,({\hat{\rho }}_{j}\otimes {\hat{b}}_{k})]-{\rm{T}}{\rm{r}}\,[{\hat{\rho }}^{[1]}{\hat{b}}_{j}]\,{\rm{T}}{\rm{r}}\,[{\hat{\rho }}^{[1]}{\hat{b}}_{k}]]$$

also depends on the second order reduced density matrix.

Then, similar observations can be made as were made in ref.^5^ for pure state. For example, if the probe state is a product state, i.e. $\hat{\rho }={\otimes }_{n=1}^{N}\,{\hat{\rho }}^{[n]}$, and permutationally invariant, then ${\hat{\rho }}^{[2]}={\hat{\rho }}^{[1]}\otimes {\hat{\rho }}^{[1]}$, such that ${J}_{Q}^{jk,[2]}=0$, and so ${J}_{Q}^{jk}=N{J}_{Q}^{jk,[1]}$. This implies that quantum correlations are necessary for achieving the Heisenberg scaling 1/*N*, which is evidently attainable even when the initial probe state is mixed. However, if both ${\hat{\rho }}^{[1]}$ and ${\hat{\rho }}^{[2]}$ are maximally mixed, the Heisenberg scaling is lost, i.e. too much quantum correlations harm the quantum advantage with *N* parallel resources^5,58^. Thus, any quantum enhancement to the estimation precision is provided by the two-particle reduced density matrices of the probe state.

Moreover, from (9), the set of POVMs, comprising

19 $$\begin{array}{ccc}{\hat{P}}_{0} & = & \hat{\rho }({\boldsymbol{\theta }})=\hat{U}({\boldsymbol{\theta }})\hat{\rho }{\hat{U}}^{\dagger }({\boldsymbol{\theta }}),\\ {\hat{P}}_{m} & = & \frac{{\rm{\partial }}\hat{\rho }({\boldsymbol{\theta }})}{{\rm{\partial }}{\theta }_{m}}=-\,i\,[{\hat{M}}_{m}({\boldsymbol{\theta }}),\hat{\rho }({\boldsymbol{\theta }})]\,{\rm{\forall }}m=1,\ldots ,q,\end{array}$$

along with one element accounting for normalisation, saturates the QCRB for (16), provided we have (8), i.e. here

20 $$\begin{array}{cccc} & 2{\rm{T}}{\rm{r}}\,[({\rm{\Delta }}{\hat{A}}_{j}{\rm{\Delta }}{\hat{A}}_{k}-{\rm{\Delta }}{\hat{A}}_{k}{\rm{\Delta }}{\hat{A}}_{j})\hat{\rho }] & = & 0\,{\rm{\forall }}j,k\\ \Rightarrow  & 4\,\sum _{n}\,{\rm{I}}{\rm{m}}\,{\rm{T}}{\rm{r}}\,[{\hat{\rho }}^{[n]}{\hat{b}}_{j}^{[n]}{\hat{b}}_{k}^{[n]}]\,+\,4\,\sum _{n\ne m}\,{\rm{I}}{\rm{m}}\,{\rm{T}}{\rm{r}}\,[{\hat{\rho }}^{[n,m]}\,({\hat{b}}_{j}^{[n]}\otimes {\hat{b}}_{k}^{[m]})] & = & 0\\ \Rightarrow  & 4\,\sum _{n}\,{\rm{I}}{\rm{m}}\,{\rm{T}}{\rm{r}}\,[{\hat{\rho }}^{[n]}{\hat{b}}_{j}^{[n]}{\hat{b}}_{k}^{[n]}] & = & 0,\end{array}$$

since ${\rm{Tr}}\,[{\hat{\rho }}^{[n,m]}\,({\hat{b}}_{j}^{[n]}\otimes {\hat{b}}_{k}^{[m]})]\in {\mathbb{R}}$. Hence, the attainability of the quantum enhancement to the estimation precision is determined solely by the one-particle reduced density matrices of the probe state.

## Estimating a Magnetic Field in Three Dimensions

Now consider the task of estimating the components of a magnetic field in three dimensions simultaneously using two-level systems. The Hamilton operator for this system is given by $$\hat{h}=\hat{{\boldsymbol{\mu }}}\cdot {\bf{B}}={\sum }_{k=1}^{3}\,{\hat{\mu }}_{k}{B}_{k}$$ = $${\sum }_{k=1}^{3}\,(\mu /2)$$$${B}_{k}{\hat{\sigma }}_{k}:\,={\sum }_{k=1}^{3}\,{\theta }_{k}{\hat{\sigma }}_{k}$$, where the magnetic moment $${\hat{\mu }}_{k}=\mu {\hat{\sigma }}_{k}/2$$ is proportional to the spin, $$\{{\hat{\sigma }}_{k}\}$$ are the unnormalized Pauli operators, and *θ*_*k*_ = *μB*_*k*_/2^5^.

Start with a Greenberger-Horne-Zeilinger (GHZ) type pure state $|{{\rm{\Phi }}}_{k}\rangle ={(|{\varphi }_{k}^{+}\rangle }^{\otimes N}+|{\varphi }_{k}^{-}{\rangle }^{\otimes N})/\sqrt{2}$, where $|{\varphi }_{k}^{\pm }\rangle$ is the eigenvector of the Pauli operator ${\hat{\sigma }}_{k}$ corresponding to the eigenvalue ±1 (*k* = 1, 2, 3 corresponding to the *X*, *Y*, and *Z* directions). These states are permutationally invariant with first and second order reduced density matrices ${\hat{\rho }}_{k}^{[1]}={1}_{2}/2$ and ${\hat{\rho }}_{k}^{[2]}=(|{\varphi }_{k}^{+},{\varphi }_{k}^{+}\rangle \langle {\varphi }_{k}^{+},{\varphi }_{k}^{+}|+|{\varphi }_{k}^{-},{\varphi }_{k}^{-}\rangle \langle {\varphi }_{k}^{-},{\varphi }_{k}^{-}|)/2$ = $({1}_{2}\otimes {1}_{2}+{\hat{\sigma }}_{k}\otimes {\hat{\sigma }}_{k})/4$, respectively^5^. Now, ref.^5^ used the pure state $|\psi \rangle ={\mathscr{N}}\,({e}^{i{\delta }_{1}}|{{\rm{\Phi }}}_{1}\rangle +{e}^{i{\delta }_{2}}|{{\rm{\Phi }}}_{2}\rangle +{e}^{i{\delta }_{3}}|{{\rm{\Phi }}}_{3}\rangle )$, where ${\mathscr{N}}$ is the normalization constant and {*δ*_*k*_} are adjustable local phases. We here intend to estimate the three components of the magnetic field using a mixed state ${\hat{\rho }}^{N}$, obtained from the above pure state in the presence of local dephasing, described using two single-particle Kraus operators^24^,

21 $${\hat{E}}_{0}=[\begin{array}{cc}1 & 0\\ 0 & {e}^{-\lambda }\end{array}],\,{\hat{E}}_{1}=[\begin{array}{cc}0 & 0\\ 0 & \sqrt{1-{e}^{-2\lambda }}\end{array}],$$

where *λ* is some constant causing the phase damping, such that the off-diagonal elements of the density matrix decay exponentially to zero with time. Considering that all particles dephase uniformly, the *N*-particle density matrix of the desired mixed state is then^59^:

$${\hat{\rho }}^{N}=\sum _{g=0}^{N}\,\sum _{{\pi }_{g}^{N}}\,{\pi }_{g}^{N}\,[{\hat{E}}_{1}^{\otimes g}\otimes {\hat{E}}_{0}^{\otimes N-g}]\,|\psi \rangle \langle \psi |\,{\pi }_{g}^{N}\,[{\hat{E}}_{1}^{\dagger \otimes g}\otimes {\hat{E}}_{0}^{\dagger \otimes N-g}],$$

where ${\pi }_{g}^{N}$ represents different permutations of *g* and *N* − *g* copies of the ${\hat{E}}_{1}$ and ${\hat{E}}_{0}$ operators, respectively. Note that the operators ${\hat{E}}_{0}$ and ${\hat{E}}_{1}$ are Hermitian, so the †s can be dropped. Clearly, these states ${\hat{\rho }}^{N}$ are permutationally invariant as well, with now first and second order reduced density matrices ${\hat{\rho }}^{[1]}={1}_{2}/2$ and ${\hat{\rho }}_{k}^{[2]}$ = $[{1}_{2}\otimes {1}_{2}+({\sum }_{r=0}^{1}\,{\hat{E}}_{r}{\hat{\sigma }}_{k}{\hat{E}}_{r})\otimes ({\sum }_{s=0}^{1}\,{\hat{E}}_{s}{\hat{\sigma }}_{k}{\hat{E}}_{s})]/4$, respectively. This is shown in Section III of Supplementary Information.

For *N* = 8*n*, $$n\in {\mathbb{N}}$$ (and *δ*_*k*_ = 0 for all *k*), the two-body reduced density matrix of $${\hat{\rho }}^{N}$$ is an equal mixture of those in all directions (as in the pure state case in ref. ^5^), given by $${\hat{\rho }}^{[2]}$$ = $$\frac{1}{3}\,{\sum }_{k=1}^{3}\,{\hat{\rho }}_{k}^{[2]}$$ = $$\frac{1}{4}{1}_{2}\otimes {1}_{2}+\frac{1}{12}\,{\sum }_{k=1}^{3}$$$$[({\sum }_{r=0}^{1}\,{\hat{E}}_{r}{\hat{\sigma }}_{k}{\hat{E}}_{r})\otimes ({\sum }_{s=0}^{1}\,{\hat{E}}_{s}{\hat{\sigma }}_{k}{\hat{E}}_{s})]$$. For any other *N*, the difference from the form of $${\hat{\rho }}^{\mathrm{[2]}}$$ is exponentially small in *N*. This directly follows from the way it was shown in ref. ^5^ for the pure state case. Hence, we consider the probe state to have marginals $${\hat{\rho }}^{[1]}={1}_{2}/2$$ and $${\hat{\rho }}^{[2]}$$ as above and calculate the QFIM. We get

22 $${J}_{Q}^{jk,[1]}=2\,{\rm{T}}{\rm{r}}\,[{\hat{b}}_{j}{\hat{b}}_{k}],$$

and

23 $$\begin{array}{ccc}{J}_{Q}^{jk,[2]} & = & \frac{1}{3}\,\sum _{t=1}^{3}\,{\rm{T}}{\rm{r}}\,[(\sum _{r=0}^{1}\,{\hat{E}}_{r}{\hat{\sigma }}_{t}{\hat{E}}_{r}\otimes \sum _{s=0}^{1}\,{\hat{E}}_{s}{\hat{\sigma }}_{t}{\hat{E}}_{s})\,({\hat{b}}_{j}\otimes {\hat{b}}_{k})]\\  & = & \frac{1}{3}\,\sum _{t=1}^{3}\,{\rm{T}}{\rm{r}}\,[\sum _{r=0}^{1}\,{\hat{E}}_{r}{\hat{\sigma }}_{t}{\hat{E}}_{r}{\hat{b}}_{j}]\,{\rm{T}}{\rm{r}}\,[\sum _{s=0}^{1}\,{\hat{E}}_{s}{\hat{\sigma }}_{t}{\hat{E}}_{s}{\hat{b}}_{k}]\\  & = & \frac{1}{3}\,\sum _{t=1}^{3}\,{\rm{T}}{\rm{r}}\,[{\hat{\sigma }}_{t}\,\sum _{r=0}^{1}\,{\hat{E}}_{r}{\hat{b}}_{j}{\hat{E}}_{r}]\,{\rm{T}}{\rm{r}}\,[{\hat{\sigma }}_{t}\,\sum _{s=0}^{1}\,{\hat{E}}_{s}{\hat{b}}_{k}{\hat{E}}_{s}]\\  & = & \frac{2}{3}\,{\rm{T}}{\rm{r}}\,[\sum _{t=1}^{3}\,{\rm{T}}{\rm{r}}\,[{\hat{\sigma }}_{t}\,(\sum _{r=0}^{1}\,{\hat{E}}_{r}{\hat{b}}_{j}{\hat{E}}_{r})]\,{\hat{\sigma }}_{t}\,(\sum _{s=0}^{1}\,{\hat{E}}_{s}{\hat{b}}_{k}{\hat{E}}_{s})]\\  & = & \frac{2}{3}\,{\rm{T}}{\rm{r}}\,[(\sum _{r=0}^{1}\,{\hat{E}}_{r}{\hat{b}}_{j}{\hat{E}}_{r})\,(\sum _{s=0}^{1}\,{\hat{E}}_{s}{\hat{b}}_{k}{\hat{E}}_{s})].\end{array}$$

Define ${\hat{f}}_{j}={\sum }_{r=0}^{1}\,{\hat{E}}_{r}{\hat{b}}_{j}{\hat{E}}_{r}$ and ${\hat{f}}_{k}={\sum }_{s=0}^{1}\,{\hat{E}}_{s}{\hat{b}}_{k}{\hat{E}}_{s}$. Also, let $\xi =\sqrt{{\theta }_{1}^{2}+{\theta }_{2}^{2}+{\theta }_{3}^{2}}$, ${\eta }_{k}=\frac{{\theta }_{k}}{\sqrt{{\theta }_{1}^{2}+{\theta }_{2}^{2}+{\theta }_{3}^{2}}}$ for all *k* = 1, 2, 3 (corresponding to the *X*, *Y* and *Z* directions). Here, (22) is found to be (following ref.^5^):

24 $${J}_{Q}^{jk,[1]}=4\,[(1-{{\rm{s}}{\rm{i}}{\rm{n}}{\rm{c}}}^{2}[\xi ])\,{\eta }_{j}{\eta }_{k}+{\delta }_{jk}\,{{\rm{s}}{\rm{i}}{\rm{n}}{\rm{c}}}^{2}[\xi ]],$$

where sinc[*ξ*] = sin[*ξ*]/*ξ*. From (18), (24), (23), we get:

25 $$\begin{array}{ccc}{J}_{Q}^{jk} & = & 4N\,[(1-{{\rm{s}}{\rm{i}}{\rm{n}}{\rm{c}}}^{2}[\xi ])\,{\eta }_{j}{\eta }_{k}+{\delta }_{jk}\,{{\rm{s}}{\rm{i}}{\rm{n}}{\rm{c}}}^{2}[\xi ]]\,+\,\frac{2N(N-1)}{3}\,{\rm{T}}{\rm{r}}\,[{\hat{f}}_{j}{\hat{f}}_{k}],\end{array}$$

where the terms ${\rm{Tr}}\,[{\hat{f}}_{j}{\hat{f}}_{k}]$ can be explicitly calculated.

Since some or all of the terms ${\rm{Tr}}\,[{\hat{b}}_{j}{\hat{b}}_{k}]$ are non-zero, we can have the terms ${\rm{Tr}}\,[{\hat{f}}_{j}{\hat{f}}_{k}]$ as non-zero, such that the scaling 1/*N* can be achieved, as the parallel scheme bound without ancillas from ref.^31^ can be tight even for *β* ≠ 0. Even when ${\rm{Tr}}\,[{\hat{b}}_{j}{\hat{b}}_{k}]=0$, the terms ${\rm{Tr}}\,[{\hat{f}}_{j}{\hat{f}}_{k}]$ in general (i.e. when ${\hat{E}}_{0}$ and ${\hat{E}}_{1}$ need not be local dephasing operators), can be non-zero. This implies that it is possible to achieve the Heisenberg scaling with the presence of noise in the initial probe state, even when such a scaling cannot be achieved in the absence of noise in the initial state. This is because mixed separable states can be as nonclassical as entangled pure states^60^. Thus, noise in the initial probe state can act as a feature rather than a bug in attaining the Heisenberg limit. Note though that it is unlikely for all the terms ${\rm{Tr}}\,[{\hat{b}}_{j}{\hat{b}}_{k}]$ to be zero, since that would mean that the QFIM *J*_*Q*_ is zero for the pure state case from ref. ^5^. However, even when some or all of the terms ${\rm{Tr}}\,[{\hat{b}}_{j}{\hat{b}}_{k}]$ are non-zero, it may be possible for the terms ${\rm{Tr}}\,[{\hat{f}}_{j}{\hat{f}}_{k}]$ to be such that the QFIM *J*_*Q*_ for the mixed state case considered here is larger than that for the pure state case from ref. ^5^. This is because mixed entangled states can be more nonclassical than pure entangled states^60^. Thus, noise in the initial probe state can allow for better estimation precision than the case of no noise in the initial state.

Although noise is known to reduce quantum correlations in a system in most cases^24,61^, noise can also introduce or increase quantum correlations in a system^62–65^. For example, local dephasing considered in this section is a local unital noise^64^, that mostly decreases quantum correlations. Instead, if local non-unital noise, such as local dissipation^65^ as represented by the following single-particle Kraus operators^24^, is used to obtain the initial mixed probe state from a classically correlated separable state, the mixed state so obtained can have quantum correlations, that may be activated into entanglement, allowing for better estimation precision^66–69^:

26 $${\hat{E}}_{0}=[\begin{array}{cc}1 & 0\\ 0 & \sqrt{1-{e}^{-2\kappa }}\end{array}],\,{\hat{E}}_{1}=[\begin{array}{cc}0 & {e}^{-\kappa }\\ 0 & 0\end{array}],$$

where *κ* is a constant causing amplitude damping. This is why ancilla-assisted schemes of ref.^31^ yielded scaling better than that without ancillas for amplitude damping.

Nonetheless, if ${\hat{\rho }}^{[2]}={\hat{\rho }}^{[1]}\otimes {\hat{\rho }}^{[1]}={1}_{4}/4$, i.e. both ${\hat{\rho }}^{[1]}$ and ${\hat{\rho }}^{[2]}$ are maximally mixed, then ${\rm{Tr}}\,[{\hat{f}}_{j}{\hat{f}}_{k}]=0$, since ${\sum }_{t=1}^{3}\,({\sum }_{r=0}^{1}\,{\hat{E}}_{r}{\hat{\sigma }}_{t}{\hat{E}}_{r}\otimes {\sum }_{s=0}^{1}\,{\hat{E}}_{s}{\hat{\sigma }}_{t}{\hat{E}}_{s})$ would be zero in (23), such that the best scaling achievable is $1/\sqrt{N}$. Thus, unlike the conventional wisdom that any amount of noise is harmful, we see that some amount of noise in the initial probe state can be useful and provides a quantum advantage through its quantum correlations, but a lot of noise is harmful because of too much quantum correlations in the state.

## Noisy Quantum Channel

We consider a general noisy quantum channel that allows the state $\hat{\rho }$ to evolve not necessarily unitarily. Let ${\hat{{\rm{\Pi }}}}_{l}({\boldsymbol{\theta }})$ be the Kraus operators that describe the dynamical evolution of the system. The state of the system after the evolution is^22,26^

27 $$\hat{\rho }({\boldsymbol{\theta }})=\sum _{l}\,{\hat{{\rm{\Pi }}}}_{l}({\boldsymbol{\theta }})\hat{\rho }{\hat{{\rm{\Pi }}}}_{l}^{\dagger }({\boldsymbol{\theta }}),$$

where ${\sum }_{l}\,{\hat{{\rm{\Pi }}}}_{l}^{\dagger }({\boldsymbol{\theta }}){\hat{{\rm{\Pi }}}}_{l}({\boldsymbol{\theta }})=1$. Even when the transformation (27) is non-unitary, it may be described by a unitary evolution ${\hat{U}}_{SB}({\boldsymbol{\theta }})$ in a bigger space, comprising the system *S* and some vacuum state ancillary bath *B*. The evolved state in *S* + *B* space is given by

28 $$\begin{array}{c}{\hat{\rho }}_{SB}({\boldsymbol{\theta }})={\hat{U}}_{SB}({\boldsymbol{\theta }})\,(\hat{\rho }\otimes |0\rangle \langle 0|)\,{\hat{U}}_{SB}^{\dagger }({\boldsymbol{\theta }})=\sum _{l,v}\,{\hat{{\rm{\Pi }}}}_{l}({\boldsymbol{\theta }})\hat{\rho }{\hat{{\rm{\Pi }}}}_{v}^{\dagger }({\boldsymbol{\theta }})\otimes |l\rangle \langle v|\end{array}$$

Then, following from (13), (14), (15) for the noiseless *S* + *B* space, we get

29 $$\begin{array}{c}\frac{{\rm{\partial }}}{{\rm{\partial }}{\theta }_{k}}{\hat{\rho }}_{SB}({\boldsymbol{\theta }})=-\,i[{\hat{M}}_{k}({\boldsymbol{\theta }}),{\hat{\rho }}_{SB}({\boldsymbol{\theta }})]=\frac{1}{2}\,({\hat{L}}_{k}{\hat{\rho }}_{SB}({\boldsymbol{\theta }})+{\hat{\rho }}_{SB}({\boldsymbol{\theta }}){\hat{L}}_{k}^{\dagger }),\end{array}$$

where ${\hat{M}}_{k}({\boldsymbol{\theta }})\equiv i\frac{\partial {\hat{U}}_{SB}({\boldsymbol{\theta }})}{\partial {\theta }_{k}}{\hat{U}}_{SB}^{\dagger }({\boldsymbol{\theta }})$, and ${\hat{L}}_{k}=-\,2i{\rm{\Delta }}{\hat{M}}_{k}$ is anti-Hermitian, ${\rm{\Delta }}{\hat{M}}_{k}\equiv {\hat{M}}_{k}({\boldsymbol{\theta }})-{\rm{Tr}}\,({\hat{M}}_{k}({\boldsymbol{\theta }}){\hat{\rho }}_{SB}({\boldsymbol{\theta }}))$. Then, the QFIM from (6) for ${\hat{\rho }}_{SB}({\boldsymbol{\theta }})$ takes the form:

30 $$\begin{array}{ccc}{J}_{Q}^{jk} & = & 4{\rm{R}}{\rm{e}}\,[{\rm{T}}{\rm{r}}\,({\hat{H}}_{1}^{jk}({\boldsymbol{\theta }})\,(\hat{\rho }\otimes |0\rangle \langle 0|))\,-\,{\rm{T}}{\rm{r}}\,({\hat{H}}_{2}^{j}({\boldsymbol{\theta }})\,(\hat{\rho }\otimes |0\rangle \langle 0|))\,{\rm{T}}{\rm{r}}\,({\hat{H}}_{2}^{k}({\boldsymbol{\theta }})\,(\hat{\rho }\otimes |0\rangle \langle 0|))],\end{array}$$

where

31 $$\begin{array}{rcl}{\hat{H}}_{1}^{jk}({\boldsymbol{\theta }}) & = & \frac{\partial {\hat{U}}_{SB}^{\dagger }({\boldsymbol{\theta }})}{\partial {\theta }_{j}}\frac{\partial {\hat{U}}_{SB}({\boldsymbol{\theta }})}{\partial {\theta }_{k}},\\ {\hat{H}}_{2}^{k}({\boldsymbol{\theta }}) & = & i\frac{\partial {\hat{U}}_{SB}^{\dagger }({\boldsymbol{\theta }})}{\partial {\theta }_{k}}{\hat{U}}_{SB}({\boldsymbol{\theta }}).\end{array}$$

However, when only the system *S* is monitored but the bath *B* is not monitored, we recover (27) from (28) by taking a partial trace with respect to *B*: ${{\rm{Tr}}}_{B}\,({\hat{\rho }}_{SB}({\boldsymbol{\theta }}))=\hat{\rho }({\boldsymbol{\theta }})$. Then, if we trace out the bath *B* before having the traces in (30), we obtain an upper bound (like those obtained in refs ^22,26^) to the QFIM in (6) for $\hat{\rho }({\boldsymbol{\theta }})$:

32 $${C}_{Q}^{jk}=4\,{\rm{R}}{\rm{e}}\,[{\rm{T}}{\rm{r}}\,({\hat{K}}_{1}^{jk}({\boldsymbol{\theta }})\hat{\rho })-{\rm{T}}{\rm{r}}\,({\hat{K}}_{2}^{j}({\boldsymbol{\theta }})\hat{\rho })\,{\rm{T}}{\rm{r}}\,({\hat{K}}_{2}^{k}({\boldsymbol{\theta }})\hat{\rho })],$$

where

33 $$\begin{array}{rcl}{\hat{K}}_{1}^{jk}({\boldsymbol{\theta }}) & = & \sum _{l}\,\frac{\partial {\hat{{\rm{\Pi }}}}_{l}^{\dagger }({\boldsymbol{\theta }})}{\partial {\theta }_{j}}\frac{\partial {\hat{{\rm{\Pi }}}}_{l}({\boldsymbol{\theta }})}{\partial {\theta }_{k}},\\ {\hat{K}}_{2}^{k}({\boldsymbol{\theta }}) & = & i\,\sum _{p}\,\frac{\partial {\hat{{\rm{\Pi }}}}_{p}^{\dagger }({\boldsymbol{\theta }})}{\partial {\theta }_{k}}{\hat{{\rm{\Pi }}}}_{p}({\boldsymbol{\theta }}),\end{array}$$

such that

34 $$\begin{array}{ccc}{\hat{K}}_{1}^{jk}({\boldsymbol{\theta }})\hat{\rho } & = & {{\rm{T}}{\rm{r}}}_{B}\,[{\hat{H}}_{1}^{jk}({\boldsymbol{\theta }})\,(\hat{\rho }\otimes |0\rangle \langle 0|)],\\ {\hat{K}}_{2}^{k}({\boldsymbol{\theta }})\hat{\rho } & = & {{\rm{T}}{\rm{r}}}_{B}\,[{\hat{H}}_{2}^{k}({\boldsymbol{\theta }})\,(\hat{\rho }\otimes |0\rangle \langle 0|)].\end{array}$$

We prove in Section IV of Supplementary Information that *C*_*Q*_ from (32) is an upper bound to the QFIM *J*_*Q*_ from (6) for $\hat{\rho }({\boldsymbol{\theta }})$. An outline of the proof is given in the Methods section.

One may compare these results with those in ref.^22^, where initially pure states in different modes were assumed to evolve independently. We made no such assumption and our initial state is mixed, and so our results are more general. Also, we consider estimation of multiple parameters, as opposed to single parameter estimation studied in ref.^26^. Our upper bound to the QFIM is relevant, since there are an infinitude of Kraus representations ${\hat{{\rm{\Pi }}}}_{l}({\boldsymbol{\theta }})$ of the channel that make the bound to equal the QFIM^26^.

Now, we claim that (32) is saturated, when the following condition is satisfied:

35 $${\rm{I}}{\rm{m}}\,[\sum _{l}\,{\rm{T}}{\rm{r}}\,\{(\frac{{\rm{\partial }}{\hat{{\rm{\Pi }}}}_{l}^{\dagger }({\boldsymbol{\theta }})}{{\rm{\partial }}{\theta }_{j}}\frac{{\rm{\partial }}{\hat{{\rm{\Pi }}}}_{l}({\boldsymbol{\theta }})}{{\rm{\partial }}{\theta }_{k}})\,\hat{\rho }\}]=0\,{\rm{\forall }}j,k,$$

which is obtained from (8) for *S* + *B* space, by tracing out *B* (see Methods section). That is, the bound (32) is saturated, when the expectation, with respect to the initial probe state, of the commutator of every pair of the derivatives of the channel Kraus operator and its adjoint vanishes. Clearly, when the above condition is satisfied, it is possible to attain the elusive Heisenberg limit even in the most general noisy estimation scenario. One can observe that the above condition is trivially true for single parameter estimation.

Then, the set of POVMs of cardinality *q* + 2, comprising the following *q* + 1 elements,

36 $$\begin{array}{ccc}{\hat{P}}_{0} & = & \hat{\rho }({\boldsymbol{\theta }})=\sum _{l}\,{\hat{{\rm{\Pi }}}}_{l}({\boldsymbol{\theta }})\hat{\rho }{\hat{{\rm{\Pi }}}}_{l}^{\dagger }({\boldsymbol{\theta }}),\\ {\hat{P}}_{m} & = & \frac{{\rm{\partial }}\hat{\rho }({\boldsymbol{\theta }})}{{\rm{\partial }}{\theta }_{m}}=\sum _{l}\,[\frac{{\rm{\partial }}{\hat{{\rm{\Pi }}}}_{l}({\boldsymbol{\theta }})}{{\rm{\partial }}{\theta }_{m}}\hat{\rho }{\hat{{\rm{\Pi }}}}_{l}^{\dagger }({\boldsymbol{\theta }})\,+\,{\hat{{\rm{\Pi }}}}_{l}({\boldsymbol{\theta }})\hat{\rho }\frac{{\rm{\partial }}{\hat{{\rm{\Pi }}}}_{l}^{\dagger }({\boldsymbol{\theta }})}{{\rm{\partial }}{\theta }_{m}}]\,{\rm{\forall }}m=1,\ldots ,q,\end{array}$$

along with one element accounting for normalisation, saturates (32) (see Methods section).

## Upper Bound to the QFIM for *N* Particles Evolving through Noisy Channel

Consider that the probe comprising *N* particles evolves not necessarily unitarily. Then, the QFIM (16) is for unitary evolution of a probe comprising more than *N* particles in *S* + *B* space. The evolution of the probe comprising *N* particles in *S* space alone is described here by some unital Kraus operators ${\hat{{\rm{\Pi }}}}_{l}({\boldsymbol{\theta }})=\frac{1}{\sqrt{L}}{e}^{-i{\hat{G}}_{l}({\boldsymbol{\theta }})}$, where *l* = 1, …, *L*, ${\sum }_{l}\,{\hat{{\rm{\Pi }}}}_{l}^{\dagger }({\boldsymbol{\theta }}){\hat{{\rm{\Pi }}}}_{l}({\boldsymbol{\theta }})={\sum }_{l}\,{\hat{{\rm{\Pi }}}}_{l}({\boldsymbol{\theta }}){\hat{{\rm{\Pi }}}}_{l}^{\dagger }({\boldsymbol{\theta }})=1$, and

37 $${\hat{G}}_{l}({\boldsymbol{\theta }})=\sum _{n=1}^{N}\,{\hat{\pi }}_{{l}_{n}}^{[n]}=\sum _{k=1}^{q}\,{\theta }_{k}\,\sum _{n=1}^{N}\,{\hat{\pi }}_{{l}_{n}k}^{[n]}\equiv \sum _{k=1}^{q}\,{\theta }_{k}{\hat{G}}_{lk}.$$

The generators ${\hat{G}}_{lk}$ do not depend on ***θ*** and do not generally commute with each other. Then, as with unitary operators considered earlier,

38 $$\frac{\partial {\hat{{\rm{\Pi }}}}_{l}({\boldsymbol{\theta }})}{\partial {\theta }_{k}}=\frac{-i}{L\sqrt{L}}\,{\int }_{0}^{1}\,d\alpha {e}^{-i(1-\alpha ){\hat{G}}_{l}({\boldsymbol{\theta }})}\frac{\partial {\hat{G}}_{l}({\boldsymbol{\theta }})}{\partial {\theta }_{k}}{e}^{-i\alpha {\hat{G}}_{l}({\boldsymbol{\theta }})}.$$

So, ${{\rm{Tr}}}_{B}\,[{\hat{M}}_{k}({\boldsymbol{\theta }})]$ = $i\,{\sum }_{l}\,\frac{\partial {\hat{{\rm{\Pi }}}}_{l}({\boldsymbol{\theta }})}{\partial {\theta }_{k}}{\hat{{\rm{\Pi }}}}_{l}^{\dagger }({\boldsymbol{\theta }})$ = ${\sum }_{l}\,{\hat{{\rm{\Pi }}}}_{l}({\boldsymbol{\theta }}){\hat{B}}_{lk}({\boldsymbol{\theta }}){\hat{{\rm{\Pi }}}}_{l}^{\dagger }({\boldsymbol{\theta }})$, where ${\hat{M}}_{k}({\boldsymbol{\theta }})$ is from (14) for *S* + *B* space, ${\sum }_{l}\,{\hat{B}}_{lk}({\boldsymbol{\theta }})$ = ${{\rm{Tr}}}_{B}\,[{\hat{A}}_{k}({\boldsymbol{\theta }})]$ = $\frac{1}{L}\,{\sum }_{l}\,{\int }_{0}^{1}\,d\alpha {e}^{i\alpha {\hat{G}}_{l}({\boldsymbol{\theta }})}{\hat{G}}_{lk}{e}^{-i\alpha {\hat{G}}_{l}({\boldsymbol{\theta }})}$, since we have $\frac{\partial {\hat{G}}_{l}({\boldsymbol{\theta }})}{\partial {\theta }_{k}}={\hat{G}}_{lk}$. Then, we have in *S* + *B* space

39 $$\frac{\partial {\hat{U}}_{SB}^{\dagger }({\boldsymbol{\theta }})}{\partial {\theta }_{j}}\frac{\partial {\hat{U}}_{SB}({\boldsymbol{\theta }})}{\partial {\theta }_{k}}={\hat{A}}_{j}({\boldsymbol{\theta }}){\hat{U}}_{SB}^{\dagger }({\boldsymbol{\theta }}){\hat{U}}_{SB}({\boldsymbol{\theta }}){\hat{A}}_{k}({\boldsymbol{\theta }}).$$

Tracing out the bath *B*, we get (see Section X of Supplementary Information to understand why an extra 1/*L* does not arise below):

40 $$\begin{array}{cccc} & \sum _{l}\,\frac{{\rm{\partial }}{\hat{{\rm{\Pi }}}}_{l}^{\dagger }({\boldsymbol{\theta }})}{{\rm{\partial }}{\theta }_{j}}\frac{{\rm{\partial }}{\hat{{\rm{\Pi }}}}_{l}({\boldsymbol{\theta }})}{{\rm{\partial }}{\theta }_{k}} & = & \sum _{l}\,{\hat{B}}_{lj}({\boldsymbol{\theta }}){\hat{{\rm{\Pi }}}}_{l}^{\dagger }({\boldsymbol{\theta }}){\hat{{\rm{\Pi }}}}_{l}({\boldsymbol{\theta }}){\hat{B}}_{lk}({\boldsymbol{\theta }})\\ \Rightarrow  & {\rm{T}}{\rm{r}}\,[\sum _{l}\,{\textstyle \tfrac{{\rm{\partial }}{\hat{{\rm{\Pi }}}}_{l}^{\dagger }({\boldsymbol{\theta }})}{{\rm{\partial }}{\theta }_{j}}}{\textstyle \tfrac{{\rm{\partial }}{\hat{{\rm{\Pi }}}}_{l}({\boldsymbol{\theta }})}{{\rm{\partial }}{\theta }_{k}}}] & = & {\rm{T}}{\rm{r}}\,[\sum _{l}\,{\textstyle \tfrac{{\rm{\partial }}{\hat{{\rm{\Pi }}}}_{l}^{\dagger }({\boldsymbol{\theta }})}{{\rm{\partial }}{\theta }_{j}}}{\hat{{\rm{\Pi }}}}_{l}({\boldsymbol{\theta }}){\hat{{\rm{\Pi }}}}_{l}^{\dagger }({\boldsymbol{\theta }}){\textstyle \tfrac{{\rm{\partial }}{\hat{{\rm{\Pi }}}}_{l}({\boldsymbol{\theta }})}{{\rm{\partial }}{\theta }_{k}}}]\\  &  & = & {\rm{T}}{\rm{r}}\,[\sum _{l}\,{\hat{B}}_{lj}({\boldsymbol{\theta }}){\hat{{\rm{\Pi }}}}_{l}^{\dagger }({\boldsymbol{\theta }}){\hat{{\rm{\Pi }}}}_{l}({\boldsymbol{\theta }}){\hat{{\rm{\Pi }}}}_{l}^{\dagger }({\boldsymbol{\theta }}){\hat{{\rm{\Pi }}}}_{l}({\boldsymbol{\theta }}){\hat{B}}_{lk}({\boldsymbol{\theta }})],\end{array}$$

since ${\sum }_{l}\,{\hat{{\rm{\Pi }}}}_{l}({\boldsymbol{\theta }}){\hat{{\rm{\Pi }}}}_{l}^{\dagger }({\boldsymbol{\theta }})=1$. Also, we have in *S* + *B* space

41 $$i\frac{\partial {\hat{U}}_{SB}^{\dagger }({\boldsymbol{\theta }})}{\partial {\theta }_{k}}{\hat{U}}_{SB}({\boldsymbol{\theta }})=-\,{\hat{A}}_{k}({\boldsymbol{\theta }}){\hat{U}}_{SB}^{\dagger }({\boldsymbol{\theta }}){\hat{U}}_{SB}({\boldsymbol{\theta }}).$$

Again, tracing out the bath *B*, we get:

42 $$i\,\sum _{l}\,\frac{\partial {\hat{{\rm{\Pi }}}}_{l}^{\dagger }({\boldsymbol{\theta }})}{\partial {\theta }_{k}}{\hat{{\rm{\Pi }}}}_{l}({\boldsymbol{\theta }})=-\,\sum _{l}\,{\hat{B}}_{lk}({\boldsymbol{\theta }}){\hat{{\rm{\Pi }}}}_{l}^{\dagger }({\boldsymbol{\theta }}){\hat{{\rm{\Pi }}}}_{l}({\boldsymbol{\theta }}).$$

Then, we get the desired upper bound *C*_*Q*_ to the QFIM from (16) as follows:

43 $$\begin{array}{ccc}{C}_{Q}^{jk} & = & 4\,{\rm{R}}{\rm{e}}\,[{\rm{T}}{\rm{r}}\,(\sum _{l}\,{\hat{B}}_{lj}({\boldsymbol{\theta }}){\hat{B}}_{lk}({\boldsymbol{\theta }})\hat{\rho })\,-\,{\rm{T}}{\rm{r}}\,(\sum _{p}\,{\hat{B}}_{pj}({\boldsymbol{\theta }})\hat{\rho })\,{\rm{T}}{\rm{r}}\,(\sum _{r}\,{\hat{B}}_{rk}({\boldsymbol{\theta }})\hat{\rho })],\end{array}$$

since ${\sum }_{l}\,{\hat{{\rm{\Pi }}}}_{l}^{\dagger }({\boldsymbol{\theta }}){\hat{{\rm{\Pi }}}}_{l}({\boldsymbol{\theta }})=1$. Also, here we have

$$\sum _{l}\,{\hat{B}}_{lk}({\boldsymbol{\theta }})=\sum _{n}\,\sum _{{l}_{n}}\,{\hat{d}}_{{l}_{n}k}^{[n]}=\frac{1}{L}\,\sum _{n}\,\sum _{{l}_{n}}\,{\int }_{0}^{1}\,d\alpha {e}^{i\alpha {\hat{\pi }}_{{l}_{n}}^{[n]}}{\hat{\pi }}_{{l}_{n}k}^{[n]}{e}^{-i\alpha {\hat{\pi }}_{{l}_{n}}^{[n]}}.$$

Thus, (43) becomes:

$$\begin{array}{ccc}{C}_{Q}^{jk} & = & 4\,\sum _{n}\,{\rm{R}}{\rm{e}}\,[{\rm{T}}{\rm{r}}\,[\sum _{{l}_{n}}\,{\hat{\rho }}^{[n]}{\hat{d}}_{{l}_{n}j}^{[n]}{\hat{d}}_{{l}_{n}k}^{[n]}]\,-\,{\rm{T}}{\rm{r}}\,[\sum _{{l}_{{n}_{1}}}\,{\hat{\rho }}^{[n]}{\hat{d}}_{{l}_{{n}_{1}}j}^{[n]}]\,{\rm{T}}{\rm{r}}\,[\sum _{{l}_{{n}_{2}}}\,{\hat{\rho }}^{[n]}{\hat{d}}_{{l}_{{n}_{2}}k}^{[n]}]]\\  &  & +\,4\,\sum _{n\ne m}\,\sum _{{l}_{n},{l}_{m}}\,{\rm{R}}{\rm{e}}\,[{\rm{T}}{\rm{r}}\,[{\hat{\rho }}^{[n,m]}\,({\hat{d}}_{{l}_{n}j}^{[n]}\otimes {\hat{d}}_{{l}_{m}k}^{[m]})]\,-\,{\rm{T}}{\rm{r}}\,[{\hat{\rho }}^{[n]}{\hat{d}}_{{l}_{n}j}^{[n]}]\,{\rm{T}}{\rm{r}}\,[{\hat{\rho }}^{[m]}{\hat{d}}_{{l}_{m}k}^{[m]}]]\\  & = & \sum _{n}\,{C}_{Q}^{jk,[1]}\,({\hat{\rho }}^{[n]})+\sum _{n\ne m}\,{C}_{Q}^{jk,[2]}\,({\hat{\rho }}^{[n,m]}),\end{array}$$

where ${C}_{Q}^{jk,[1]}$ depends only on one-particle reduced density matrix on subsystem *n* and ${C}_{Q}^{jk,[2]}$ depends on two-particle reduced density matrix on subsystems *n*, *m*.

Further, if we restrict ourselves to only permutationally invariant states, the upper bound to the QFIM from (18) is as follows:

44 $${C}_{Q}^{jk}=N{C}_{Q}^{jk,[1]}\,({\hat{\rho }}^{[1]})+N(N-1){C}_{Q}^{jk,[2]}\,({\hat{\rho }}^{[1]},{\hat{\rho }}^{[2]}),$$

where

$${C}_{Q}^{jk,[1]}=4\,\sum _{p,r}\,{\rm{R}}{\rm{e}}\,[{\rm{T}}{\rm{r}}\,[{\hat{\rho }}^{[1]}{\hat{d}}_{pj}{\hat{d}}_{pk}]-{\rm{T}}{\rm{r}}\,[{\hat{\rho }}^{[1]}{\hat{d}}_{pj}]\,{\rm{T}}{\rm{r}}\,[{\hat{\rho }}^{[1]}{\hat{d}}_{rk}]]$$

only depends on the first order reduced density matrix,

$$\begin{array}{ccc}{C}_{Q}^{jk,[2]} & = & 4\,\sum _{p,r}\,{\rm{R}}{\rm{e}}\,[{\rm{T}}{\rm{r}}\,[{\hat{\rho }}^{[2]}\,({\hat{d}}_{pj}\otimes {\hat{d}}_{rk})]\,-\,{\rm{T}}{\rm{r}}\,[{\hat{\rho }}^{[1]}{\hat{d}}_{pj}]\,{\rm{T}}{\rm{r}}\,[{\hat{\rho }}^{[1]}{\hat{d}}_{rk}]]\end{array}$$

also depends on the second order reduced density matrix.

Clearly, when the two-particle reduced density matrix of the initial probe state is a product state, we get ${C}_{Q}^{jk,[2]}=0$. When both the one- and two-particle reduced density matrices of the initial probe state are maximally mixed, we again get ${C}_{Q}^{jk,[2]}=0$. Thus, a precision scaling of 1/*N* cannot be achieved, when there are no correlations or too much quantum correlations in the initial state, like in unitary channel case. Thus, any quantum enhancement to the estimation precision is provided by the two-particle reduced density matrices of the probe state.

Now, from (36), the set of POVMs comprising

45 $$\begin{array}{ccc}{\hat{P}}_{0} & = & \hat{\rho }({\boldsymbol{\theta }})=\sum _{l}\,{\hat{{\rm{\Pi }}}}_{l}({\boldsymbol{\theta }})\hat{\rho }{\hat{{\rm{\Pi }}}}_{l}^{\dagger }({\boldsymbol{\theta }}),\\ {\hat{P}}_{m} & = & \frac{{\rm{\partial }}\hat{\rho }({\boldsymbol{\theta }})}{{\rm{\partial }}{\theta }_{m}}=\sum _{l}\,[{\hat{{\rm{\Pi }}}}_{l}({\boldsymbol{\theta }}){\hat{B}}_{lm}({\boldsymbol{\theta }})\hat{\rho }{\hat{{\rm{\Pi }}}}_{l}^{\dagger }({\boldsymbol{\theta }})\,+\,{\hat{{\rm{\Pi }}}}_{l}({\boldsymbol{\theta }})\hat{\rho }{\hat{B}}_{lm}({\boldsymbol{\theta }}){\hat{{\rm{\Pi }}}}_{l}^{\dagger }({\boldsymbol{\theta }})]\,{\rm{\forall }}m=1,\ldots ,q,\end{array}$$

along with one element accounting for normalisation, saturates the upper bound (43) to the QFIM, provided we have (35), i.e. here

46 $$\begin{array}{cccc} & 4\,\sum _{l}\,{\rm{I}}{\rm{m}}\,[{\rm{T}}{\rm{r}}\,({\hat{B}}_{lj}(\theta ){\hat{B}}_{lk}(\theta )\hat{\rho })] & = & 0\,{\rm{\forall }}j,k\\ \Rightarrow  & 4\,\sum _{n}\,{\rm{I}}{\rm{m}}\,{\rm{T}}{\rm{r}}\,[\sum _{{l}_{n}}\,{\hat{\rho }}^{[n]}{\hat{d}}_{{l}_{n}j}^{[n]}{\hat{d}}_{{l}_{n}k}^{[n]}]\,+\,4\,\sum _{n\ne m}\,\sum _{{l}_{n},{l}_{m}}\,{\rm{I}}{\rm{m}}\,{\rm{T}}{\rm{r}}\,[{\hat{\rho }}^{[n,m]}\,({\hat{d}}_{{l}_{n}j}^{[n]}\otimes {\hat{d}}_{{l}_{m}k}^{[m]})] & = & 0\\ \Rightarrow  & 4\,\sum _{n}\,{\rm{I}}{\rm{m}}\,{\rm{T}}{\rm{r}}\,[\sum _{{l}_{n}}\,{\hat{\rho }}^{[n]}{\hat{d}}_{{l}_{n}j}^{[n]}{\hat{d}}_{{l}_{n}k}^{[n]}] & = & 0,\end{array}$$

since ${\sum }_{{l}_{n},{l}_{m}}\,{\rm{Tr}}\,[{\hat{\rho }}^{[n,m]}\,({\hat{d}}_{{l}_{n}j}^{[n]}\otimes {\hat{d}}_{{l}_{m}k}^{[m]})]\in {\mathbb{R}}$. Hence, the attainability of the quantum enhancement to the estimation precision is determined solely by the one-particle reduced density matrices of the probe state.

Consider the magnetic field example again here in the context of noisy channel. The same permutationally invariant mixed input probe state is used. Thus, the first and second order marginals are the same. Moreover, for the purposes of this example here, each Pauli operator ${\hat{\sigma }}_{k}$ for *k* = 1, 2, 3 (corresponding to *X*, *Y* and *Z* directions) can be split into a sum of two single particle Kraus operators as ${\hat{\sigma }}_{k}={\sum }_{l=1}^{2}\,{\hat{\pi }}_{lk}$, so that ${\hat{\pi }}_{l}={\sum }_{k=1}^{3}\,{\theta }_{k}{\hat{\pi }}_{lk}$, e.g.

47 $$\begin{array}{ccc}{\hat{\sigma }}_{1} & = & [\begin{array}{cc}0 & 1\\ 1 & 0\end{array}]=[\begin{array}{cc}0 & 1\\ 0 & 0\end{array}]+[\begin{array}{cc}0 & 0\\ 1 & 0\end{array}],\\ {\hat{\sigma }}_{2} & = & [\begin{array}{cc}0 & -i\\ i & 0\end{array}]=[\begin{array}{cc}0 & -i\\ 0 & 0\end{array}]+[\begin{array}{cc}0 & 0\\ i & 0\end{array}],\\ {\hat{\sigma }}_{3} & = & [\begin{array}{cc}1 & 0\\ 0 & -1\end{array}]=[\begin{array}{cc}1 & 0\\ 0 & 0\end{array}]+[\begin{array}{cc}0 & 0\\ 0 & -1\end{array}].\end{array}$$

One can verify that such a decomposition for each Pauli operator ${\hat{\sigma }}_{k}$ satisfies ${\sum }_{l}\,{\hat{\pi }}_{lk}^{\dagger }{\hat{\pi }}_{lk}={\sum }_{l}\,{\hat{\pi }}_{lk}{\hat{\pi }}_{lk}^{\dagger }={1}_{2}$. Then,

48 $${\hat{d}}_{lk}=\frac{1}{2}\,{\int }_{0}^{1}\,d\alpha {e}^{i\alpha {\hat{\pi }}_{l}}{\hat{\pi }}_{lk}{e}^{-i\alpha {\hat{\pi }}_{l}}.$$

Then, we get:

49 $${C}_{Q}^{jk,[1]}=\sum _{l,p}\,{\rm{R}}{\rm{e}}\,[2\,{\rm{T}}{\rm{r}}\,[{\hat{d}}_{lj}{\hat{d}}_{lk}]-{\rm{T}}{\rm{r}}\,[{\hat{d}}_{lj}]\,{\rm{T}}{\rm{r}}\,[{\hat{d}}_{pk}]],$$

and

50 $$\begin{array}{ccc}{C}_{Q}^{jk,[2]} & = & \frac{1}{3}\,\sum _{l,p}\,\sum _{t=1}^{3}\,{\rm{R}}{\rm{e}}\,[{\rm{T}}{\rm{r}}\,[(\sum _{r=0}^{1}\,{\hat{E}}_{r}{\hat{\sigma }}_{t}{\hat{E}}_{r}\otimes \sum _{s=0}^{1}\,{\hat{E}}_{s}{\hat{\sigma }}_{t}{\hat{E}}_{s})\,({\hat{d}}_{lj}\otimes {\hat{d}}_{pk})]]\\  & = & \frac{2}{3}\,\sum _{l,p}\,{\rm{R}}{\rm{e}}\,[{\rm{T}}{\rm{r}}\,[(\sum _{r=0}^{1}\,{\hat{E}}_{r}{\hat{d}}_{lj}{\hat{E}}_{r})\,(\sum _{s=0}^{1}\,{\hat{E}}_{s}{\hat{d}}_{pk}{\hat{E}}_{s})]].\end{array}$$

Define $${\hat{g}}_{lj}={\sum }_{r=0}^{1}\,{\hat{E}}_{r}{\hat{d}}_{lj}{\hat{E}}_{r}$$ and $${\hat{g}}_{pk}={\sum }_{s=0}^{1}\,{\hat{E}}_{s}{\hat{d}}_{pk}{\hat{E}}_{s}$$.

Thus, from (44), (49) and (50), we get:

$$\begin{array}{ccc}{C}_{Q}^{jk} & = & N\,\sum _{l,p}\,{\rm{R}}{\rm{e}}\,[2\,{\rm{T}}{\rm{r}}\,[{\hat{d}}_{lj}{\hat{d}}_{lk}]-{\rm{T}}{\rm{r}}\,[{\hat{d}}_{lj}]\,{\rm{T}}{\rm{r}}\,[{\hat{d}}_{pk}]]\,+\,\frac{2N(N-1)}{3}\,\sum _{l,p}\,{\rm{R}}{\rm{e}}\,[{\rm{T}}{\rm{r}}\,[{\hat{g}}_{lj}{\hat{g}}_{pk}]],\end{array}$$

where all the quantities may be explicitly calculated.

Again, note that when the terms ${\rm{Tr}}\,[{\hat{d}}_{lj}{\hat{d}}_{pk}]$ are all zero, the terms ${\rm{Tr}}\,[{\hat{g}}_{lj}{\hat{g}}_{pk}]$ in general (i.e. when ${\hat{E}}_{0}$ and ${\hat{E}}_{1}$ need not be local dephasing operators) can be non-zero, such that it is possible to achieve the Heisenberg limit with the presence of noise in the initial probe state, even when it cannot be achieved in the absence of noise in the initial probe state. Moreover, when the terms ${\rm{Tr}}\,[{\hat{d}}_{lj}{\hat{d}}_{pk}]$ are not all zero, the terms ${\rm{Tr}}\,[{\hat{g}}_{lj}{\hat{g}}_{pk}]$ can be such that *C*_*Q*_ with noise in the initial probe state, such as by means of ${\hat{E}}_{0}$ and ${\hat{E}}_{1}$ for local dissipation, is larger than that without noise in the initial probe state, so that the estimation precision can be better with noise in the initial probe state than that without noise in the initial state.

We next consider the more general situation, where the noisy channel need not be necessarily unital, and illustrate that the presence of noise in the channel can actually serve as a feature rather than a bug, since even when the Heisenberg precision scaling cannot be achieved with a unitary channel, it is possible to attain the Heisenberg scaling, and in fact, even beat it with a noisy channel.

## Noise in Channel as a Feature rather than a Bug

We now look at the utility of the presence of noise in a general channel in achieving or even beating the Heisenberg precision limit.

Consider first the case of a mixed probe state, comprising *N* particles, evolving through a unitary channel, and that the *N* particles of the probe undergo *N* independent ***θ***-dependent unitary evolutions, i.e. the unitary operator of the channel is a product of *N* independent unitary operators $\hat{U}({\boldsymbol{\theta }})={\otimes }_{n=1}^{N}\,{\hat{U}}_{(n)}({\boldsymbol{\theta }})$.

Then, the QFIM takes the form as in (30) as follows:

$$\begin{array}{ccc}{J}_{Q}^{jk} & = & 4{\rm{R}}{\rm{e}}\,\sum _{n}\,[{\rm{T}}{\rm{r}}\,(\frac{{\rm{\partial }}{\hat{U}}_{(n)}^{\dagger }({\boldsymbol{\theta }})}{{\rm{\partial }}{\theta }_{j}}\frac{{\rm{\partial }}{\hat{U}}_{(n)}({\boldsymbol{\theta }})}{{\rm{\partial }}{\theta }_{k}}{\hat{\rho }}^{[n]})\,-\,{\rm{T}}{\rm{r}}\,(i\frac{{\rm{\partial }}{\hat{U}}_{(n)}^{\dagger }({\boldsymbol{\theta }})}{{\rm{\partial }}{\theta }_{j}}{\hat{U}}_{(n)}({\boldsymbol{\theta }}){\hat{\rho }}^{[n]})\,{\rm{T}}{\rm{r}}\,(i\frac{{\rm{\partial }}{\hat{U}}_{(n)}^{\dagger }({\boldsymbol{\theta }})}{{\rm{\partial }}{\theta }_{k}}{\hat{U}}_{(n)}({\boldsymbol{\theta }}){\hat{\rho }}^{[n]})]\\  &  & +\,4{\rm{R}}{\rm{e}}\,\sum _{n\ne m}\,[{\rm{T}}{\rm{r}}\,\{(\frac{{\rm{\partial }}{\hat{U}}_{(n)}^{\dagger }({\boldsymbol{\theta }})}{{\rm{\partial }}{\theta }_{j}}{\hat{U}}_{(n)}({\boldsymbol{\theta }})\otimes {\hat{U}}_{(m)}^{\dagger }({\boldsymbol{\theta }})\frac{{\rm{\partial }}{\hat{U}}_{(m)}({\boldsymbol{\theta }})}{{\rm{\partial }}{\theta }_{k}})\,{\hat{\rho }}^{[n,m]}\}\,\,-\,{\rm{T}}{\rm{r}}\,(i\frac{{\rm{\partial }}{\hat{U}}_{(n)}^{\dagger }({\boldsymbol{\theta }})}{{\rm{\partial }}{\theta }_{j}}{\hat{U}}_{(n)}({\boldsymbol{\theta }}){\hat{\rho }}^{[n]})\,{\rm{T}}{\rm{r}}\,(i\frac{{\rm{\partial }}{\hat{U}}_{(m)}^{\dagger }({\boldsymbol{\theta }})}{{\rm{\partial }}{\theta }_{k}}{\hat{U}}_{(m)}({\boldsymbol{\theta }}){\hat{\rho }}^{[m]})]\\  & = & {J}_{Q}^{jk,[n]}+{J}_{Q}^{jk,[n,m]}.\end{array}$$

Now, note that the first term ${J}_{Q}^{jk,[n]}$ is of O(*N*) and the second term ${J}_{Q}^{jk,[n,m]}$ is of O(*N*^2^), as they involve *N* and *N*(*N* − 1)/2 terms, respectively. Then, the term ${J}_{Q}^{jk,[n,m]}$ should be non-zero, implying that quantum correlations amongst the particles play a role in attaining the Heisenberg scaling of 1/*N*. As observed earlier, if the probe state is a product state, i.e. $\hat{\rho }={\otimes }_{n=1}^{N}\,{\hat{\rho }}^{[n]}$, then we have ${\hat{\rho }}^{[n,m]}={\hat{\rho }}^{[n]}\otimes {\hat{\rho }}^{[m]}$, and consequently ${J}_{Q}^{jk,[n,m]}=0$, such that the Heisenberg scaling is lost and the covariance scales as $1/\sqrt{N}$ at best. Also, if both ${\hat{\rho }}^{[n]}$ and ${\hat{\rho }}^{[n,m]}$ are maximally mixed, the Heisenberg scaling is lost again and the best scaling for the covariance is $1/\sqrt{N}$, implying that too much quantum correlations harms the quantum advantage with *N* parallel resources. Classical correlations in the initial probe state cannot be converted into quantum correlations by a unitary channel and cannot allow for an advantage over the scaling $1/\sqrt{N}$. Thus, any quantum enhancement to the estimation precision is provided by the two-particle reduced density matrices of the probe state. Notice that the saturability condition (8) here yields:

51 $$\begin{array}{cc} & 4{\rm{I}}{\rm{m}}\,\sum _{n}\,{\rm{T}}{\rm{r}}\,({\textstyle \tfrac{{\rm{\partial }}{\hat{U}}_{(n)}^{\dagger }({\boldsymbol{\theta }})}{{\rm{\partial }}{\theta }_{j}}}{\textstyle \tfrac{{\rm{\partial }}{\hat{U}}_{(n)}({\boldsymbol{\theta }})}{{\rm{\partial }}{\theta }_{k}}}{\hat{\rho }}^{[n]})\\  & +4{\rm{I}}{\rm{m}}\,\sum _{n\ne m}\,{\rm{T}}{\rm{r}}\,\{({\textstyle \tfrac{{\rm{\partial }}{\hat{U}}_{(n)}^{\dagger }({\boldsymbol{\theta }})}{{\rm{\partial }}{\theta }_{j}}}{\hat{U}}_{(n)}({\boldsymbol{\theta }})\otimes {\hat{U}}_{(m)}^{\dagger }({\boldsymbol{\theta }}){\textstyle \tfrac{{\rm{\partial }}{\hat{U}}_{(m)}({\boldsymbol{\theta }})}{{\rm{\partial }}{\theta }_{k}}}){\hat{\rho }}^{[n,m]}\}=0\\ \Rightarrow  & 4{\rm{I}}{\rm{m}}\,\sum _{n}\,{\rm{T}}{\rm{r}}\,({\textstyle \tfrac{{\rm{\partial }}{\hat{U}}_{(n)}^{\dagger }({\boldsymbol{\theta }})}{{\rm{\partial }}{\theta }_{j}}}{\textstyle \tfrac{{\rm{\partial }}{\hat{U}}_{(n)}({\boldsymbol{\theta }})}{{\rm{\partial }}{\theta }_{k}}}{\hat{\rho }}^{[n]})=0,\end{array}$$

since ${\hat{U}}_{(n/m)}^{\dagger }({\boldsymbol{\theta }}){\hat{U}}_{(n/m)}({\boldsymbol{\theta }})={1}_{2}\,{\rm{\forall }}n,m$. Clearly, the attainability of the quantum enhancement to the estimation precision is determined solely by the one-particle reduced density matrices of the initial mixed probe state.

Next, consider the case of a mixed initial probe state, comprising *N* particles, evolving through a noisy quantum channel, and that the *N* particles of the initial probe state undergo *N* independent ***θ***-dependent evolutions, i.e. the Kraus operator of the noisy quantum channel is a product of *N* independent Kraus operators ${\hat{{\rm{\Pi }}}}_{l}({\boldsymbol{\theta }})={\otimes }_{n=1}^{N}\,{\hat{{\rm{\Pi }}}}_{{l}_{n}}^{(n)}({\boldsymbol{\theta }})$, where we have *l* = (*l*_1_, *l*_2_, …, *l*_*N*_).

Then, (32) takes the form:

$$\begin{array}{ccc}{C}_{Q}^{jk} & = & 4{\rm{R}}{\rm{e}}\,\sum _{n}\,[{\rm{T}}{\rm{r}}\,(\sum _{{l}_{n}}{\textstyle \tfrac{{\rm{\partial }}{\hat{{\rm{\Pi }}}}_{{l}_{n}}^{(n)\dagger }({\boldsymbol{\theta }})}{{\rm{\partial }}{\theta }_{j}}}{\textstyle \tfrac{{\rm{\partial }}{\hat{{\rm{\Pi }}}}_{{l}_{n}}^{(n)}({\boldsymbol{\theta }})}{{\rm{\partial }}{\theta }_{k}}}{\hat{\rho }}^{[n]})\,-\,{\rm{T}}{\rm{r}}\,(i\,\sum _{{l}_{{n}_{1}}}\,{\textstyle \tfrac{{\rm{\partial }}{\hat{{\rm{\Pi }}}}_{{l}_{{n}_{1}}}^{(n)\dagger }({\boldsymbol{\theta }})}{{\rm{\partial }}{\theta }_{j}}}{\hat{{\rm{\Pi }}}}_{{l}_{{n}_{1}}}({\boldsymbol{\theta }}){\hat{\rho }}^{[n]})\,{\rm{T}}{\rm{r}}\,(i\,\sum _{{l}_{{n}_{2}}}\,{\textstyle \tfrac{{\rm{\partial }}{\hat{{\rm{\Pi }}}}_{{l}_{{n}_{2}}}^{(n)\dagger }({\boldsymbol{\theta }})}{{\rm{\partial }}{\theta }_{k}}}{\hat{{\rm{\Pi }}}}_{{l}_{{n}_{2}}}^{(n)}({\boldsymbol{\theta }}){\hat{\rho }}^{[n]})]\\  &  & +\,4{\rm{R}}{\rm{e}}\,\sum _{n\ne m}\,\sum _{{l}_{n},{l}_{m}}\,[{\rm{T}}{\rm{r}}\,\{({\textstyle \tfrac{{\rm{\partial }}{\hat{{\rm{\Pi }}}}_{{l}_{n}}^{(n)\dagger }({\boldsymbol{\theta }})}{{\rm{\partial }}{\theta }_{j}}}{\hat{{\rm{\Pi }}}}_{{l}_{n}}^{(n)}({\boldsymbol{\theta }})\otimes {\hat{{\rm{\Pi }}}}_{{l}_{m}}^{(m)\dagger }({\boldsymbol{\theta }}){\textstyle \tfrac{{\rm{\partial }}{\hat{{\rm{\Pi }}}}_{{l}_{m}}^{(m)}({\boldsymbol{\theta }})}{{\rm{\partial }}{\theta }_{k}}}){\hat{\rho }}^{[n,m]}\}\,\,-\,{\rm{T}}{\rm{r}}\,(i{\textstyle \tfrac{{\rm{\partial }}{\hat{{\rm{\Pi }}}}_{{l}_{n}}^{(n)\dagger }({\boldsymbol{\theta }})}{{\rm{\partial }}{\theta }_{j}}}{\hat{{\rm{\Pi }}}}_{{l}_{n}}^{(n)}({\boldsymbol{\theta }}){\hat{\rho }}^{[n]})\,{\rm{T}}{\rm{r}}\,(i{\textstyle \tfrac{{\rm{\partial }}{\hat{{\rm{\Pi }}}}_{{l}_{m}}^{(m)\dagger }({\boldsymbol{\theta }})}{{\rm{\partial }}{\theta }_{k}}}{\hat{{\rm{\Pi }}}}_{{l}_{m}}^{(m)}({\boldsymbol{\theta }}){\hat{\rho }}^{[m]})]\\  & = & {C}_{Q}^{jk,[n]}+{C}_{Q}^{jk,[n,m]}.\end{array}$$

Again, note that the first term ${C}_{Q}^{jk,[n]}$ is of O(*N*) and the second term ${C}_{Q}^{jk,[n,m]}$ is of O(*N*^2^), as they involve *N* and *N*(*N* − 1)/2 terms, respectively. Then, the term ${C}_{Q}^{jk,[n,m]}$ should be non-zero, implying that quantum correlations amongst the particles play a role in attaining the Heisenberg scaling of 1/*N* or better. Now, if the initial probe state is separable but not a product state, then that leads to ${C}_{Q}^{jk,[n,m]}\ne 0$. This is because, as noted earlier, although noise is widely known to reduce quantum correlations in a system in most cases^24,61^, noise can also introduce or increase quantum correlations in a system^62–65^, that may then be activated into entanglement^60,69^. Even without quantum correlations between the particles of the initial probe state, an estimation precision scaling of 1/*N* or better can be achieved, when the initial probe state has classical correlations, that can be converted into quantum correlations by non-unital noise in the channel, unlike in cases of mixed state evolving unitarily or unitally considered earlier. Thus, noise in the quantum channel can act as a feature rather than a bug, since we see that the estimation precision that can be achieved with a noisy channel in some situations is impossible with a noiseless unitary channel. However, if both ${\hat{\rho }}^{[n]}$ and ${\hat{\rho }}^{[n,m]}$ are maximally mixed, we get ${C}_{Q}^{jk,[n,m]}=0$, so a best precision scaling of $1/\sqrt{N}$ can be achieved.

Moreover, if there exists some Kraus representation ${\hat{{\rm{\Pi }}}}_{l}({\boldsymbol{\theta }})$ of the quantum channel which renders ${C}_{Q}^{jk,[n,m]}=0$, then the covariance scales as $1/\sqrt{N}$ at best, even when the particles of the initial probe state are entangled. Extending the argument from ref.^26^ to the multiparameter case, the covariance also scales as $1/\sqrt{N}$ at most, even in the presence of feedback control. Thus, any quantum enhancement to the estimation precision is provided by the two-particle reduced density matrices of the initial probe state.

Notice that the saturability condition (35) here becomes:

52 $$\begin{array}{cc} & 4{\rm{I}}{\rm{m}}\,\sum _{n}\,{\rm{T}}{\rm{r}}\,(\sum _{{l}_{n}}\,{\textstyle \tfrac{{\rm{\partial }}{\hat{{\rm{\Pi }}}}_{{l}_{n}}^{(n)\dagger }({\boldsymbol{\theta }})}{{\rm{\partial }}{\theta }_{j}}}{\textstyle \tfrac{{\rm{\partial }}{\hat{{\rm{\Pi }}}}_{{l}_{n}}^{(n)}({\boldsymbol{\theta }})}{{\rm{\partial }}{\theta }_{k}}}{\hat{\rho }}^{[n]})\\  & +4{\rm{I}}{\rm{m}}\,\sum _{n\ne m}\,\sum _{{l}_{n},{l}_{m}}\,{\rm{T}}{\rm{r}}\,\{({\textstyle \tfrac{{\rm{\partial }}{\hat{{\rm{\Pi }}}}_{{l}_{n}}^{(n)\dagger }({\boldsymbol{\theta }})}{{\rm{\partial }}{\theta }_{j}}}{\hat{{\rm{\Pi }}}}_{{l}_{n}}^{(n)}({\boldsymbol{\theta }})\otimes {\hat{{\rm{\Pi }}}}_{{l}_{m}}^{(m)\dagger }({\boldsymbol{\theta }}){\textstyle \tfrac{{\rm{\partial }}{\hat{{\rm{\Pi }}}}_{{l}_{m}}^{(m)}({\boldsymbol{\theta }})}{{\rm{\partial }}{\theta }_{k}}})\,{\hat{\rho }}^{[n,m]}\}=0\\ \Rightarrow  & 4{\rm{I}}{\rm{m}}\,\sum _{n}\,{\rm{T}}{\rm{r}}\,(\sum _{{l}_{n}}\,{\textstyle \tfrac{{\rm{\partial }}{\hat{{\rm{\Pi }}}}_{{l}_{n}}^{(n)\dagger }({\boldsymbol{\theta }})}{{\rm{\partial }}{\theta }_{j}}}{\textstyle \tfrac{{\rm{\partial }}{\hat{{\rm{\Pi }}}}_{{l}_{n}}^{(n)}({\boldsymbol{\theta }})}{{\rm{\partial }}{\theta }_{k}}}{\hat{\rho }}^{[n]})=0,\end{array}$$

since ${\sum }_{{l}_{n}/{l}_{m}}\,{\hat{{\rm{\Pi }}}}_{{l}_{n}/{l}_{m}}^{(n/m)\dagger }({\boldsymbol{\theta }}){\hat{{\rm{\Pi }}}}_{{l}_{n}/{l}_{m}}^{(n/m)}({\boldsymbol{\theta }})={1}_{2}\,{\rm{\forall }}n,m$. Clearly, the attainability of the quantum enhancement to the estimation precision is determined solely by the one-particle reduced density matrices of the probe state.

Now, in terms of the evolved probe state $\hat{\rho }({\boldsymbol{\theta }})$, (32) takes the following form. We get the below *C*_*Q*_ from *J*_*Q*_ defined in the *S* + *B* space by tracing out the bath *B*, and this is equivalent to *C*_*Q*_ in terms of the initial state.

$$\begin{array}{ccc}{C}_{Q}^{jk} & = & 4{\rm{R}}{\rm{e}}\,[{\rm{T}}{\rm{r}}\,(\sum _{l}\,{\hat{{\rm{\Pi }}}}_{l}({\boldsymbol{\theta }}){\textstyle \tfrac{{\rm{\partial }}{\hat{{\rm{\Pi }}}}_{l}^{\dagger }({\boldsymbol{\theta }})}{{\rm{\partial }}{\theta }_{j}}}{\textstyle \tfrac{{\rm{\partial }}{\hat{{\rm{\Pi }}}}_{l}({\boldsymbol{\theta }})}{{\rm{\partial }}{\theta }_{k}}}{\hat{{\rm{\Pi }}}}_{l}^{\dagger }({\boldsymbol{\theta }})\hat{\rho }({\boldsymbol{\theta }}))\,-\,{\rm{T}}{\rm{r}}\,(i\,\sum _{p}\,{\hat{{\rm{\Pi }}}}_{p}({\boldsymbol{\theta }}){\textstyle \tfrac{{\rm{\partial }}{\hat{{\rm{\Pi }}}}_{p}^{\dagger }({\boldsymbol{\theta }})}{{\rm{\partial }}{\theta }_{j}}}\hat{\rho }({\boldsymbol{\theta }}))\,{\rm{T}}{\rm{r}}\,(i\,\sum _{r}\,{\hat{{\rm{\Pi }}}}_{r}(\theta ){\textstyle \tfrac{{\rm{\partial }}{\hat{{\rm{\Pi }}}}_{r}^{\dagger }(\theta )}{{\rm{\partial }}{\theta }_{k}}}\hat{\rho }(\theta ))]\\  & = & 4{\rm{R}}{\rm{e}}\,\sum _{n}\,[{\rm{T}}{\rm{r}}\,(\sum _{{l}_{n}}\,{\hat{{\rm{\Pi }}}}_{{l}_{n}}^{(n)}({\boldsymbol{\theta }}){\textstyle \tfrac{{\rm{\partial }}{\hat{{\rm{\Pi }}}}_{{l}_{n}}^{(n)\dagger }({\boldsymbol{\theta }})}{{\rm{\partial }}{\theta }_{j}}}{\textstyle \tfrac{{\rm{\partial }}{\hat{{\rm{\Pi }}}}_{{l}_{n}}^{(n)}({\boldsymbol{\theta }})}{{\rm{\partial }}{\theta }_{k}}}{\hat{{\rm{\Pi }}}}_{{l}_{n}}^{(n)\dagger }({\boldsymbol{\theta }}){\hat{\rho }}^{[n]}({\boldsymbol{\theta }}))\,-\,{\rm{T}}{\rm{r}}\,(i\,\sum _{{l}_{{n}_{1}}}\,{\hat{{\rm{\Pi }}}}_{{l}_{{n}_{1}}}^{(n)}({\boldsymbol{\theta }}){\textstyle \tfrac{{\rm{\partial }}{\hat{{\rm{\Pi }}}}_{{l}_{{n}_{1}}}^{(n)\dagger }({\boldsymbol{\theta }})}{{\rm{\partial }}{\theta }_{j}}}{\hat{\rho }}^{[n]}({\boldsymbol{\theta }}))\,{\rm{T}}{\rm{r}}\,(i\,\sum _{{l}_{{n}_{2}}}\,{\hat{{\rm{\Pi }}}}_{{l}_{{n}_{2}}}^{(n)}({\boldsymbol{\theta }}){\textstyle \tfrac{{\rm{\partial }}{\hat{{\rm{\Pi }}}}_{{l}_{{n}_{2}}}^{(n)\dagger }({\boldsymbol{\theta }})}{{\rm{\partial }}{\theta }_{k}}}{\hat{\rho }}^{[n]}({\boldsymbol{\theta }}))]\\  &  & +\,4{\rm{R}}{\rm{e}}\,\sum _{n\ne m}\,\sum _{{l}_{n},{l}_{m}}\,[{\rm{T}}{\rm{r}}\,\{({\hat{{\rm{\Pi }}}}_{{l}_{n}}^{(n)}({\boldsymbol{\theta }}){\textstyle \tfrac{{\rm{\partial }}{\hat{{\rm{\Pi }}}}_{{l}_{n}}^{(n)\dagger }({\boldsymbol{\theta }})}{{\rm{\partial }}{\theta }_{j}}}\otimes {\textstyle \tfrac{{\rm{\partial }}{\hat{{\rm{\Pi }}}}_{{l}_{m}}^{(m)}({\boldsymbol{\theta }})}{{\rm{\partial }}{\theta }_{k}}}{\hat{{\rm{\Pi }}}}_{{l}_{m}}^{(m)\dagger }({\boldsymbol{\theta }})){\hat{\rho }}^{[n,m]}({\boldsymbol{\theta }})\}\,-\,{\rm{T}}{\rm{r}}\,(i{\hat{{\rm{\Pi }}}}_{{l}_{n}}^{(n)}({\boldsymbol{\theta }}){\textstyle \tfrac{{\rm{\partial }}{\hat{{\rm{\Pi }}}}_{{l}_{n}}^{(n)\dagger }({\boldsymbol{\theta }})}{{\rm{\partial }}{\theta }_{j}}}{\hat{\rho }}^{[n]}({\boldsymbol{\theta }}))\,{\rm{T}}{\rm{r}}\,(i{\hat{{\rm{\Pi }}}}_{{l}_{m}}^{(m)}({\boldsymbol{\theta }}){\textstyle \tfrac{{\rm{\partial }}{\hat{{\rm{\Pi }}}}_{{l}_{m}}^{(m)\dagger }({\boldsymbol{\theta }})}{{\rm{\partial }}{\theta }_{k}}}{\hat{\rho }}^{[m]}({\boldsymbol{\theta }}))]\\  & = & {C}_{Q}^{jk,[n]}+{C}_{Q}^{jk,[n,m]}.\end{array}$$

Clearly, if the final probe state is a product state, we get ${C}_{Q}^{jk,[n,m]}=0$, such that a scaling of $1/\sqrt{N}$ can be attained at best. This implies that noise in the channel should introduce quantum correlations between the particles of the probe state, in order to provide quantum advantage in achieving an estimation precision scaling of 1/*N* or better. Also, if both ${\hat{\rho }}^{[n]}({\boldsymbol{\theta }})$ and ${\hat{\rho }}^{[n,m]}({\boldsymbol{\theta }})$ are maximally mixed, we get ${C}_{Q}^{jk,[n,m]}=0$. This implies that a lot of noise in the channel can introduce too much quantum correlations between the particles of the probe state, such that a best precision scaling of $1/\sqrt{N}$ can be achieved. Thus, some amount of noise in the quantum channel can act as a feature rather than a bug by introducing quantum correlations into the system, but excessive noise destroys the achievable quantum advantage with *N* parallel resources.

## Beating the Heisenberg Limit

We show in Section X of Supplementary Information that unless the following condition is also satisfied by the channel Kraus operators:

53 $$\begin{array}{cccc} & \sum _{l}\,\frac{{\rm{\partial }}{\hat{{\rm{\Pi }}}}_{l}({\boldsymbol{\theta }})}{{\rm{\partial }}{\theta }_{k}}\hat{\rho }{\hat{{\rm{\Pi }}}}_{l}^{\dagger }({\boldsymbol{\theta }}) & = & \sum _{l}\,\frac{{\rm{\partial }}{\hat{{\rm{\Pi }}}}_{l}({\boldsymbol{\theta }})}{{\rm{\partial }}{\theta }_{k}}\hat{\rho }\\ \Rightarrow  & \sum _{l}\,{\rm{T}}{\rm{r}}\,[\frac{{\rm{\partial }}{\hat{{\rm{\Pi }}}}_{l}^{\dagger }({\boldsymbol{\theta }})}{{\rm{\partial }}{\theta }_{j}}{\hat{{\rm{\Pi }}}}_{l}({\boldsymbol{\theta }}){\hat{{\rm{\Pi }}}}_{l}^{\dagger }({\boldsymbol{\theta }})\frac{{\rm{\partial }}{\hat{{\rm{\Pi }}}}_{l}({\boldsymbol{\theta }})}{{\rm{\partial }}{\theta }_{k}}\hat{\rho }] & = & \sum _{l}\,{\rm{T}}{\rm{r}}\,[\frac{{\rm{\partial }}{\hat{{\rm{\Pi }}}}_{l}^{\dagger }({\boldsymbol{\theta }})}{{\rm{\partial }}{\theta }_{j}}\frac{{\rm{\partial }}{\hat{{\rm{\Pi }}}}_{l}({\boldsymbol{\theta }})}{{\rm{\partial }}{\theta }_{k}}\hat{\rho }]\\ \Rightarrow  & \sum _{l}\,{\hat{{\rm{\Pi }}}}_{l}({\boldsymbol{\theta }}){\hat{{\rm{\Pi }}}}_{l}^{\dagger }({\boldsymbol{\theta }}) & = & 1,\end{array}$$

i.e. unless the channel is unital, any noise in the channel may beat the Heisenberg limit, when (35) is satisfied. An outline of the proof is given in the Methods section.

However, since the Heisenberg limit is not ultimate, e.g. see refs^70–83^, although this has sparked some controversy^84–90^, now the question is what is the fundamental ultimate quantum limit to the achievable estimation precision in the presence of optimal amount of noise in a non-unitary quantum channel. In other words, what should the quantity *C*_*Q*_ look like when the precision achievable is maximum in a non-unitary channel. It is fairly easy to see that for optimal quantity of noise in the channel, the two-particle reduced density operators of the evolved probe state should be a maximally entangled mixed state (MEMS) and the one-particle reduced density operators of the evolved probe state should be a maximally mixed state^91–93^. Therefore, we must have the reduced density operators of the evolved probe state as: ${\hat{\rho }}^{[n]}({\boldsymbol{\theta }})={1}_{2}/2$ and ${\hat{\rho }}_{MEMS}^{[n,m]}({\boldsymbol{\theta }})\ne {\hat{\rho }}^{[n]}({\boldsymbol{\theta }})\otimes {\hat{\rho }}^{[m]}({\boldsymbol{\theta }})$.

Then, the fundamental quantum limit to the achievable estimation precision in a noisy channel is given by the following:

$$\begin{array}{ccc}{J}_{SH}^{jk} & = & {\rm{R}}{\rm{e}}\,\sum _{n}\,[2{\rm{T}}{\rm{r}}\,(\sum _{{l}_{n}}\,{\hat{{\rm{\Pi }}}}_{{l}_{n}}^{(n)}({\boldsymbol{\theta }}){\textstyle \tfrac{{\rm{\partial }}{\hat{{\rm{\Pi }}}}_{{l}_{n}}^{(n)\dagger }({\boldsymbol{\theta }})}{{\rm{\partial }}{\theta }_{j}}}{\textstyle \tfrac{{\rm{\partial }}{\hat{{\rm{\Pi }}}}_{{l}_{n}}^{(n)}({\boldsymbol{\theta }})}{{\rm{\partial }}{\theta }_{k}}}{\hat{{\rm{\Pi }}}}_{{l}_{n}}^{(n)\dagger }({\boldsymbol{\theta }}))\,-\,{\rm{T}}{\rm{r}}\,(i\,\sum _{{l}_{{n}_{1}}}\,{\hat{{\rm{\Pi }}}}_{{l}_{{n}_{1}}}^{(n)}({\boldsymbol{\theta }}){\textstyle \tfrac{{\rm{\partial }}{\hat{{\rm{\Pi }}}}_{{l}_{{n}_{1}}}^{(n)\dagger }({\boldsymbol{\theta }})}{{\rm{\partial }}{\theta }_{j}}})\,{\rm{T}}{\rm{r}}\,(i\,\sum _{{l}_{{n}_{2}}}\,{\hat{{\rm{\Pi }}}}_{{l}_{{n}_{2}}}^{(n)}({\boldsymbol{\theta }}){\textstyle \tfrac{{\rm{\partial }}{\hat{{\rm{\Pi }}}}_{{l}_{{n}_{2}}}^{(n)\dagger }({\boldsymbol{\theta }})}{{\rm{\partial }}{\theta }_{k}}})]\\  &  & +\,{\rm{R}}{\rm{e}}\,\sum _{n\ne m}\,\sum _{{l}_{n},{l}_{m}}\,[4{\rm{T}}{\rm{r}}\,\{({\hat{{\rm{\Pi }}}}_{{l}_{n}}^{(n)}({\boldsymbol{\theta }}){\textstyle \tfrac{{\rm{\partial }}{\hat{{\rm{\Pi }}}}_{{l}_{n}}^{(n)\dagger }({\boldsymbol{\theta }})}{{\rm{\partial }}{\theta }_{j}}}\otimes {\textstyle \tfrac{{\rm{\partial }}{\hat{{\rm{\Pi }}}}_{{l}_{m}}^{(m)}({\boldsymbol{\theta }})}{{\rm{\partial }}{\theta }_{k}}}{\hat{{\rm{\Pi }}}}_{{l}_{m}}^{(m)\dagger }({\boldsymbol{\theta }})){\hat{\rho }}_{MEMS}^{[n,m]}({\boldsymbol{\theta }})\}\,-\,{\rm{T}}{\rm{r}}\,(i{\hat{{\rm{\Pi }}}}_{{l}_{n}}^{(n)}({\boldsymbol{\theta }}){\textstyle \tfrac{{\rm{\partial }}{\hat{{\rm{\Pi }}}}_{{l}_{n}}^{(n)\dagger }({\boldsymbol{\theta }})}{{\rm{\partial }}{\theta }_{j}}})\,{\rm{T}}{\rm{r}}\,(i{\hat{{\rm{\Pi }}}}_{{l}_{m}}^{(m)}({\boldsymbol{\theta }}){\textstyle \tfrac{{\rm{\partial }}{\hat{{\rm{\Pi }}}}_{{l}_{m}}^{(m)\dagger }({\boldsymbol{\theta }})}{{\rm{\partial }}{\theta }_{k}}})]\\  & = & {J}_{SH}^{jk,[n]}+{J}_{SH}^{jk,[n,m]},\end{array}$$

where we have used the subscript “*SH*” to denote ‘super-Heisenberg’^80^ fundamental quantum estimation precision limit. The set of POVMs from (36) then saturates this ultimate limit. Note that a maximally discordant mixed state (MDMS) need not be maximally entangled^94^. In fact, it can be not entangled at all, but then it can be at best as nonclassical as (and not more nonclassical than) a maximally entangled pure state^60^, and therefore, cannot allow to beat the Heisenberg limit.

Note, however, that in order for entanglement to be activated from the quantum correlations in the probe state, multi-particle unitary maps (such as CNOT gates) are required^60,69^, if there was no entanglement in the initial probe state already or any entanglement in the initial probe state vanishes even if leaving the probe state maximally discordant. The Kraus representation of the channel is non-unique and is invariant under arbitrary unitary maps and so the above equations are invariant under addition of such unitary maps. But unless the quantum correlations are activated into entanglement, the above best estimation precision cannot be achieved. Thus, the active ancilla-assisted scheme from ref.^31^ can be strictly better than the passive ancilla-assisted scheme, since mixed entangled states can be more nonclassical than mixed separable states^60^. Note that a unitary operator is also a Kraus operator, and the identity operator is trivially unitary.

Now, without the additional unitary maps, that can activate entanglement from quantum correlations in the probe state, the best estimation precision limit is determined by the two-particle reduced density matrices of the evolved probe state being separable and maximally discordant (MDMS)^94^, i.e. the two-particle reduced density matrices having maximal dissonance^95^. Therefore, we must have ${\hat{\rho }}_{MDMS}^{[n,m]}({\boldsymbol{\theta }})\ne {\hat{\rho }}^{[n]}({\boldsymbol{\theta }})\otimes {\hat{\rho }}^{[m]}({\boldsymbol{\theta }})$, where we used the subscript “MDMS” to indicate unentangled and maximally discordant mixed state:

$$\begin{array}{ccc}{J}_{Q}^{jk} & = & 4{\rm{R}}{\rm{e}}\,\sum _{n}\,[{\rm{T}}{\rm{r}}\,(\sum _{{l}_{n}}\,{\hat{{\rm{\Pi }}}}_{{l}_{n}}^{(n)}({\boldsymbol{\theta }}){\textstyle \tfrac{{\rm{\partial }}{\hat{{\rm{\Pi }}}}_{{l}_{n}}^{(n)\dagger }({\boldsymbol{\theta }})}{{\rm{\partial }}{\theta }_{j}}}{\textstyle \tfrac{{\rm{\partial }}{\hat{{\rm{\Pi }}}}_{{l}_{n}}^{(n)}({\boldsymbol{\theta }})}{{\rm{\partial }}{\theta }_{k}}}{\hat{{\rm{\Pi }}}}_{{l}_{n}}^{(n)\dagger }({\boldsymbol{\theta }}){\hat{\rho }}^{[n]}({\boldsymbol{\theta }}))\,-\,{\rm{T}}{\rm{r}}\,(i\,\sum _{{l}_{{n}_{1}}}\,{\hat{{\rm{\Pi }}}}_{{l}_{{n}_{1}}}^{(n)}({\boldsymbol{\theta }}){\textstyle \tfrac{{\rm{\partial }}{\hat{{\rm{\Pi }}}}_{{l}_{{n}_{1}}}^{(n)\dagger }({\boldsymbol{\theta }})}{{\rm{\partial }}{\theta }_{j}}}{\hat{\rho }}^{[n]}({\boldsymbol{\theta }}))\,{\rm{T}}{\rm{r}}\,(i\,\sum _{{l}_{{n}_{2}}}\,{\hat{{\rm{\Pi }}}}_{{l}_{{n}_{2}}}^{(n)}({\boldsymbol{\theta }}){\textstyle \tfrac{{\rm{\partial }}{\hat{{\rm{\Pi }}}}_{{l}_{{n}_{2}}}^{(n)\dagger }({\boldsymbol{\theta }})}{{\rm{\partial }}{\theta }_{k}}}{\hat{\rho }}^{[n]}({\boldsymbol{\theta }}))]\\  &  & +\,4{\rm{R}}{\rm{e}}\,\sum _{n\ne m}\,\sum _{{l}_{n},{l}_{m}}\,[{\rm{T}}{\rm{r}}\,\{({\hat{{\rm{\Pi }}}}_{{l}_{n}}^{(n)}({\boldsymbol{\theta }}){\textstyle \tfrac{{\rm{\partial }}{\hat{{\rm{\Pi }}}}_{{l}_{n}}^{(n)\dagger }({\boldsymbol{\theta }})}{{\rm{\partial }}{\theta }_{j}}}\otimes {\textstyle \tfrac{{\rm{\partial }}{\hat{{\rm{\Pi }}}}_{{l}_{m}}^{(m)}({\boldsymbol{\theta }})}{{\rm{\partial }}{\theta }_{k}}}{\hat{{\rm{\Pi }}}}_{{l}_{m}}^{(m)\dagger }({\boldsymbol{\theta }})){\hat{\rho }}_{MDMS}^{[n,m]}({\boldsymbol{\theta }})\}\,-\,{\rm{T}}{\rm{r}}\,(i{\hat{{\rm{\Pi }}}}_{{l}_{n}}^{(n)}({\boldsymbol{\theta }}){\textstyle \tfrac{{\rm{\partial }}{\hat{{\rm{\Pi }}}}_{{l}_{n}}^{(n)\dagger }({\boldsymbol{\theta }})}{{\rm{\partial }}{\theta }_{j}}}{\hat{\rho }}^{[n]}({\boldsymbol{\theta }}))\,{\rm{T}}{\rm{r}}\,(i{\hat{{\rm{\Pi }}}}_{{l}_{m}}^{(m)}({\boldsymbol{\theta }}){\textstyle \tfrac{{\rm{\partial }}{\hat{{\rm{\Pi }}}}_{{l}_{m}}^{(m)\dagger }({\boldsymbol{\theta }})}{{\rm{\partial }}{\theta }_{k}}}{\hat{\rho }}^{[m]}({\boldsymbol{\theta }}))]\\  & = & {J}_{Q}^{jk,[n]}+{J}_{Q}^{jk,[n,m]},\end{array}$$

which corresponds to a precision scaling of 1/*N* for maximal pairwise quantum correlations, without entanglement, amongst the final probe particles^69^, since mixed separable states can be as nonclassical as entangled pure states^60^. Since the best estimation precision achievable with quantum correlations without entanglement coincides with and does not beat the Heisenberg limit, we used the subscript “*Q*” above.

Next, with the additional unitary maps and entanglement activated from the quantum correlations in the probe state, since the super-Heisenberg limit is obtained for the two-particle reduced density operators of the evolved probe state being maximally entangled and the one-particle reduced density operators being maximally mixed, the super-Heisenberg limit corresponds to a precision scaling of 1/*N*^2^ for maximal pairwise quantum correlations including entanglement amongst the final probe particles^74^. This is because mixed entangled bipartite states can be twice as nonclassical as maximally entangled bipartite pure states^60^.

Note that the precision scaling that could be achieved, e.g. in ref. ^74^, using two-particle Hamiltonians for a unitary channel, is achieved using one-particle Kraus operators for a noisy channel here, i.e. local noise inducing quantum correlations including entanglement amongst the two particles^64,65^. Notice that we did not get precision scaling better than 1/*N* when we studied the unitary channel case in this paper, since we considered only one-particle Hamiltonians. If we further considered *γ*-particle (instead of one-particle) Kraus operators for the noisy channel case here, with *γ* > 1, each set of Kraus operators can generate quantum correlations including entanglement induced by a common bath amongst the *γ* particles^62,63^. Then, the best super-Heisenberg precision scaling of 1/*N*^2*γ*^ may be attained, that is known to be only attainable using 2*γ*-particle Hamiltonians for a unitary channel. For example, using three-particle Kraus operators for a noisy channel, the best precision scaling of 1/*N*^6^ can be achieved, that is otherwise known to be possible with six-particle Hamiltonians for a unitary channel. This is again because mixed entangled states can be twice as nonclassical as pure entangled states^60^.

Considering again one-particle Kraus operators for the noisy channel, although the quantum Cramér-Rao bound (QCRB) can be beaten in the system space, the QCRB for the enlarged system plus bath space, for which the evolution is unitary, is not beaten. This also holds for multi-particle Kraus operators for the channel, where entanglement is induced by common baths. This implies that the estimation in the system space alone is not unbiased, when the QCRB, and therefore, the Heisenberg limit are beaten^23,85^. However, when the estimation involving measurements beats the QCRB, and therefore, the Heisenberg limit, it does not violate Robertson’s generalized formulation of Heisenberg’s uncertainty relation^23,24,96^, that does not include the measurement process. Note that the QCRB can be derived from the general Heisenberg’s uncertainty relation, upon considering that the estimator is unbiased^23^. Thus, beating the QCRB implies that the estimator bias is no longer zero (also see Section I of Supplementary Information), but does not violate the general Heisenberg’s uncertainty principle. Nonetheless, without including measurements, it is noteworthy that entanglement amongst the particles of a state allows for lower bounds for the dispersions of non-commuting observables than that furnished by the traditional Heisenberg’s uncertainty relation, originally derived for one particle^97^.

Finally, note that the super-Heisenberg limit will not necessarily be strictly less than the Heisenberg limit, such as when there are quantum correlations without entanglement in the evolved probe state. Moreover, if the two-particle reduced density matrices of the initial probe state are already maximally entangled, the super-Heisenberg limit will equal the Heisenberg limit. This is because it is only entanglement generated in the channel, i.e. in the evolution stage, that can contribute to a precision scaling better than the Heisenberg limit, and entanglement in the preparation and measurement stages are inessential^72^. Furthermore, the Heisenberg limit is not beaten, when the Kraus operators of the channel satisfy the condition (53). When the QCRB and the Heisenberg limit are not beaten, the estimator in the *S* space alone will be unbiased. Otherwise, when they are beaten, the estimator in the *S* space alone will be biased and may be of limited interest in practice.

The upper bound (32) to the QFIM reduces to the following actual QFIM, when (53) is satisfied:

$$\begin{array}{ccc}{J}_{Q}^{jk} & = & 4{\rm{R}}{\rm{e}}\,\sum _{n}\,[{\rm{T}}{\rm{r}}\,(\sum _{{l}_{n}}\,{\textstyle \tfrac{{\rm{\partial }}{\hat{{\rm{\Pi }}}}_{{l}_{n}}^{(n)\dagger }({\boldsymbol{\theta }})}{{\rm{\partial }}{\theta }_{j}}}{\textstyle \tfrac{{\rm{\partial }}{\hat{{\rm{\Pi }}}}_{{l}_{n}}^{(n)}({\boldsymbol{\theta }})}{{\rm{\partial }}{\theta }_{k}}}{\hat{\rho }}^{[n]})\,-\,{\rm{T}}{\rm{r}}\,(\sum _{{l}_{{n}_{1}}}\,{\textstyle \tfrac{{\rm{\partial }}{\hat{{\rm{\Pi }}}}_{{l}_{{n}_{1}}}^{(n)\dagger }({\boldsymbol{\theta }})}{{\rm{\partial }}{\theta }_{j}}}{\hat{{\rm{\Pi }}}}_{{l}_{{n}_{1}}}^{(n)}({\boldsymbol{\theta }}){\hat{\rho }}^{[n]})\,{\rm{T}}{\rm{r}}\,(\sum _{{l}_{{n}_{2}}}\,{\hat{{\rm{\Pi }}}}_{{l}_{{n}_{2}}}^{(n)\dagger }({\boldsymbol{\theta }}){\textstyle \tfrac{{\rm{\partial }}{\hat{{\rm{\Pi }}}}_{{l}_{{n}_{2}}}^{(n)}({\boldsymbol{\theta }})}{{\rm{\partial }}{\theta }_{k}}}{\hat{\rho }}^{[n]})]\\  &  & +\,4{\rm{R}}{\rm{e}}\,\sum _{n\ne m}\,\sum _{{l}_{n},{l}_{m}}\,[{\rm{T}}{\rm{r}}\,\{({\textstyle \tfrac{{\rm{\partial }}{\hat{{\rm{\Pi }}}}_{{l}_{n}}^{(n)\dagger }({\boldsymbol{\theta }})}{{\rm{\partial }}{\theta }_{j}}}{\hat{{\rm{\Pi }}}}_{{l}_{n}}^{(n)}({\boldsymbol{\theta }})\otimes {\hat{{\rm{\Pi }}}}_{{l}_{m}}^{(m)\dagger }({\boldsymbol{\theta }}){\textstyle \tfrac{{\rm{\partial }}{\hat{{\rm{\Pi }}}}_{{l}_{m}}^{(m)}({\boldsymbol{\theta }})}{{\rm{\partial }}{\theta }_{k}}}){\hat{\rho }}^{[n,m]}\}\,\,-\,{\rm{T}}{\rm{r}}\,({\textstyle \tfrac{{\rm{\partial }}{\hat{{\rm{\Pi }}}}_{{l}_{n}}^{(n)\dagger }({\boldsymbol{\theta }})}{{\rm{\partial }}{\theta }_{j}}}{\hat{{\rm{\Pi }}}}_{{l}_{n}}^{(n)}({\boldsymbol{\theta }}){\hat{\rho }}^{[n]})\,{\rm{T}}{\rm{r}}\,({\hat{{\rm{\Pi }}}}_{{l}_{m}}^{(m)\dagger }({\boldsymbol{\theta }}){\textstyle \tfrac{{\rm{\partial }}{\hat{{\rm{\Pi }}}}_{{l}_{m}}^{(m)}({\boldsymbol{\theta }})}{{\rm{\partial }}{\theta }_{k}}}{\hat{\rho }}^{[m]})]\\  & = & {J}_{Q}^{jk,[n]}+{J}_{Q}^{jk,[n,m]}.\end{array}$$

This was the case for unital channel of the form considered earlier. Notice that if the initial probe state is maximally mixed, i.e. $\hat{\rho }={1}_{{2}^{N}}/{2}^{N}$, we get $\hat{\rho }({\boldsymbol{\theta }})={1}_{{2}^{N}}/{2}^{N}$ too in that section. This is why quantum correlations are reduced, and cannot be created from any classical correlation in the probe state by the noise in a unital channel^64^, and so the QCRB and the Heisenberg limit are not beaten and the estimator remains unbiased. When there are no correlations or too much quantum correlations in the two-particle reduced density matrix of the initial probe state, the best achievable precision scaling is $1/\sqrt{N}$ with a unital channel, like the unitary channel case. Thus, as long as (53) is satisfied, a noisy channel can at best attain the Heisenberg limit but not beat it, so that the estimator remains unbiased. However, (53) will not be satisfied by non-unital channels, such as local dissipation of the form mentioned earlier, so that quantum correlations can be created from classical correlations in the probe state by noise in the channel. Notice that in this case, if the initial probe state is maximally mixed, the evolved state will not be maximally mixed. Thus, it may be possible to beat the Heisenberg limit with non-unital channels, and the estimator would be biased when the Heisenberg limit is beaten.

Moreover, the fact that dissonance is more robust to decoherence than entanglement^98^ suggests that it is more probable to attain the Heisenberg limit with a mixed state input than a pure entangled state input to a unital channel. In fact, it may not be possible at all to attain the Heisenberg limit with an input pure entangled state because of entanglement sudden death^99,100^. Furthermore, since dissonance can grow and give rise to entanglement in the presence of dissipation, it is more probable to attain or surpass the Heisenberg limit with a mixed state input than a pure entangled state input to a non-unital channel. In fact, it is never possible to attain or surpass the Heisenberg limit with an input pure entangled state because of no initial classical correlations and entanglement sudden death. On the other hand, the fact that entanglement is the intrinsic and minimal discord capturing nonlocal quantum correlations, as opposed to dissonance, which is the extrinsic discord capturing local quantum correlations that cannot be shared^101,102^, is the reason why the Heisenberg limit can be surpassed only when entanglement and not just dissonance is generated in a non-unital channel fed with an input mixed probe state.

In summary, it may appear that noisy quantum states or channels may require the same or less resources to achieve as much as noiseless quantum states or channels, by exploiting additional resources from the environment. That is why, the overall resources required by the noisy cases in the enlarged noiseless system plus bath space are the same as those known to be required by the noiseless cases in the system space alone. However, any channel can be expressed by Kraus operators, which has the same effect as performing a measurement and discarding the result. To have a measurement on a pure state that is the same as the measurement of the pure state after noise, one would just need to have a POVM that combines the POVM elements used for the mixed state with the Kraus operators of the channel, without requiring any extra resource. Thus, a precision scaling of 1/*N*^2*γ*^ can, in principle, be achieved with a pure initial probe state evolving through a unitary channel, described by *γ*-particle Hamiltonians, by using a POVM, that combines the POVM elements used here with the *γ*-particle Kraus operators of the noisy channel and the Kraus operators used to prepare the initial mixed probe state considered here. Thus, entangling measurements^11^ may also contribute to a precision scaling surpassing the Heisenberg limit, unlike as noted earlier. Similarly, a precision scaling of 1/*N*^2*γ*^ can, in principle, be also achieved with a mixed initial probe state evolving through a unitary channel, described by *γ*-particle Hamiltonians, by using a POVM, that combines the POVM elements used here with the *γ*-particle Kraus operators of the noisy channel considered here. But using entangling measurements with our noisy channel, it is possible to obtain even better precision scaling, so the noisy case is still superior.

Nonetheless, although it may likewise seem that it should be possible too to achieve a precision scaling of 1/*N*^2*γ*^ with a pure initial probe state evolving through the noisy channel, described by *γ*-particle Kraus operators, by using a POVM, obtained by combining the POVM elements used here with the Kraus operators used to prepare the initial mixed probe state from the pure state, that is not true even if the initial pure probe state is maximally entangled and/or if the channel is non-unital. This is because of no initial classical or local quantum correlations in the probe state and sudden death of any entanglement in the probe state caused by the noise in the channel, as discussed earlier. This is the distinct important advantage, unique to mixed state metrology^69^.

## Conclusion

We studied fundamental quantum limits in noisy quantum multiparameter estimation using a quantum Fisher information matrix (QFIM) defined in terms of anti-symmetric logarithmic derivatives (ALDs), that lend a convenient way to study noisy metrology. We presented a QFIM for multiparameter estimation using a mixed probe state evolving unitarily. We then considered a mixed state evolving via a noisy channel, and presented an upper bound to the QFIM for this general-most case.

We found that the bounds are such that the quantum enhancement in the estimation precision is provided by the two-particle reduced density matrices and the attainability of the quantum enhancement is solely determined by the one-particle reduced density matrices of the initial probe state, when the channel is described by one-particle evolution operators. We showed conditions and accordingly measurements to saturate these explicitly computable bounds (e.g. in terms of the Kraus operators of the channel), not known to exist with conventional symmetric logarithmic derivatives (SLDs) for these general-most cases. We saw that the Heisenberg limit can be achieved even in these most general noisy cases.

Moreover, for the most part of the past century since the inception of quantum physics, weird quantum phenomena, such as superposition and entanglement, were perceived as bugs, until the 80s when the scientists started to exploit them as features^103^. Today, the biggest hurdle to quantum technologies, e.g. in building a scalable quantum computer, is noise. The results here suggest that some noise in the initial probe state or the quantum channel can actually serve as a feature rather than a bug, because we saw that the achievable estimation precision scaling in the presence of noise is not possible in the absence of any noise in the initial probe state or the quantum channel. Noise in the initial probe state or the channel provides with a quantum advantage by introducing quantum correlations into the system. However, too much noise in the initial probe state or the channel is detrimental, since it introduces too much quantum correlations into the system, and, in turn, harms the quantum advantage achievable with *N* parallel resources.

Furthermore, we found that it is possible to beat the Heisenberg limit by exploiting the noise in the quantum channel. The fundamental super-Heisenberg precision limit for non-unitary channel is then determined by two-particle reduced density operators of the evolved probe state being maximally entangled and one-particle reduced density operators being maximally mixed, and corresponds to a precision scaling of 1/*N*^2^, achieved with one-particle Kraus operators. Further, using *γ*-particle (instead of one-particle) Kraus operators for a noisy channel, where *γ* > 1, the best scaling of 1/*N*^2*γ*^ can be attained, that is known to be only possible with 2*γ*-particle Hamiltonians for a noiseless channel. Such a precision scaling can be achieved with an initial pure or mixed probe state evolving through a unitary channel without requiring additional resources, but not with an initial pure probe state evolving through a noisy channel.

## Methods

### Outline of the proof for ${\boldsymbol{\nu }}{\boldsymbol{V}}\,[\mathop{{\boldsymbol{\theta }}}\limits^{ \sim }({\boldsymbol{m}})]{\boldsymbol{\ge }}{[{{\boldsymbol{J}}}_{{\boldsymbol{C}}}({\boldsymbol{\theta }})]}^{-{\bf{1}}}{\boldsymbol{\ge }}{[{{\boldsymbol{J}}}_{{\boldsymbol{Q}}}({\boldsymbol{\theta }})]}^{-{\bf{1}}}$

Here, we provide an outline of the proof for (7), adapted from ref.^51^ for our case here. Please see Section I of Supplementary Information.

Differentiating (1) with respect to *θ*_*k*_, we get

54 $$\begin{array}{c}{\delta }_{jk}=\sum _{m}\,({\mathop{\theta }\limits^{ \sim }}_{j}(m)-{\theta }_{j})\frac{{\rm{\partial }}p(m|{\boldsymbol{\theta }})}{{\rm{\partial }}{\theta }_{k}}={\rm{R}}{\rm{e}}\,\sum _{m}\,({\mathop{\theta }\limits^{ \sim }}_{j}(m)-{\theta }_{j})\,{\rm{T}}{\rm{r}}\,[{\hat{P}}_{m}{\hat{L}}_{k}\hat{\rho }({\boldsymbol{\theta }})].\end{array}$$

For arbitrary real column vectors ***u***, ***v*** and ***w***, following ref. ^51^, since *ν* ≥ 1, we get ${{\boldsymbol{v}}}^{T}{\boldsymbol{u}}={\sum }_{j}\,{u}_{j}{v}_{j}\le {A}^{T}B$, ${{\boldsymbol{w}}}^{T}{\boldsymbol{u}}={\sum }_{k}\,{u}_{k}{w}_{k}\le {\rm{Re}}\,[\mathrm{Tr}\,({C}^{\dagger }D)]$, where ${A}^{T}={\sum }_{k}\,{v}_{k}\frac{\partial p(m|{\boldsymbol{\theta }})}{\partial {\theta }_{k}}\frac{1}{\sqrt{p(m|{\boldsymbol{\theta }})}}$, $B={\sum }_{j}\,{u}_{j}\,({\tilde{\theta }}_{j}(m)-{\theta }_{j})\,\sqrt{\nu }\sqrt{p(m|{\boldsymbol{\theta }})}$, ${C}^{\dagger }={\sum }_{l}\,{w}_{l}\sqrt{{\hat{P}}_{m}}{\hat{L}}_{l}\sqrt{\hat{\rho }({\boldsymbol{\theta }})}$, and $D={\sum }_{j}\,{u}_{j}\,({\tilde{\theta }}_{j}(m)-{\theta }_{j})\,\sqrt{\nu }\sqrt{\hat{\rho }({\boldsymbol{\theta }})}\sqrt{{\hat{P}}_{m}}$. Assuming ***v***^*T*^ ***u*** and ***w***^*T*^ ***u*** as positive, we get

55 $$\begin{array}{rcl}{({{\boldsymbol{v}}}^{T}{\boldsymbol{u}})}^{2} & \le  & {({A}^{T}B)}^{2}\le ({A}^{T}A)\,({B}^{T}B),\\ {({{\boldsymbol{w}}}^{T}{\boldsymbol{u}})}^{2} & \le  & |{\rm{Tr}}\,({C}^{\dagger }D){|}^{2}\le {\rm{Tr}}\,({C}^{\dagger }C)\,{\rm{Tr}}\,({D}^{\dagger }D),\end{array}$$

where the second inequalities in both lines are Schwarz inequalities.

Now, note that *A*^*T*^*A* = ***v***^*T*^ *J*_*C*_***v***, where *J*_*C*_ is a real, symmetric and positive semidefinite classical Fisher information matrix (FIM) as defined in (4), ${\rm{Tr}}({C}^{\dagger }C)={{\boldsymbol{w}}}^{T}{J}_{Q}{\boldsymbol{w}}$, where *J*_*Q*_ is a real, symmetric and positive semidefinite quantum Fisher information matrix (QFIM) as defined in (6), and ${B}^{T}B={\rm{Tr}}\,({D}^{\dagger }D)={{\boldsymbol{u}}}^{T}\nu {\rm{\Sigma }}{\boldsymbol{u}}$, where ${\rm{\Sigma }}:\,=V\,[\tilde{{\boldsymbol{\theta }}}(m)]$ is the estimation error covariance matrix as defined in (2). Substituting these in (55), we find that $({{\boldsymbol{v}}}^{T}{J}_{C}{\boldsymbol{v}})\,({{\boldsymbol{u}}}^{T}\nu \Sigma {\boldsymbol{u}})\ge ({{\boldsymbol{v}}}^{T}{\boldsymbol{u}})\,({{\boldsymbol{u}}}^{T}{\boldsymbol{v}})$, and $({{\boldsymbol{w}}}^{T}{J}_{Q}{\boldsymbol{w}})\,({{\boldsymbol{u}}}^{T}\nu {\rm{\Sigma }}{\boldsymbol{u}})\ge ({{\boldsymbol{w}}}^{T}{\boldsymbol{u}})\,({{\boldsymbol{u}}}^{T}{\boldsymbol{w}})$. Setting ${\boldsymbol{v}}={J}_{C}^{-1}{\boldsymbol{u}}$ implies that ${{\boldsymbol{u}}}^{T}\,(\nu {\rm{\Sigma }}-{J}_{C}^{-1})\,{\boldsymbol{u}}\ge 0$ for arbitrary real vectors ***u***. Since ${\rm{\Sigma }}-{J}_{C}^{-1}$ is real and symmetric, this implies that $\nu {\rm{\Sigma }}-{J}_{C}^{-1}$ is positive semidefinite. Also, setting ${\boldsymbol{w}}={J}_{Q}^{-1}{\boldsymbol{u}}$ implies that ${{\boldsymbol{u}}}^{T}(\nu {\rm{\Sigma }}-{J}_{Q}^{-1})\,{\boldsymbol{u}}\ge 0$. Since ${\rm{\Sigma }}-{J}_{Q}^{-1}$ is real and symmetric, this implies that $\nu {\rm{\Sigma }}-{J}_{Q}^{-1}$ is positive semidefinite.

We now take ***v*** = ***w***. Then, ${{\boldsymbol{v}}}^{T}{\boldsymbol{u}}$ ≤ ${A}^{T}B$ = ${\rm{Re}}\,[\mathrm{Tr}\,({C}^{\dagger }D)]\Rightarrow ({{\boldsymbol{v}}}^{T}{\boldsymbol{u}})\,({{\boldsymbol{u}}}^{T}{\boldsymbol{v}})\le {({A}^{T}B)}^{2}$ ≤ $|{\rm{Tr}}\,({C}^{\dagger }D){|}^{2}$ ≤ ${\rm{Tr}}\,({C}^{\dagger }C)$ ${\rm{Tr}}\,({D}^{\dagger }D)=({{\boldsymbol{v}}}^{T}{J}_{Q}{\boldsymbol{v}})\,({{\boldsymbol{u}}}^{T}\nu {\rm{\Sigma }}{\boldsymbol{u}})$. Setting ${\boldsymbol{v}}={J}_{C}^{-1}{\boldsymbol{u}}$ gives ${({{\boldsymbol{u}}}^{T}{J}_{C}^{-1}{\boldsymbol{u}})}^{2}\le ({{\boldsymbol{u}}}^{T}{J}_{C}^{-1}{J}_{Q}{J}_{C}^{-1}{\boldsymbol{u}})\,({{\boldsymbol{u}}}^{T}\nu {\rm{\Sigma }}{\boldsymbol{u}})$. Since ${{\boldsymbol{u}}}^{T}(\nu {\rm{\Sigma }}-{J}_{C}^{-1})\,{\boldsymbol{u}}\ge 0$, we get: ${{\boldsymbol{u}}}^{T}{J}_{C}^{-1}{\boldsymbol{u}}$ ≤ ${{\boldsymbol{u}}}^{T}{J}_{C}^{-1}{J}_{Q}{J}_{C}^{-1}{\boldsymbol{u}}\Rightarrow {J}_{C}^{-1}$ ≤ ${J}_{C}^{-1}{J}_{Q}{J}_{C}^{-1}\Rightarrow {J}_{C}^{-1}$ ≥ ${J}_{Q}^{-1}$. Thus, we have (7).

### Condition to saturate ALD-based QCRB

Here, we provide an outline of the proof that the ALD-based QCRB (7) can be saturated when (8) is satisfied for every pair of ALDs. The proof is directly adapted from ref. ^56^ for ALDs. Please see Section II of Supplementary Information for the full proof.

The proof relies on the fact that it is enough to show that the QFIM bound is equivalent to the Holevo bound when (8) is satisfied, because the Holevo bound is a tighter bound, known to be asymptotically saturable.

Given the anti-Hermitian operators ${\hat{L}}_{k}$ satisfying (5) and the QFIM *J*_*Q*_ from (6), the bound (7) implies that for a given positive definite cost matrix *G*, the estimation cost is bounded by^56,104^:

56 $${\rm{t}}{\rm{r}}\,(G\nu V\,[\mathop{{\boldsymbol{\theta }}}\limits^{ \sim }(m)])\ge {\rm{t}}{\rm{r}}\,(G{J}_{Q}^{-1})=\mathop{min}\limits_{\{{\hat{X}}_{j}\}}\,{\rm{t}}{\rm{r}}\,(G{\rm{R}}{\rm{e}}W),$$

where tr denotes the trace of a matrix in distinction from Tr for an operator. Then, the achievable estimation uncertainty is lower-bounded by the Holevo Cramér-Rao bound^56,104^:

57 $${\rm{t}}{\rm{r}}\,(G\nu V\,[\mathop{{\boldsymbol{\theta }}}\limits^{ \sim }(m)])\ge \mathop{min}\limits_{\{{\hat{X}}_{j}\}}\,\{{\rm{t}}{\rm{r}}\,(G{\rm{R}}{\rm{e}}W)+\parallel G{\rm{I}}{\rm{m}}W{\parallel }_{1}\},$$

where ||·||_1_ is the operator trace norm, ${W}_{jk}={\rm{Tr}}\,({\hat{X}}_{j}^{\dagger }{\hat{X}}_{k}\hat{\rho }({\boldsymbol{\theta }}))$^104^, and the minimization is performed over the anti-Hermitian operators ${\hat{X}}_{j}$ satisfying $\frac{1}{2}\,{\rm{Tr}}\,[({\hat{X}}_{j}^{\dagger }{\hat{L}}_{k}+{\hat{L}}_{k}^{\dagger }{\hat{X}}_{j})\hat{\rho }(\theta )]={\delta }_{jk}$. Then, the solution to the minimization problem in (56) is^56^:

58 $${\hat{X}}_{j}=\sum _{k}\,{({J}_{Q}^{-1})}_{jk}{\hat{L}}_{k},$$

for which the second term in (57) becomes zero, when (8) is satisfied, such that the Holevo bound coincides with the QFIM bound.

### POVM to saturate ALD-based QCRB

Here, we provide an outline of the proof that the set of POVMs from (9) indeed saturates the ALD-based QCRB (7), if the condition (8) holds for every pair of ALDs.

Consider that our initial state is pure, i.e. $\hat{\rho }=|\psi \rangle \langle \psi |$. Note that, given the way we choose our ALDs, the ALD-based QCRB coincides with the SLD-based QCRB for pure state input and unitary channel. Then, based on refs^4,5^, the set of POVMs $\{{\hat{P}}_{m1}\}$ of cardinality *q* + 2, comprising the *q* + 1 elements,

59 $$\begin{array}{ccc}{\hat{P}}_{0} & = & \hat{U}({\boldsymbol{\theta }})|\psi \rangle \langle \psi |{\hat{U}}^{\dagger }({\boldsymbol{\theta }}),\\ {\hat{P}}_{m} & = & \frac{{\rm{\partial }}\hat{U}({\boldsymbol{\theta }})}{{\rm{\partial }}{\theta }_{m}}|\psi \rangle \langle \psi |\frac{{\rm{\partial }}{\hat{U}}^{\dagger }({\boldsymbol{\theta }})}{{\rm{\partial }}{\theta }_{m}}\,{\rm{\forall }}m=1,\ldots ,q,\end{array}$$

together with one element that accounts for the normalisation, saturates the ALD-based QCRB, provided (8) is satisfied for every pair of ALDs. If it is not immediately obvious from refs^4,5^ that the set of POVMs $\{{\hat{P}}_{m1}\}$ indeed saturates the QCRB, when (8) is satisfied, then please see Section V of Supplementary Information.

However, considering our initial state is not pure, we can purify it by extending the system *S* space, introducing ancillas *S*′, and can then apply (59) to the pure state in the initial enlarged space *S* + *S*′. Since the operators $\hat{U}({\boldsymbol{\theta }})$ and $\frac{\partial \hat{U}({\boldsymbol{\theta }})}{\partial {\theta }_{k}}$ do not act on *S*′ (e.g. see ref.^26^), the set of POVMs $\{{\hat{P}}_{m1}\}$ can saturate the ALD-based QCRB in the *S* + *S*′ space, if the set of POVMs $\{{\hat{P}}_{m2}\}$ of cardinality *q* + 2, comprising the *q* + 1 elements,

60 $$\begin{array}{ccc}{\hat{P}}_{0} & = & \hat{U}({\boldsymbol{\theta }})\hat{\rho }{\hat{U}}^{\dagger }({\boldsymbol{\theta }}),\\ {\hat{P}}_{m} & = & \frac{{\rm{\partial }}\hat{U}({\boldsymbol{\theta }})}{{\rm{\partial }}{\theta }_{m}}\hat{\rho }{\hat{U}}^{\dagger }({\boldsymbol{\theta }})+\hat{U}({\boldsymbol{\theta }})\hat{\rho }\frac{{\rm{\partial }}{\hat{U}}^{\dagger }({\boldsymbol{\theta }})}{{\rm{\partial }}{\theta }_{m}}\,{\rm{\forall }}m=1,\ldots ,q,\end{array}$$

along with one element that accounts for the normalisation, saturates the ALD-based QCRB in the *S* space, provided (8) is satisfied for every pair of ALDs. Please see Section VI of Supplementary Information.

### Outline of the proof for *C*_*Q*_(***θ***) ≥ *J*_*Q*_(***θ***)

Here, we provide an outline of the proof for *C*_*Q*_(***θ***) ≥ *J*_*Q*_(***θ***) for $\hat{\rho }({\boldsymbol{\theta }})$. Please see Section V of Supplementary Information for the full proof.

The Bures metric *d*_*B*_ and Bures distance *D*_*B*_ are defined and related to the fidelity *F* as follows^28,105^:

61 $$\begin{array}{c}{d}_{B}^{2}\,(\hat{\rho }({\boldsymbol{\theta }}),\hat{\rho }({\boldsymbol{\theta }}+{\boldsymbol{\epsilon }}))={D}_{B}^{2}\,(\hat{\rho }({\boldsymbol{\theta }}),\hat{\rho }({\boldsymbol{\theta }}+{\boldsymbol{\epsilon }}))=2\,[1-F\,(\hat{\rho }({\boldsymbol{\theta }}),\hat{\rho }({\boldsymbol{\theta }}+{\boldsymbol{\epsilon }}))]=\frac{1}{2}\,\sum _{j,k}\,{{\boldsymbol{\epsilon }}}_{j}{{\boldsymbol{\epsilon }}}_{k}{\rm{T}}{\rm{r}}\,[\frac{{\hat{L}}_{j}^{\dagger }{\hat{L}}_{k}+{\hat{L}}_{k}^{\dagger }{\hat{L}}_{j}}{2}\hat{\rho }({\boldsymbol{\theta }})],\end{array}$$

where the operators ${\hat{L}}_{k}$ are anti-Hermitian and satisfy (5) and ***θ*** is assumed to be the actual value of the vector of unknown parameters, ${\boldsymbol{\epsilon }}$ is an infinitesimal increment in ***θ***, and $0\le F({\hat{\rho }}_{1},{\hat{\rho }}_{2})={\rm{Tr}}\,(\sqrt{\sqrt{{\hat{\rho }}_{1}}{\hat{\rho }}_{2}\sqrt{{\hat{\rho }}_{1}}})\le 1$ is the Bures fidelity between two given states ${\hat{\rho }}_{1}$ and ${\hat{\rho }}_{2}$^20,22,24,105–107^. We have from (61):

62 $$F\,(\hat{\rho }({\boldsymbol{\theta }}),\hat{\rho }({\boldsymbol{\theta }}+{\boldsymbol{\epsilon }}))=1-\frac{1}{4}\,\sum _{j,k}\,{{\boldsymbol{\epsilon }}}_{j}{{\boldsymbol{\epsilon }}}_{k}{J}_{Q}^{jk}({\boldsymbol{\theta }}).$$

Now, since fidelity is non-decreasing with respect to partial trace^24,106,108,109^, we have:

63 $$F\,(\hat{\rho }({\boldsymbol{\theta }}),\hat{\rho }({\boldsymbol{\theta }}+{\boldsymbol{\epsilon }}))=F\,({{\rm{T}}{\rm{r}}}_{B}\,[{\hat{\rho }}_{SB}({\boldsymbol{\theta }})],{{\rm{T}}{\rm{r}}}_{B}\,[{\hat{\rho }}_{SB}({\boldsymbol{\theta }}+{\boldsymbol{\epsilon }})])\ge F\,({\hat{\rho }}_{SB}({\boldsymbol{\theta }}),{\hat{\rho }}_{SB}({\boldsymbol{\theta }}+{\boldsymbol{\epsilon }}))=1-\frac{1}{4}\,\sum _{j,k}\,{{\boldsymbol{\epsilon }}}_{j}{{\boldsymbol{\epsilon }}}_{k}{C}_{Q}^{jk}({\boldsymbol{\theta }}).$$

Clearly, from (62) and (63), we have (like in ref.^22^):

64 $${C}_{Q}({\boldsymbol{\theta }})\ge {J}_{Q}({\boldsymbol{\theta }}).$$

Extending the argument from ref. ^26^ to the multiparameter case, the equality in (64) is achieved by minimizing *C*_*Q*_(***θ***) over all Kraus representations of the quantum channel. Hence, there exist an infinitude of Kraus representations of the quantum channel that lead to *C*_*Q*_(***θ***) = *J*_*Q*_(***θ***). An alternative argument for (64) to hold is that quantum Fisher information (for both single and multiparameter cases) is non-increasing with respect to partial trace^31,110^.

### Condition and POVM to saturate Upper Bound to QFIM

Here, we provide an outline of the proof that the upper bound (32) to the QFIM may be saturated, if the condition (35) holds.

If our initial probe state is pure, i.e. $\hat{\rho }=|\psi \rangle \langle \psi |$, then the unitary evolution ${\hat{U}}_{SB}({\boldsymbol{\theta }})$ in the *S* + *B* space can be considered equivalent to the output impure state ${\sum }_{l}\,{\hat{{\rm{\Pi }}}}_{l}({\boldsymbol{\theta }})|\psi \rangle \langle \psi |{\hat{{\rm{\Pi }}}}_{l}^{\dagger }({\boldsymbol{\theta }})$ of the noisy channel in the system *S* space, subsequently purified by extending the *S* space, introducing ancillas *B*. Then, (8) implies that the condition

65 $${\rm{I}}{\rm{m}}\,[{\rm{T}}{\rm{r}}\,\{(\frac{{\rm{\partial }}{\hat{U}}_{SB}^{\dagger }({\boldsymbol{\theta }})}{{\rm{\partial }}{\theta }_{j}}\frac{{\rm{\partial }}{\hat{U}}_{SB}({\boldsymbol{\theta }})}{{\rm{\partial }}{\theta }_{k}})\,(\hat{\rho }\otimes |0\rangle \langle 0|)\}]=0\,{\rm{\forall }}j,k$$

saturates the ALD-based QCRB in the final enlarged *S* + *B* space. This is possible only if (35) saturates the upper bound (32) to the QFIM in the *S* space, since the operators $\frac{\partial {\hat{{\rm{\Pi }}}}_{l}({\boldsymbol{\theta }})}{\partial {\theta }_{k}}$ do not act on *B*.

However, even when our initial probe state is impure, we can purify it by extending the system *S* space, introducing ancillas *S*′, and can then apply (35) to the pure state in the initial enlarged space *S* + *S*′. Since the operators $\frac{\partial {\hat{{\rm{\Pi }}}}_{l}({\boldsymbol{\theta }})}{\partial {\theta }_{k}}$ do not act on *S*′, we must have (35) to saturate the upper bound (32) to the QFIM in the *S* space. Please see Section VII of Supplementary Information.

Next, we prove that the set of POVMs from (36) indeed saturates the upper bound (32) to the QFIM, if the condition (35) holds.

Consider again that our initial probe state is pure, i.e. $\hat{\rho }=|\psi \rangle \langle \psi |$. Then, extending (59) to our case here, the set of POVMs {${\hat{P}}_{n1}$} of cardinality *q* + 2, comprising the *q* + 1 elements,

66 $$\begin{array}{ccc}{\hat{P}}_{0} & = & {\hat{U}}_{SB}({\boldsymbol{\theta }})|\psi \rangle \langle \psi |{\hat{U}}_{SB}^{\dagger }({\boldsymbol{\theta }}),\\ {\hat{P}}_{m} & = & \frac{{\rm{\partial }}{\hat{U}}_{SB}({\boldsymbol{\theta }})}{{\rm{\partial }}{\theta }_{m}}|\psi \rangle \langle \psi |\frac{{\rm{\partial }}{\hat{U}}_{SB}^{\dagger }({\boldsymbol{\theta }})}{{\rm{\partial }}{\theta }_{m}}\,{\rm{\forall }}m=1,\ldots ,q,\end{array}$$

together with one element that accounts for the normalisation, saturates the ALD-based QCRB in the final *S* + *B* space, provided (65) is satisfied. Now, since the operators ${\hat{{\rm{\Pi }}}}_{l}({\boldsymbol{\theta }})$ and $\frac{\partial {\hat{{\rm{\Pi }}}}_{l}({\boldsymbol{\theta }})}{\partial {\theta }_{k}}$ do not act on *B*, the set of POVMs $\{{\hat{P}}_{n1}\}$ can saturate the ALD-based QCRB in the final *S* + *B* space, if the set of POVMs $\{{\hat{P}}_{n2}\}$ of cardinality *q* + 2, comprising the *q* + 1 elements,

67 $$\begin{array}{ccc}{\hat{P}}_{0} & = & \hat{\rho }({\boldsymbol{\theta }})=\sum _{l}\,{\hat{{\rm{\Pi }}}}_{l}({\boldsymbol{\theta }})|\psi \rangle \langle \psi |{\hat{{\rm{\Pi }}}}_{l}^{\dagger }({\boldsymbol{\theta }}),\\ {\hat{P}}_{m} & = & \frac{{\rm{\partial }}\hat{\rho }({\boldsymbol{\theta }})}{{\rm{\partial }}{\theta }_{m}}=\sum _{l}\,[\frac{{\rm{\partial }}{\hat{{\rm{\Pi }}}}_{l}({\boldsymbol{\theta }})}{{\rm{\partial }}{\theta }_{m}}|\psi \rangle \langle \psi |{\hat{{\rm{\Pi }}}}_{l}^{\dagger }({\boldsymbol{\theta }})\,+\,{\hat{{\rm{\Pi }}}}_{l}({\boldsymbol{\theta }})|\psi \rangle \langle \psi |\frac{{\rm{\partial }}{\hat{{\rm{\Pi }}}}_{l}^{\dagger }({\boldsymbol{\theta }})}{{\rm{\partial }}{\theta }_{m}}]\,{\rm{\forall }}m=1,\ldots ,q,\end{array}$$

together with one element that accounts for the normalisation, saturates the upper bound (32) to the QFIM in the *S* space, provided (35) is satisfied. Please see Section VIII of Supplementary Information.

Further, when the initial probe state in *S* space is impure, we can apply (67) to the pure state in the initial enlarged space *S* + *S*′. Since the operators ${\hat{{\rm{\Pi }}}}_{l}({\boldsymbol{\theta }})$ and $\frac{\partial {\hat{{\rm{\Pi }}}}_{l}({\boldsymbol{\theta }})}{\partial {\theta }_{k}}$ do not act on *S*′, the set of POVMs $\{{\hat{P}}_{n2}\}$ can saturate the upper bound (32) to the QFIM in the *S* + *S*′ space, if the set of POVMs $\{{\hat{P}}_{n3}\}$ of cardinality *q* + 2, comprising the *q* + 1 elements,

68 $$\begin{array}{ccc}{\hat{P}}_{0} & = & \hat{\rho }({\boldsymbol{\theta }})=\sum _{l}\,{\hat{{\rm{\Pi }}}}_{l}({\boldsymbol{\theta }})\hat{\rho }{\hat{{\rm{\Pi }}}}_{l}^{\dagger }({\boldsymbol{\theta }}),\\ {\hat{P}}_{m} & = & \frac{{\rm{\partial }}\hat{\rho }({\boldsymbol{\theta }})}{{\rm{\partial }}{\theta }_{m}}=\sum _{l}\,[\frac{{\rm{\partial }}{\hat{{\rm{\Pi }}}}_{l}({\boldsymbol{\theta }})}{{\rm{\partial }}{\theta }_{m}}\hat{\rho }{\hat{{\rm{\Pi }}}}_{l}^{\dagger }({\boldsymbol{\theta }})\\  &  & +\,{\hat{{\rm{\Pi }}}}_{l}({\boldsymbol{\theta }})\hat{\rho }\frac{{\rm{\partial }}{\hat{{\rm{\Pi }}}}_{l}^{\dagger }({\boldsymbol{\theta }})}{{\rm{\partial }}{\theta }_{m}}]\,{\rm{\forall }}m=1,\ldots ,q,\end{array}$$

along with one element that accounts for the normalisation, saturates the upper bound (32) to the QFIM in the *S* space, when (35) is satisfied. Please see Section IX of Supplementary Information.

### Beating the Heisenberg Limit with Noise in Channel

Here, we provide an outline of the proof that the Heisenberg limit can be beaten with a non-unital channel, when (35) is satisfied. Please see Section X of Supplementary Information for the full proof.

First, note that

69 $$\begin{array}{cccc} & \hat{\rho }({\boldsymbol{\theta }}) & = & \sum _{l}\,{\hat{{\rm{\Pi }}}}_{l}({\boldsymbol{\theta }})\hat{\rho }{\hat{{\rm{\Pi }}}}_{l}^{\dagger }({\boldsymbol{\theta }})\\ \Rightarrow  & \frac{{\rm{\partial }}\hat{\rho }({\boldsymbol{\theta }})}{{\rm{\partial }}{\theta }_{k}} & = & \sum _{l}[\frac{{\rm{\partial }}{\hat{{\rm{\Pi }}}}_{l}({\boldsymbol{\theta }})}{{\rm{\partial }}{\theta }_{k}}\hat{\rho }{\hat{{\rm{\Pi }}}}_{l}^{\dagger }({\boldsymbol{\theta }})+{\hat{{\rm{\Pi }}}}_{l}({\boldsymbol{\theta }})\hat{\rho }\frac{{\rm{\partial }}{\hat{{\rm{\Pi }}}}_{l}^{\dagger }({\boldsymbol{\theta }})}{{\rm{\partial }}{\theta }_{k}}].\end{array}$$

Next, (35) saturates an ALD-based QCRB, corresponding to:

70 $$\frac{{\rm{\partial }}\hat{\rho }({\boldsymbol{\theta }})}{{\rm{\partial }}{\theta }_{k}}=\frac{1}{2}\,[{\hat{O}}_{k}\hat{\rho }+\hat{\rho }{\hat{O}}_{k}^{\dagger }]=\sum _{l}\,[\frac{{\rm{\partial }}{\hat{{\rm{\Pi }}}}_{l}({\boldsymbol{\theta }})}{{\rm{\partial }}{\theta }_{k}}\hat{\rho }+\hat{\rho }\frac{{\rm{\partial }}{\hat{{\rm{\Pi }}}}_{l}^{\dagger }({\boldsymbol{\theta }})}{{\rm{\partial }}{\theta }_{k}}],$$

where the ALDs are chosen to be ${\hat{O}}_{k}=2\,{\sum }_{l}\,\frac{\partial {\hat{{\rm{\Pi }}}}_{l}({\boldsymbol{\theta }})}{\partial {\theta }_{k}}$. Strictly speaking, this is not a valid ALD, since it is not a function of the probe state. Moreover, (70) is not expressed in terms of the evolved probe state. However, it suffices to consider the above for our proof here for simplicity without loss of generality.

Assume that when (35) is satisfied, the upper bound (32) to the QFIM equals the actual QFIM. This is possible, when we have:

$$\sum _{l}\,[{\textstyle \tfrac{{\rm{\partial }}{\hat{{\rm{\Pi }}}}_{l}({\boldsymbol{\theta }})}{{\rm{\partial }}{\theta }_{k}}}\hat{\rho }{\hat{{\rm{\Pi }}}}_{l}^{\dagger }({\boldsymbol{\theta }})+{\hat{{\rm{\Pi }}}}_{l}({\boldsymbol{\theta }})\hat{\rho }{\textstyle \tfrac{{\rm{\partial }}{\hat{{\rm{\Pi }}}}_{l}^{\dagger }({\boldsymbol{\theta }})}{{\rm{\partial }}{\theta }_{k}}}]=\sum _{l}\,[{\textstyle \tfrac{{\rm{\partial }}{\hat{{\rm{\Pi }}}}_{l}({\boldsymbol{\theta }})}{{\rm{\partial }}{\theta }_{k}}}\hat{\rho }+\hat{\rho }{\textstyle \tfrac{{\rm{\partial }}{\hat{{\rm{\Pi }}}}_{l}^{\dagger }({\boldsymbol{\theta }})}{{\rm{\partial }}{\theta }_{k}}}],$$

following from (69) and (70).

Now, the above requires the following condition to be satisfied:

$$\sum _{l}\,\frac{\partial {\hat{{\rm{\Pi }}}}_{l}({\boldsymbol{\theta }})}{\partial {\theta }_{k}}\hat{\rho }{\hat{{\rm{\Pi }}}}_{l}^{\dagger }({\boldsymbol{\theta }})=\sum _{l}\,\frac{\partial {\hat{{\rm{\Pi }}}}_{l}({\boldsymbol{\theta }})}{\partial {\theta }_{k}}\hat{\rho },$$

which, in turn, yields

$$\sum _{l}\,{\rm{T}}{\rm{r}}\,[({\textstyle \tfrac{{\rm{\partial }}{\hat{{\rm{\Pi }}}}_{l}^{\dagger }({\boldsymbol{\theta }})}{{\rm{\partial }}{\theta }_{j}}}{\hat{{\rm{\Pi }}}}_{l}({\boldsymbol{\theta }}){\hat{{\rm{\Pi }}}}_{l}^{\dagger }({\boldsymbol{\theta }}){\textstyle \tfrac{{\rm{\partial }}{\hat{{\rm{\Pi }}}}_{l}({\boldsymbol{\theta }})}{{\rm{\partial }}{\theta }_{k}}})\hat{\rho }]=\sum _{l}\,{\rm{T}}{\rm{r}}\,[({\textstyle \tfrac{{\rm{\partial }}{\hat{{\rm{\Pi }}}}_{l}^{\dagger }({\boldsymbol{\theta }})}{{\rm{\partial }}{\theta }_{j}}}{\textstyle \tfrac{{\rm{\partial }}{\hat{{\rm{\Pi }}}}_{l}({\boldsymbol{\theta }})}{{\rm{\partial }}{\theta }_{k}}})\hat{\rho }],$$

which is possible when we have ${\sum }_{l}\,{\hat{{\rm{\Pi }}}}_{l}({\boldsymbol{\theta }}){\hat{{\rm{\Pi }}}}_{l}^{\dagger }({\boldsymbol{\theta }})=1$, i.e. when the channel is unital. Thus, for unital channels the upper bound (32) to the QFIM equals the actual QFIM. Hence, if the channel is non-unital, the upper bound (32) to the QFIM can be strictly larger than the actual QFIM, so that the Heisenberg limit is beaten.

## Supplementary information

Fundamental noisy multiparameter quantum bounds: Detailed Proofs

## References

[1] Genoni MG (2013). Optimal estimation of joint parameters in phase space. Physical Review A.

[2] Vidrighin MD (2014). Joint estimation of phase and phase diffusion for quantum metrology. Nature Communications.

[3] Crowley PJD, Datta A, Barbieri M, Walmsley IA (2014). Tradeoff in simultaneous quantum-limited phase and loss estimation in interferometry. Physical Review A.

[4] Humphreys PC, Barbieri M, Datta A, Walmsley IA (2013). Quantum enhanced multiple phase estimation. Physical Review Letters.

[5] Baumgratz T, Datta A (2016). Quantum enhanced estimation of a multi-dimensional field. Physical Review Letters.

[6] Szczykulska M, Baumgratz T, Datta A (2016). Multiparameter quantum metrology. Advances in Physics: X.

[7] Pezzè L (2017). Optimal measurements for simultaneous quantum estimation of multiple phases. Physical Review Letters.

[8] Gagatsos CN, Branford D, Datta A (2016). Gaussian systems for quantum-enhanced multiple phase estimation. Physical Review A.

[9] Nichols R, Liuzzo-Scorpo P, Knott PA, Adesso G (2018). Multiparameter Gaussian quantum metrology. Physical Review A.

[10] Liu J, Yuan H (2017). Control-enhanced multiparameter quantum estimation. Physical Review A.

[11] Roccia E (2017). Entangling measurements for multiparameter estimation with two qubits. Quantum Science and Technology.

[12] Ciampini MA (2016). Quantum-enhanced multiparameter estimation in multiarm interferometers. Scientific Reports.

[13] Proctor TJ, Knott PA, Dunningham JA (2018). Multiparameter estimation in networked quantum sensors. Physical Review Letters.

[14] Gessner M, Pezzè L, Smerzi A (2018). Sensitivity bounds for multiparameter quantum metrology. Physical Review Letters.

[15] Giovannetti V, Lloyd S, Maccone L (2011). Advances in quantum metrology. Nature Photonics.

[16] Giovannetti V, Lloyd S, Maccone L (2006). Quantum metrology. Physical Review Letters.

[17] Friis N (2017). Flexible resources for quantum metrology. New Journal of Physics.

[18] Giovannetti V, Lloyd S, Maccone L (2004). Quantum-enhanced measurements: Beating the standard quantum limit. Science.

[19] Helstrom, C. W. *Quantum detection and estimation theory, Mathematics in Science and Engineering*. (Academic Press, Massachusetts, 1967).

[20] Paris MGA (2009). Quantum estimation for quantum technology. International Journal of Quantum Information.

[21] Liu N, Cable H (2017). Quantum-enhanced multiparameter estimation for unitary photonic systems. Quantum Science and Technology.

[22] Yue J-D, Zhang Y-R, Fan H (2014). Quantum-enhanced metrology for multiple phase estimation with noise. Scientific Reports.

[23] Wiseman, H. M. & Milburn, G. J. *Quantum Measurement and Control* (Cambridge University Press, 2010).

[24] Nielsen, M. A. & Chuang, I. L. *Quantum Computation and Quantum Information* (Cambridge University Press, 2002).

[25] Braunstein SL, Caves CM, Milburn GJ (1996). Generalized uncertainty relations: Theory, examples, and Lorentz invariance. Annals of Physics.

[26] Escher BM, de Matos Filho RL, Davidovich L (2011). General framework for estimating the ultimate precision limit in noisy quantum-enhanced metrology. Nature Physics.

[27] Szczykulska M, Baumgratz T, Datta A (2017). Reaching for the quantum limits in the simultaneous estimation of phase and phase diffusion. Quantum Science and Technology.

[28] Pasquale AD, Rossini D, Facchi P, Giovannetti V (2013). Quantum parameter estimation affected by unitary disturbance. Physical Review A.

[29] Kołodyński J, Demkowicz-Dobrzański R (2013). Efficient tools for quantum metrology with uncorrelated noise. New Journal of Physics.

[30] Demkowicz-Dobrzański R, Kołodyński J, Guţă M (2012). The elusive Heisenberg limit in quantum-enhanced metrology. Nature Communications.

[31] Demkowicz-Dobrzański R, Maccone L (2014). Using entanglement against noise in quantum metrology. Physical Review Letters.

[32] Demkowicz-Dobrzański R, Czajkowski J, Sekatski P (2017). Adaptive quantum metrology under general Markovian noise. Physical Review X.

[33] Nichols R, Bromley TR, Correa LA, Adesso G (2016). Practical quantum metrology in noisy environments. Physical Review A.

[34] Yao Y, Ge L, Xiao X, Wang X, Sun CP (2014). Multiple phase estimation for arbitrary pure states under white noise. Physical Review A.

[35] Roccia E (2018). Multiparameter quantum estimation of noisy phase shifts. Optica.

[36] Jeske J, Cole JH, Huelga SF (2014). Quantum metrology subject to spatially correlated Markovian noise: restoring the Heisenberg limit. New Journal of Physics.

[37] Sekatski P, Skotiniotis M, Dür W (2016). Dynamical decoupling leads to improved scaling in noisy quantum metrology. New Journal of Physics.

[38] Sekatski P, Skotiniotis M, Kołodyński J, Dür W (2017). Quantum metrology with full and fast quantum control. Quantum.

[39] Falaye BJ (2017). Investigating quantum metrology in noisy channels. Scientific Reports.

[40] Huang Z, Macchiavello C, Maccone L (2018). Noise-dependent optimal strategies for quantum metrology. Physical Review A.

[41] Zhou S, Zhang M, Preskill J, Jiang L (2018). Achieving the Heisenberg limit in quantum metrology using quantum error correction. Nature. Communications.

[42] Chen G (2018). Heisenberg-scaling measurement of the single-photon Kerr non-linearity using mixed states. Nature. Communications.

[43] Demkowicz-Dobrzański R, Jarzyna M, Kołodyński J (2015). Quantum limits in optical interferometry. Progress in Optics.

[44] Benatti F, Alipour S, Rezakhani AT (2014). Dissipative quantum metrology in manybody systems of identical particles. New Journal of Physics.

[45] Beau M, del Campo A (2017). Nonlinear quantum metrology of many-body open systems. Physical Review Letters.

[46] Alipour S, Mehboudi M, Rezakhani AT (2014). Quantum metrology in open systems: Dissipative Cramér-Rao bound. Physical Review Letters.

[47] Yuan H, Fung C-HF (2017). Quantum parameter estimation with general dynamics. npj Quantum Information.

[48] Tsang M (2013). Quantum metrology with open dynamical systems. New Journal of Physics.

[49] Albarelli F, Rossi MAC, Tamascelli D, Genoni MG (2018). Restoring Heisenberg scaling in noisy quantum metrology by monitoring the environment. Quantum.

[50] Haase JF, Smirne A, Huelga SF, Kołodyński J, Demkowicz-Dobrzański R (2018). Precision limits in quantum metrology with open quantum systems. Quantum Measurements and Quantum Metrology.

[51] Tsang M, Wiseman HM, Caves CM (2011). Fundamental quantum limit to waveform estimation. Physical Review Letters.

[52] Fujiwara A, Nagaoka H (1995). Quantum Fisher metric and estimation for pure state models. Physics Letters A.

[53] Fujiwara A, Imai H (2008). A fibre bundle over manifolds of quantum channels and its application to quantum statistics. Journal of Physics A: Mathematical and Theoretical.

[54] Braunstein SL (1992). How large a sample is needed for the maximum likelihood estimator to be approximately Gaussian?. Journal of Physics A: Mathematical and General.

[55] Matsumoto K (2002). A new approach to the Cramér-Rao type bound of the pure-state model. Journal of Physics A: Mathematical and General.

[56] Ragy S, Jarzyna M, Demkowicz-Dobrzański R (2016). Compatibility in multiparameter quantum metrology. Physical Review A.

[57] Wilcox RM (1967). Exponential operators and parameter differentiation in quantum physics. Journal of Mathematical Physics.

[58] Hyllus P, Gühne O, Smerzi A (2010). Not all pure entangled states are useful for sub-shot-noise interferometry. Physical Review A.

[59] Jarzyna M, Demkowicz-Dobrzański R (2015). True precision limits in quantum metrology. New Journal of Physics.

[60] Piani M (2011). All nonclassical correlations can be activated into distillable entanglement. Physical Review Letters.

[61] Horodecki R, Horodecki P, Horodecki M, Horodecki K (2009). Quantum entanglement. Reviews of Modern Physics.

[62] Braun D (2002). Creation of entanglement by interaction with a common heat bath. Physical Review Letters.

[63] Benatti F, Floreanini R, Piani M (2003). Environment induced entanglement in Markovian dissipative dynamics. Physical Review Letters.

[64] Streltsov A, Kampermann H, Bruß D (2011). Behavior of quantum correlations under local noise. Physical Review Letters.

[65] Orieux A (2015). Experimental generation of robust entanglement from classical correlations via local dissipation. Physical Review Letters.

[66] Bollinger JJ, Itano WM, Wineland DJ, Heinzen DJ (1996). Optimal frequency measurements with maximally correlated states. Physical Review A.

[67] Shaji A, Caves CM (2007). Qubit metrology and decoherence. Physical Review A.

[68] Huver SD, Wildfeuer CF, Dowling JP (2008). Entangled Fock states for robust quantum optical metrology, imaging, and sensing. Physical Review A.

[69] Modi K, Cable H, Williamson M, Vedral V (2011). Quantum correlations in mixed-state metrology. Physical Review X.

[70] Luis A (2007). Quantum limits, nonseparable transformations, and nonlinear optics. Physical Review A.

[71] Beltrán J, Luis A (2005). Breaking the Heisenberg limit with inefficient detectors. Physical Review A.

[72] Roy SM, Braunstein SL (2008). Exponentially enhanced quantum metrology. Physical Review Letters.

[73] Rivas A, Luis A (2012). Sub-Heisenberg estimation of non-random phase shifts. New Journal of Physics.

[74] Boixo S, Flammia ST, Caves CM, Geremia JM (2007). Generalized limits for single-parameter quantum estimation. Physical Review Letters.

[75] Woolley MJ, Milburn GJ, Caves CM (2008). Nonlinear quantum metrology using coupled nanomechanical resonators. New Journal of Physics.

[76] Anisimov PM (2010). Quantum metrology with two-mode squeezed vacuum: Parity detection beats the Heisenberg limit. Physical Review Letters.

[77] Boixo S (2008). Quantum metrology: Dynamics versus entanglement. Physical Review Letters.

[78] Boixo S (2008). Quantum-limited metrology with product states. Physical Review A.

[79] Choi S, Sundaram B (2008). Bose-Einstein condensate as a nonlinear Ramsey interferometer operating beyond the Heisenberg limit. Physical Review A.

[80] Napolitano M (2011). Interaction-based quantum metrology showing scaling beyond the Heisenberg limit. Nature (London).

[81] Joo J (2012). Quantum metrology for nonlinear phase shifts with entangled coherent states. Physical Review A.

[82] Kish SP, Ralph TC (2017). Quantum-limited measurement of space-time curvature with scaling beyond the conventional Heisenberg limit. Physical Review A.

[83] Tsarev DV, Arakelian SM, Chuang Y-L, Lee R-K, Alodjants AP (2018). Quantum metrology beyond Heisenberg limit with entangled matter wave solitons. Optics Express.

[84] Zwierz M, Pèrez-Delgado CA, Kok P (2010). General optimality of the Heisenberg limit for quantum metrology. Physical Review Letters.

[85] Pezzè L (2013). Sub-Heisenberg phase uncertainties. Physical Review A.

[86] Hall MJW, Wiseman HM (2012). Does nonlinear metrology offer improved resolution? Answers from quantum information theory. Physical Review X.

[87] Giovannetti V, Maccone L (2012). Sub-Heisenberg estimation strategies are ineffective. Physical Review Letters.

[88] Giovannetti V, Lloyd S, Maccone L (2012). Quantum measurement bounds beyond the uncertainty relations. Physical Review Letters.

[89] Hall MJW, Berry DW, Zwierz M, Wiseman HM (2012). Universality of the Heisenberg limit for estimates of random phase shifts. Physical Review A.

[90] Rams MM, Sierant P, Dutta O, Horodecki P, Zakrzewski J (2018). At the limits of criticality-based quantum metrology: Apparent super-Heisenberg scaling revisited. Physical Review X.

[91] Adesso G, Illuminati F, Siena SD (2003). Characterizing entanglement with global and marginal entropic measures. Physical Review A.

[92] Verstraete F, Audenaert K, Moor BD (2001). Maximally entangled mixed states of two qubits. Physical Review A.

[93] Li ZG, Zhao MJ, Fei SM, Fan H, Liu WM (2012). Mixed maximally entangled states. Quantum Information and Computation.

[94] Galve F, Giorgi GL, Zambrini R (2011). Maximally discordant mixed states of two qubits. Physical Review A.

[95] Modi K, Paterek T, Son W, Vedral V, Williamson M (2010). Unified view of quantum and classical correlations. Physical Review Letters.

[96] Robertson HP (1929). The uncertainty principle. Physical Review.

[97] Rigolin G (2016). Entanglement, identical particles and the uncertainty principle. Communications in Theoretical Physics.

[98] Werlang T, Souza S, Fanchini FF, Boas CJV (2009). Robustness of quantum discord to sudden death. Physical Review A.

[99] Almeida MP (2007). Environment-induced sudden death of entanglement. Science.

[100] Yu T, Eberly JH (2009). Sudden death of entanglement. Science.

[101] Luo S (2016). Entanglement as minimal discord over state extensions. Physical Review A.

[102] Streltsov A, Zurek WH (2013). Quantum discord cannot be shared. Physical Review Letters.

[103] Coecke, B. & Kissinger, A. *Picturing Quantum Processes* (Cambridge University Press, 2017).

[104] Nagaoka, H. A new approach to Cramér-Rao bounds for quantum state estimation. In *Asymptotic Theory of Quantum Statistical Inference*, Vol. 1, edited by Hayashi, M. Chap. 8 (World Scientific, 1989).

[105] Braunstein SL, Caves CM (1994). Statistical distance and the geometry of quantum states. Physical Review Letters.

[106] Hayashi, M. *Quantum Information - An Introduction* (Springer, 2006).

[107] Dittmann J (1999). Explicit formulae for the Bures metric. Journal of Physics A: Mathematical and General.

[108] Fawzi O, Renner R (2015). Quantum conditional mutual information and approximate Markov chains. Communications in Mathematical Physics.

[109] Dodd JL, Nielsen MA (2002). Simple operational interpretation of the fidelity of mixed states. Physical Review A.

[110] Petz, D. & Ghinea, C. Introduction to quantum Fisher information. In *Quantum Probability and Related Topics* pp. 261–281 (World Scientific, 2011).

