# Supplementary material for: Fundamental noisy multiparameter quantum bounds
Source: Sci Rep. 2019 Jan 31;9:1038. doi: 10.1038/s41598-018-37583-7 (PMC6355885; doi:10.1038/s41598-018-37583-7)
Supplement: Supplementary file 1 — Fundamental noisy multiparameter quantum bounds: Detailed Proofs [file 41598_2018_37583_MOESM1_ESM.pdf]

Supplementary Information

Fundamental noisy multiparameter quantum bounds

Shibdas Roy[1, *]

[1] *Department of Physics, University of Warwick, Coventry CV4 7AL, United Kingdom.*

(Dated: November 26, 2018)

I. PROOF FOR $\nu V\left[\tilde{\boldsymbol{\theta}}(m)\right] \geq \left[J_C(\boldsymbol{\theta})\right]^{-1} \geq \left[J_Q(\boldsymbol{\theta})\right]^{-1}$

Here, we prove the following quantum Cramér-Rao inequality, as claimed in the main text:

$$\nu V\left[\tilde{\boldsymbol{\theta}}(m)\right] \geq \left[J_C(\boldsymbol{\theta})\right]^{-1} \geq \left[J_Q(\boldsymbol{\theta})\right]^{-1}, \tag{1.1}$$

where ν is the number of times the experiment is repeated,

$$V\left[\tilde{\boldsymbol{\theta}}(m)\right] = \sum_m p(m|\boldsymbol{\theta})\left(\tilde{\boldsymbol{\theta}}(m) - \boldsymbol{\theta}\right)\left(\tilde{\boldsymbol{\theta}}(m) - \boldsymbol{\theta}\right)^T =: \Sigma \tag{1.2}$$

is the estimation error covariance,

$$J_C^{jk} = \sum_m \frac{1}{p(m|\boldsymbol{\theta})}\frac{\partial}{\partial\theta_j}p(m|\boldsymbol{\theta})\frac{\partial}{\partial\theta_k}p(m|\boldsymbol{\theta}) \tag{1.3}$$

is the classical Fisher information matrix (FIM), and

$$J_Q^{jk} = \frac{1}{2}\mathrm{Tr}\left[\left(\hat{L}_j^\dagger\hat{L}_k + \hat{L}_k^\dagger\hat{L}_j\right)\hat{\rho}(\boldsymbol{\theta})\right] \tag{1.4}$$

is the quantum Fisher information matrix (QFIM), with the operators $\hat{L}_k$ satisfying

$$\frac{1}{2}\left(\hat{L}_k\hat{\rho}(\boldsymbol{\theta}) + \hat{\rho}(\boldsymbol{\theta})\hat{L}_k^\dagger\right) = \frac{\partial}{\partial\theta_k}\hat{\rho}(\boldsymbol{\theta}). \tag{1.5}$$

The proof is adapted from Ref. [1] for frequentist multiparameter estimation problem here.

The estimates $\tilde{\boldsymbol{\theta}}(m) = \left[\begin{array}{cccc}\tilde{\theta}_1(m) & \tilde{\theta}_2(m) & \ldots & \tilde{\theta}_q(m)\end{array}\right]^T$ of the parameters $\boldsymbol{\theta} = \left[\begin{array}{cccc}\theta_1 & \theta_2 & \ldots & \theta_q\end{array}\right]^T$ are unbiased, if

$$\sum_m p(m|\boldsymbol{\theta})\tilde{\theta}_j(m) = \theta_j \qquad \forall j, \tag{1.6}$$

where $p(m|\boldsymbol{\theta}) = \mathrm{Tr}\left(\hat{P}_m\hat{\rho}(\boldsymbol{\theta})\right)$ is the conditional probability to obtain the outcome m from a measurement performed on the evolved probe state $\hat{\rho}(\boldsymbol{\theta})$ via a positive operator valued measure (POVM) $\{\hat{P}_m\}$, given that the parameters have the value $\boldsymbol{\theta}$. Differentiating (1.6) with respect to θ_k, we get

$$\delta_{jk} = \sum_m\left(\tilde{\theta}_j(m) - \theta_j\right)\frac{\partial p(m|\boldsymbol{\theta})}{\partial\theta_k} = \mathrm{Re}\sum_m\left(\tilde{\theta}_j(m) - \theta_j\right)\mathrm{Tr}\left[\hat{P}_m\hat{L}_k\hat{\rho}(\boldsymbol{\theta})\right]. \tag{1.7}$$

Then, following Ref. [1], since $\nu \geq 1$, we get:

$$\begin{aligned}\boldsymbol{v}^T\boldsymbol{u} &= \sum_j u_j v_j \leq A^T B,\\ \boldsymbol{w}^T\boldsymbol{u} &= \sum_k u_k w_k \leq \mathrm{Re}\left[\mathrm{Tr}\left(C^\dagger D\right)\right],\end{aligned} \tag{1.8}$$

* Email: roy_shibdas@yahoo.co.in

where $\boldsymbol{u}$, $\boldsymbol{v}$, $\boldsymbol{w}$ are arbitrary real column vectors, and

$$\begin{aligned}
A^T &= \sum_k v_k \frac{\partial p(m|\boldsymbol{\theta})}{\partial \theta_k} \frac{1}{\sqrt{p(m|\boldsymbol{\theta})}}, \\
B &= \sum_j u_j \left(\tilde{\theta}_j(m) - \theta_j \right) \sqrt{\nu} \sqrt{p(m|\boldsymbol{\theta})}, \\
C^\dagger &= \sum_l w_l \sqrt{\hat{P}_m} \hat{L}_l \sqrt{\hat{\rho}(\boldsymbol{\theta})}, \\
D &= \sum_j u_j \left(\tilde{\theta}_j(m) - \theta_j \right) \sqrt{\nu} \sqrt{\hat{\rho}(\boldsymbol{\theta})} \sqrt{\hat{P}_m}.
\end{aligned} \tag{1.9}$$

We assume that $\boldsymbol{v}^T\boldsymbol{u}$ and $\boldsymbol{w}^T\boldsymbol{u}$ are positive, which are valid assumptions given how we set these later. Then,

$$\begin{aligned}
\left(\boldsymbol{v}^T\boldsymbol{u}\right)^2 &\leq \left(A^T B\right)^2 \leq \left(A^T A\right)\left(B^T B\right), \\
\left(\boldsymbol{w}^T\boldsymbol{u}\right)^2 &\leq \left|\mathrm{Tr}\left(C^\dagger D\right)\right|^2 \leq \mathrm{Tr}\left(C^\dagger C\right)\mathrm{Tr}\left(D^\dagger D\right),
\end{aligned} \tag{1.10}$$

where the second inequalities in both lines are Schwarz inequalities.

Now, note that $A^T A = \boldsymbol{v}^T J_C \boldsymbol{v}$, where J_C is a real, symmetric and positive semidefinite classical Fisher information matrix (FIM) as defined in (1.3), $\mathrm{Tr}\left(C^\dagger C\right) = \boldsymbol{w}^T J_Q \boldsymbol{w}$, where J_Q is a real, symmetric and positive semidefinite quantum Fisher information matrix (QFIM) as defined in (1.4), and $B^T B = \mathrm{Tr}\left(D^\dagger D\right) = \boldsymbol{u}^T \nu\Sigma \boldsymbol{u}$, where $\nu\Sigma$ is the estimation error covariance matrix as defined in (1.2). Substituting these in (1.10), we find that

$$\begin{aligned}
\left(\boldsymbol{v}^T J_C \boldsymbol{v}\right)\left(\boldsymbol{u}^T \nu\Sigma \boldsymbol{u}\right) &\geq \left(\boldsymbol{v}^T\boldsymbol{u}\right)\left(\boldsymbol{u}^T\boldsymbol{v}\right), \\
\left(\boldsymbol{w}^T J_Q \boldsymbol{w}\right)\left(\boldsymbol{u}^T \nu\Sigma \boldsymbol{u}\right) &\geq \left(\boldsymbol{w}^T\boldsymbol{u}\right)\left(\boldsymbol{u}^T\boldsymbol{w}\right).
\end{aligned} \tag{1.11}$$

Setting $\boldsymbol{v} = J_C^{-1}\boldsymbol{u}$ implies that

$$\boldsymbol{u}^T\left(\nu\Sigma - J_C^{-1}\right)\boldsymbol{u} \geq 0, \tag{1.12}$$

for arbitrary real vectors $\boldsymbol{u}$. Since $\nu\Sigma - J_C^{-1}$ is real and symmetric, this implies that $\nu\Sigma - J_C^{-1}$ is positive semidefinite. Also, setting $\boldsymbol{w} = J_Q^{-1}\boldsymbol{u}$ implies that

$$\boldsymbol{u}^T\left(\nu\Sigma - J_Q^{-1}\right)\boldsymbol{u} \geq 0. \tag{1.13}$$

Since $\nu\Sigma - J_Q^{-1}$ is real and symmetric, this implies that $\nu\Sigma - J_Q^{-1}$ is positive semidefinite.

We now take $\boldsymbol{v} = \boldsymbol{w}$. Then, we have

$$\begin{aligned}
&\boldsymbol{v}^T\boldsymbol{u} \leq A^T B = \mathrm{Re}\left[\mathrm{Tr}\left(C^\dagger D\right)\right] \\
\Rightarrow &\left(\boldsymbol{v}^T\boldsymbol{u}\right)\left(\boldsymbol{u}^T\boldsymbol{v}\right) \leq \left|\mathrm{Tr}\left(C^\dagger D\right)\right|^2 \leq \mathrm{Tr}\left(C^\dagger C\right)\mathrm{Tr}\left(D^\dagger D\right) = \left(\boldsymbol{v}^T J_Q \boldsymbol{v}\right)\left(\boldsymbol{u}^T \nu\Sigma \boldsymbol{u}\right).
\end{aligned} \tag{1.14}$$

Then, again setting $\boldsymbol{v} = J_C^{-1}\boldsymbol{u}$ imply that

$$\left(\boldsymbol{u}^T J_C^{-1}\boldsymbol{u}\right)\left(\boldsymbol{u}^T J_C^{-1}\boldsymbol{u}\right) \leq \left(\boldsymbol{u}^T J_C^{-1} J_Q J_C^{-1}\boldsymbol{u}\right)\left(\boldsymbol{u}^T \nu\Sigma \boldsymbol{u}\right). \tag{1.15}$$

Now, since $\boldsymbol{u}^T\left(\nu\Sigma - J_C^{-1}\right)\boldsymbol{u} \geq 0$, we get from above

$$\begin{aligned}
&\boldsymbol{u}^T J_C^{-1}\boldsymbol{u} \leq \boldsymbol{u}^T J_C^{-1} J_Q J_C^{-1}\boldsymbol{u} \\
&\Rightarrow J_C^{-1} \leq J_C^{-1} J_Q J_C^{-1} \\
&\Rightarrow J_C^{-1} \geq J_Q^{-1}.
\end{aligned} \tag{1.16}$$

Thus, we have (1.1).

II. SATURABILITY OF ALD-BASED QCRB

Here, we prove that an ALD-based QCRB can be saturated when the expectation of the commutator of the ALDs vanishes, as claimed in the main text:

$$\mathrm{Tr}\left[\left(\hat{L}_j^\dagger \hat{L}_k - \hat{L}_k^\dagger \hat{L}_j\right)\hat{\rho}(\boldsymbol{\theta})\right] = \mathrm{Tr}\left(\left[\hat{L}_j, \hat{L}_k\right]\hat{\rho}(\boldsymbol{\theta})\right) = 0, \tag{2.1}$$

where the operators $\hat{L}_k$ are anti-Hermitian. The proof presented here is directly adapted from Ref. [2] for ALDs, and relies on the fact that it is enough to show that the QFIM bound is equivalent to the Holevo bound when (2.1) is satisfied, because the Holevo bound is a tighter bound, known to be asymptotically saturable.

Given that the operators $\hat{L}_k$ are anti-Hermitian and satisfy

$$\frac{1}{2}\left(\hat{L}_k\hat{\rho}(\boldsymbol{\theta}) - \hat{\rho}(\boldsymbol{\theta})\hat{L}_k\right) = \frac{\partial}{\partial\theta_k}\hat{\rho}(\boldsymbol{\theta}), \tag{2.2}$$

and the QFIM J_Q is given by (1.4), then (1.1) implies that for a given cost matrix G, the estimation cost is bounded by

$$\mathrm{tr}\left(G\nu V\left[\tilde{\boldsymbol{\theta}}(m)\right]\right) \geq \mathrm{tr}\left(GJ_Q^{-1}\right), \tag{2.3}$$

where tr denotes the trace of a matrix in distinction from Tr for an operator. Then, the achievable estimation uncertainty is lower-bounded by the Holevo Cramér-Rao bound [2, 3]:

$$\mathrm{tr}\left(G\nu V\left[\tilde{\boldsymbol{\theta}}(m)\right]\right) \geq \min_{\{\hat{X}_j\}}\left\{\mathrm{tr}\left(G\mathrm{Re}W\right) + ||G\mathrm{Im}W||_1\right\}, \tag{2.4}$$

where $||\cdot||_1$ is the operator trace norm, the elements of the matrix W are [3]

$$W_{jk} = \mathrm{Tr}\left(\hat{X}_j^\dagger\hat{X}_k\hat{\rho}(\boldsymbol{\theta})\right), \tag{2.5}$$

and the minimization is performed over the operators $\hat{X}_j$ satisfying

$$\frac{1}{2}\mathrm{Tr}\left[\left(\hat{X}_j^\dagger\hat{L}_k + \hat{L}_k^\dagger\hat{X}_j\right)\hat{\rho}(\boldsymbol{\theta})\right] = \delta_{jk}. \tag{2.6}$$

In our case, the operators $\hat{X}_j$ are also anti-Hermitian. The bound (2.4) is stronger than the bound (2.3), the right hand side of which can be rewritten in the form [2]:

$$\mathrm{tr}\left(GJ_Q^{-1}\right) = \min_{\{\hat{X}_j\}}\mathrm{tr}\left(G\mathrm{Re}W\right). \tag{2.7}$$

Then, the solution to the minimization problem in (2.7) is [2]

$$\hat{X}_j = \sum_k\left(G^{-1}\Lambda\right)_{jk}\hat{L}_k = \sum_k\left(J_Q^{-1}\right)_{jk}\hat{L}_k, \tag{2.8}$$

where Λ is a matrix of Lagrange multipliers, chosen so that $G^{-1}\Lambda J_Q = \mathbb{1}$.

Now, the cost matrix G and the QFIM J_Q are assumed to be strictly positive. Firstly, we assume that (2.1) holds for all j, k. We saw that the optimal $\hat{X}_j = \sum_k\left(J_Q^{-1}\right)_{jk}\hat{L}_k$ are linear combinations of $\hat{L}_j$. This implies that $\mathrm{Tr}\left(\left[\hat{X}_j, \hat{X}_k\right]\hat{\rho}(\boldsymbol{\theta})\right) = 0$ for all j, k. Hence, the same set of $\hat{X}_j$ minimizes the Holevo bound, since it makes the second term in (2.4) to equal zero. Thus, (2.1) is a sufficient condition for saturating the ALD-based QCRB corresponding to the QFIM (1.4).

Secondly, we assume that the Holevo bound coincides with the QFIM bound, and so for the $\hat{X}_j$ that minimize both (2.3) and (2.4), the second term in (2.4) must equal zero. Since G is strictly positive, the matrix ImW must be zero and hence $\mathrm{Tr}\left(\left[\hat{X}_j, \hat{X}_k\right]\hat{\rho}(\boldsymbol{\theta})\right) = 0$ for all j, k. However, the $\hat{X}_j$ that minimizes (2.3) is $\hat{X}_j = \sum_k\left(J_Q^{-1}\right)_{jk}\hat{L}_k$. Inverting this formula, we get $\hat{L}_j = \sum_k\left(\hat{J}_Q\right)_{jk}\hat{X}_k$. Hence, (2.1) holds for all j, k and is also a necessary condition for saturating the ALD-based QCRB corresponding to the QFIM (1.4).

III. THE STATES $\hat{\rho}^N$ ARE PERMUTATIONALLY INVARIANT

Here, we show that the first order and second order reduced density matrices are as claimed in the main text for the magnetic field example.

First, considering the $N = 2$ case:

$$
\begin{aligned}
\hat{\rho}_k^{N=2} = \frac{1}{2} \Big[& \hat{E}_0^{\otimes 2} |\phi_k^+, \phi_k^+\rangle\langle\phi_k^+, \phi_k^+| \hat{E}_0^{\otimes 2} + (\hat{E}_0 \otimes \hat{E}_1)|\phi_k^+, \phi_k^+\rangle\langle\phi_k^+, \phi_k^+|(\hat{E}_0 \otimes \hat{E}_1) \\
& + (\hat{E}_1 \otimes \hat{E}_0)|\phi_k^+, \phi_k^+\rangle\langle\phi_k^+, \phi_k^+|(\hat{E}_1 \otimes \hat{E}_0) + \hat{E}_1^{\otimes 2}|\phi_k^+, \phi_k^+\rangle\langle\phi_k^+, \phi_k^+|\hat{E}_1^{\otimes 2} \\
& + \hat{E}_0^{\otimes 2}|\phi_k^+, \phi_k^+\rangle\langle\phi_k^-, \phi_k^-|\hat{E}_0^{\otimes 2} + (\hat{E}_0 \otimes \hat{E}_1)|\phi_k^+, \phi_k^+\rangle\langle\phi_k^-, \phi_k^-|(\hat{E}_0 \otimes \hat{E}_1) \\
& + (\hat{E}_1 \otimes \hat{E}_0)|\phi_k^+, \phi_k^+\rangle\langle\phi_k^-, \phi_k^-|(\hat{E}_1 \otimes \hat{E}_0) + \hat{E}_1^{\otimes 2}|\phi_k^+, \phi_k^+\rangle\langle\phi_k^-, \phi_k^-|\hat{E}_1^{\otimes 2} \\
& + \hat{E}_0^{\otimes 2}|\phi_k^-, \phi_k^-\rangle\langle\phi_k^+, \phi_k^+|\hat{E}_0^{\otimes 2} + (\hat{E}_0 \otimes \hat{E}_1)|\phi_k^-, \phi_k^-\rangle\langle\phi_k^+, \phi_k^+|(\hat{E}_0 \otimes \hat{E}_1) \\
& + (\hat{E}_1 \otimes \hat{E}_0)|\phi_k^-, \phi_k^-\rangle\langle\phi_k^+, \phi_k^+|(\hat{E}_1 \otimes \hat{E}_0) + \hat{E}_1^{\otimes 2}|\phi_k^-, \phi_k^-\rangle\langle\phi_k^+, \phi_k^+|\hat{E}_1^{\otimes 2} \\
& + \hat{E}_0^{\otimes 2}|\phi_k^-, \phi_k^-\rangle\langle\phi_k^-, \phi_k^-|\hat{E}_0^{\otimes 2} + (\hat{E}_0 \otimes \hat{E}_1)|\phi_k^-, \phi_k^-\rangle\langle\phi_k^-, \phi_k^-|(\hat{E}_0 \otimes \hat{E}_1) \\
& + (\hat{E}_1 \otimes \hat{E}_0)|\phi_k^-, \phi_k^-\rangle\langle\phi_k^-, \phi_k^-|(\hat{E}_1 \otimes \hat{E}_0) + \hat{E}_1^{\otimes 2}|\phi_k^-, \phi_k^-\rangle\langle\phi_k^-, \phi_k^-|\hat{E}_1^{\otimes 2} \Big].
\end{aligned}
\tag{3.1}
$$

Then, tracing out the second qubit, we get:

$$
\begin{aligned}
\mathrm{Tr}_2\left[\rho_k^{N=2}\right] = & \frac{1}{2} \Big[\hat{E}_0|\phi_k^+\rangle\langle\phi_k^+|\hat{E}_0\langle\phi_k^+|\hat{E}_0^2|\phi_k^+\rangle + \hat{E}_0|\phi_k^+\rangle\langle\phi_k^+|\hat{E}_0\langle\phi_k^+|\hat{E}_1^2|\phi_k^+\rangle \\
& + \hat{E}_1|\phi_k^+\rangle\langle\phi_k^+|\hat{E}_1\langle\phi_k^+|\hat{E}_0^2|\phi_k^+\rangle + \hat{E}_1|\phi_k^+\rangle\langle\phi_k^+|\hat{E}_1\langle\phi_k^+|\hat{E}_1^2|\phi_k^+\rangle \\
& + \hat{E}_0|\phi_k^+\rangle\langle\phi_k^-|\hat{E}_0\langle\phi_k^+|\hat{E}_0^2|\phi_k^-\rangle + \hat{E}_0|\phi_k^+\rangle\langle\phi_k^-|\hat{E}_0\langle\phi_k^+|\hat{E}_1^2|\phi_k^-\rangle \\
& + \hat{E}_1|\phi_k^+\rangle\langle\phi_k^-|\hat{E}_1\langle\phi_k^+|\hat{E}_0^2|\phi_k^-\rangle + \hat{E}_1|\phi_k^+\rangle\langle\phi_k^-|\hat{E}_1\langle\phi_k^+|\hat{E}_1^2|\phi_k^-\rangle \\
& + \hat{E}_0|\phi_k^-\rangle\langle\phi_k^+|\hat{E}_0\langle\phi_k^-|\hat{E}_0^2|\phi_k^+\rangle + \hat{E}_0|\phi_k^-\rangle\langle\phi_k^+|\hat{E}_0\langle\phi_k^-|\hat{E}_1^2|\phi_k^+\rangle \\
& + \hat{E}_1|\phi_k^-\rangle\langle\phi_k^+|\hat{E}_1\langle\phi_k^-|\hat{E}_0^2|\phi_k^+\rangle + \hat{E}_1|\phi_k^-\rangle\langle\phi_k^+|\hat{E}_1\langle\phi_k^-|\hat{E}_1^2|\phi_k^+\rangle \\
& + \hat{E}_0|\phi_k^-\rangle\langle\phi_k^-|\hat{E}_0\langle\phi_k^-|\hat{E}_0^2|\phi_k^-\rangle + \hat{E}_0|\phi_k^-\rangle\langle\phi_k^-|\hat{E}_0\langle\phi_k^-|\hat{E}_1^2|\phi_k^-\rangle \\
& + \hat{E}_1|\phi_k^-\rangle\langle\phi_k^-|\hat{E}_1\langle\phi_k^-|\hat{E}_0^2|\phi_k^-\rangle + \hat{E}_1|\phi_k^-\rangle\langle\phi_k^-|\hat{E}_1\langle\phi_k^-|\hat{E}_1^2|\phi_k^-\rangle \Big] \\
= & \frac{1}{2} \Big[\hat{E}_0|\phi_k^+\rangle\langle\phi_k^+|\hat{E}_0\langle\phi_k^+|\hat{E}_0^2 + \hat{E}_1^2|\phi_k^+\rangle + \hat{E}_1|\phi_k^+\rangle\langle\phi_k^+|\hat{E}_1\langle\phi_k^+|\hat{E}_0^2 + \hat{E}_1^2|\phi_k^+\rangle \\
& + \hat{E}_0|\phi_k^+\rangle\langle\phi_k^-|\hat{E}_0\langle\phi_k^+|\hat{E}_0^2 + \hat{E}_1^2|\phi_k^-\rangle + \hat{E}_1|\phi_k^+\rangle\langle\phi_k^-|\hat{E}_1\langle\phi_k^+|\hat{E}_0^2 + \hat{E}_1^2|\phi_k^-\rangle \\
& + \hat{E}_0|\phi_k^-\rangle\langle\phi_k^+|\hat{E}_0\langle\phi_k^-|\hat{E}_0^2 + \hat{E}_1^2|\phi_k^+\rangle + \hat{E}_1|\phi_k^-\rangle\langle\phi_k^+|\hat{E}_1\langle\phi_k^-|\hat{E}_0^2 + \hat{E}_1^2|\phi_k^+\rangle \\
& + \hat{E}_0|\phi_k^-\rangle\langle\phi_k^-|\hat{E}_0\langle\phi_k^-|\hat{E}_0^2 + \hat{E}_1^2|\phi_k^-\rangle + \hat{E}_1|\phi_k^-\rangle\langle\phi_k^-|\hat{E}_1\langle\phi_k^-|\hat{E}_0^2 + \hat{E}_1^2|\phi_k^-\rangle \Big] \\
= & \frac{1}{2} \Big[\hat{E}_0|\phi_k^+\rangle\langle\phi_k^+|\hat{E}_0\langle\phi_k^+|\phi_k^+\rangle + \hat{E}_1|\phi_k^+\rangle\langle\phi_k^+|\hat{E}_1\langle\phi_k^+|\phi_k^+\rangle \\
& + \hat{E}_0|\phi_k^+\rangle\langle\phi_k^-|\hat{E}_0\langle\phi_k^+|\phi_k^-\rangle + \hat{E}_1|\phi_k^+\rangle\langle\phi_k^-|\hat{E}_1\langle\phi_k^+|\phi_k^-\rangle \\
& + \hat{E}_0|\phi_k^-\rangle\langle\phi_k^+|\hat{E}_0\langle\phi_k^-|\phi_k^+\rangle + \hat{E}_1|\phi_k^-\rangle\langle\phi_k^+|\hat{E}_1\langle\phi_k^-|\phi_k^+\rangle \\
& + \hat{E}_0|\phi_k^-\rangle\langle\phi_k^-|\hat{E}_0\langle\phi_k^-|\phi_k^-\rangle + \hat{E}_1|\phi_k^-\rangle\langle\phi_k^-|\hat{E}_1\langle\phi_k^-|\phi_k^-\rangle \Big] \\
= & \frac{1}{2} \Big[\hat{E}_0|\phi_k^+\rangle\langle\phi_k^+|\hat{E}_0 + \hat{E}_1|\phi_k^+\rangle\langle\phi_k^+|\hat{E}_1 + \hat{E}_0|\phi_k^-\rangle\langle\phi_k^-|\hat{E}_0 + \hat{E}_1|\phi_k^-\rangle\langle\phi_k^-|\hat{E}_1 \Big] \\
= & \frac{1}{2} \left[\sum_{r=0}^{1} \hat{E}_r \left(|\phi_k^+\rangle\langle\phi_k^+| + |\phi_k^-\rangle\langle\phi_k^-| \right) \hat{E}_r \right] = \frac{\mathbb{1}_2}{2}.
\end{aligned}
\tag{3.2}
$$

Similarly, considering the $N=3$ case, and then tracing out the third qubit, we get:

$$\begin{aligned}
\mathrm{Tr}_3\left[\hat{\rho}_k^{N=3}\right] =&\frac{1}{2}\Big[\hat{E}_0^{\otimes 2}|\phi_k^+,\phi_k^+\rangle\langle\phi_k^+,\phi_k^+|\hat{E}_0^{\otimes 2}+(\hat{E}_0\otimes\hat{E}_1)|\phi_k^+,\phi_k^+\rangle\langle\phi_k^+,\phi_k^+|(\hat{E}_0\otimes\hat{E}_1)\\
&+(\hat{E}_1\otimes\hat{E}_0)|\phi_k^+,\phi_k^+\rangle\langle\phi_k^+,\phi_k^+|(\hat{E}_1\otimes\hat{E}_0)+\hat{E}_1^{\otimes 2}|\phi_k^+,\phi_k^+\rangle\langle\phi_k^+,\phi_k^+|\hat{E}_1^{\otimes 2}\\
&+\hat{E}_0^{\otimes 2}|\phi_k^-,\phi_k^-\rangle\langle\phi_k^-,\phi_k^-|\hat{E}_0^{\otimes 2}+(\hat{E}_0\otimes\hat{E}_1)|\phi_k^-,\phi_k^-\rangle\langle\phi_k^-,\phi_k^-|(\hat{E}_0\otimes\hat{E}_1)\\
&+(\hat{E}_1\otimes\hat{E}_0)|\phi_k^-,\phi_k^-\rangle\langle\phi_k^-,\phi_k^-|(\hat{E}_1\otimes\hat{E}_0)+\hat{E}_1^{\otimes 2}|\phi_k^-,\phi_k^-\rangle\langle\phi_k^-,\phi_k^-|\hat{E}_1^{\otimes 2}\Big]\\
=&\frac{1}{2}\Big[\hat{E}_0^{\otimes 2}\left(|\phi_k^+,\phi_k^+\rangle\langle\phi_k^+,\phi_k^+|+|\phi_k^-,\phi_k^-\rangle\langle\phi_k^-,\phi_k^-|\right)\hat{E}_0^{\otimes 2}\\
&+(\hat{E}_0\otimes\hat{E}_1)\left(|\phi_k^+,\phi_k^+\rangle\langle\phi_k^+,\phi_k^+|+|\phi_k^-,\phi_k^-\rangle\langle\phi_k^-,\phi_k^-|\right)(\hat{E}_0\otimes\hat{E}_1)\\
&+(\hat{E}_1\otimes\hat{E}_0)\left(|\phi_k^+,\phi_k^+\rangle\langle\phi_k^+,\phi_k^+|+|\phi_k^-,\phi_k^-\rangle\langle\phi_k^-,\phi_k^-|\right)(\hat{E}_1\otimes\hat{E}_0)\\
&+\hat{E}_1^{\otimes 2}\left(|\phi_k^+,\phi_k^+\rangle\langle\phi_k^+,\phi_k^+|+|\phi_k^-,\phi_k^-\rangle\langle\phi_k^-,\phi_k^-|\right)\hat{E}_1^{\otimes 2}\Big]\\
=&\frac{1}{4}\Big[\mathbb{1}_2\otimes\mathbb{1}_2+\hat{E}_0^{\otimes 2}\left(\hat{\sigma}_k\otimes\hat{\sigma}_k\right)\hat{E}_0^{\otimes 2}+(\hat{E}_0\otimes\hat{E}_1)\left(\hat{\sigma}_k\otimes\hat{\sigma}_k\right)(\hat{E}_0\otimes\hat{E}_1)\\
&+(\hat{E}_1\otimes\hat{E}_0)\left(\hat{\sigma}_k\otimes\hat{\sigma}_k\right)(\hat{E}_1\otimes\hat{E}_0)+\hat{E}_1^{\otimes 2}\left(\hat{\sigma}_k\otimes\hat{\sigma}_k\right)\hat{E}_1^{\otimes 2}\Big]\\
=&\frac{1}{4}\left[\mathbb{1}_2\otimes\mathbb{1}_2+\left(\sum_{r=0}^{1}\hat{E}_r\hat{\sigma}_k\hat{E}_r\right)\otimes\left(\sum_{s=0}^{1}\hat{E}_s\hat{\sigma}_k\hat{E}_s\right)\right],
\end{aligned} \tag{3.3}$$

and so on.

IV. PROOF FOR $C_Q(\boldsymbol{\theta}) \geq J_Q(\boldsymbol{\theta})$

Here, we prove that the quantity $C_Q(\boldsymbol{\theta})$ is indeed an upper bound to the quantity $J_Q(\boldsymbol{\theta})$ for the evolved probe state $\hat{\rho}(\boldsymbol{\theta})$, as claimed in the main text. Consider the following relationship of the Bures fidelity with the quantum Fisher information matrix (QFIM), where the QFIM is real, symmetric and positive semidefinite but more general and not necessarily composed of symmetric logarithmic derivatives (SLDs):

$$F\left(\hat{\rho}(\boldsymbol{\theta}),\hat{\rho}(\boldsymbol{\theta}+\boldsymbol{\epsilon})\right)=1-\frac{1}{4}\sum_{j,k}\epsilon_j\epsilon_k\mathrm{Tr}\left[\frac{\hat{L}_j^\dagger\hat{L}_k+\hat{L}_k^\dagger\hat{L}_j}{2}\hat{\rho}(\boldsymbol{\theta})\right], \tag{4.1}$$

where $\boldsymbol{\theta}$ is assumed to be the actual value of the vector of unknown parameters, $\boldsymbol{\epsilon}$ is an infinitesimal increment in $\boldsymbol{\theta}$, and $0\leq F(\hat{\rho}_1,\hat{\rho}_2)=\mathrm{Tr}\left(\sqrt{\sqrt{\hat{\rho}_1}\hat{\rho}_2\sqrt{\hat{\rho}_1}}\right)\leq 1$ is the Bures fidelity between two given states $\hat{\rho}_1$ and $\hat{\rho}_2$ [4–9]. Here, (4.1) holds, when the operators $\hat{L}_k$ are not necessarily Hermitian and satisfy:

$$\frac{1}{2}\left(\hat{L}_k\hat{\rho}(\boldsymbol{\theta})+\hat{\rho}(\boldsymbol{\theta})\hat{L}_k^\dagger\right)=\frac{\partial\hat{\rho}(\boldsymbol{\theta})}{\partial\theta_k}. \tag{4.2}$$

This can be seen as follows. When the operators $\hat{L}_k$ are Hermitian, such that $\hat{L}_k^\dagger=\hat{L}_k$, as is the convention, the Bures metric d_B and Bures distance D_B are defined and related to the fidelity F for infinitesimal $\boldsymbol{\epsilon}$ as follows [4, 10]:

$$d_B^2\left(\hat{\rho}(\boldsymbol{\theta}),\hat{\rho}(\boldsymbol{\theta}+\boldsymbol{\epsilon})\right)=D_B^2\left(\hat{\rho}(\boldsymbol{\theta}),\hat{\rho}(\boldsymbol{\theta}+\boldsymbol{\epsilon})\right)=2\left[1-F\left(\hat{\rho}(\boldsymbol{\theta}),\hat{\rho}(\boldsymbol{\theta}+\boldsymbol{\epsilon})\right)\right]=\frac{1}{2}\sum_{j,k}\epsilon_j\epsilon_k\mathrm{Tr}\left[\frac{\hat{L}_j\hat{L}_k+\hat{L}_k\hat{L}_j}{2}\hat{\rho}(\boldsymbol{\theta})\right], \tag{4.3}$$

where $\hat{L}_k$ are the SLDs satisfying:

$$\frac{1}{2}\left(\hat{L}_k\hat{\rho}(\boldsymbol{\theta})+\hat{\rho}(\boldsymbol{\theta})\hat{L}_k\right)=\frac{\partial\hat{\rho}(\boldsymbol{\theta})}{\partial\theta_k}. \tag{4.4}$$

However, if the operators $\hat{L}_k$ are not necessarily Hermitian and rather satisfy (4.2), then (4.3) becomes:

$$d_B^2\left(\hat{\rho}(\boldsymbol{\theta}),\hat{\rho}(\boldsymbol{\theta}+\boldsymbol{\epsilon})\right)=D_B^2\left(\hat{\rho}(\boldsymbol{\theta}),\hat{\rho}(\boldsymbol{\theta}+\boldsymbol{\epsilon})\right)=2\left[1-F\left(\hat{\rho}(\boldsymbol{\theta}),\hat{\rho}(\boldsymbol{\theta}+\boldsymbol{\epsilon})\right)\right]=\frac{1}{2}\sum_{j,k}\epsilon_j\epsilon_k\mathrm{Tr}\left[\frac{\hat{L}_j^\dagger\hat{L}_k+\hat{L}_k^\dagger\hat{L}_j}{2}\hat{\rho}(\boldsymbol{\theta})\right]. \tag{4.5}$$

Then, clearly (4.1) is obtained from the above.

We must comment here that there is a lot of inconsistency in the literature about the relationship between d_B, D_B and F. We here used the relationship originally presented in Ref. [4].

Now, for our case in this paper, the operators $\hat{L}_k$ are anti-symmetric logarithmic derivatives (ALDs), such that $\hat{L}_k^\dagger = -\hat{L}_k$. We have from (4.1):

$$F\left(\hat{\rho}(\boldsymbol{\theta}), \hat{\rho}(\boldsymbol{\theta}+\boldsymbol{\epsilon})\right) = 1 - \frac{1}{4}\sum_{j,k} \epsilon_j \epsilon_k J_Q^{jk}(\boldsymbol{\theta}). \tag{4.6}$$

Now, since fidelity is non-decreasing with respect to partial trace (See Refs. [5, 8, 11, 12], for example), we have:

$$F\left(\hat{\rho}(\boldsymbol{\theta}), \hat{\rho}(\boldsymbol{\theta}+\boldsymbol{\epsilon})\right) = F\left(\mathrm{Tr}_B\left[\hat{\rho}_{SB}(\boldsymbol{\theta})\right], \mathrm{Tr}_B\left[\hat{\rho}_{SB}(\boldsymbol{\theta}+\boldsymbol{\epsilon})\right]\right) \geq F\left(\hat{\rho}_{SB}(\boldsymbol{\theta}), \hat{\rho}_{SB}(\boldsymbol{\theta}+\boldsymbol{\epsilon})\right) = 1 - \frac{1}{4}\sum_{j,k} \epsilon_j \epsilon_k C_Q^{jk}(\boldsymbol{\theta}). \tag{4.7}$$

Clearly, from (4.6) and (4.7), we have (like in Ref. [7]):

$$C_Q(\boldsymbol{\theta}) \geq J_Q(\boldsymbol{\theta}). \tag{4.8}$$

An alternative argument for (4.8) to hold is that the quantum Fisher information (for both single and multiparamter cases) is an operator monotone function, non-increasing with respect to partial trace [13, 14], noting that the partial trace is a completely positive and trace-preserving map from $S+B$ space to S space.

Note that, even though we did not explicitly invoke Uhlmann's theorem here, the inequality in (4.7) is the monotonicity property of fidelity and is a consequence of Uhlmann's theorem. Thus, extending the argument from Ref. [15] to the multiparameter case, the equality in (4.8) is achieved by minimizing $C_Q(\boldsymbol{\theta})$ over all Kraus representations of the quantum channel. Hence, there are an infinitude of Kraus representations of the channel that lead to $C_Q(\boldsymbol{\theta}) = J_Q(\boldsymbol{\theta})$.

V. POVM TO ATTAIN QCRB FOR PURE STATE INPUT VIA UNITARY CHANNEL

Here, we prove that, as claimed in the main text, the set of POVMs $\{\hat{P}_{m1}\}$ of cardinality $q+2$, comprising the following $q+1$ elements,

$$\hat{P}_0 = \hat{\rho}(\boldsymbol{\theta}) = \hat{U}(\boldsymbol{\theta})|\psi\rangle\langle\psi|\hat{U}^\dagger(\boldsymbol{\theta}), \qquad \hat{P}_m = \frac{\partial \hat{U}(\boldsymbol{\theta})}{\partial \theta_m}|\psi\rangle\langle\psi|\frac{\partial \hat{U}^\dagger(\boldsymbol{\theta})}{\partial \theta_m} \qquad \forall m = 1, \ldots, q, \tag{5.1}$$

together with one element $\hat{P}_n = \hat{P}_{q+1} := |\phi_n\rangle\langle\phi_n|$ that accounts for the normalisation, saturates the ALD-based QCRB, provided (2.1) is satisfied for every pair of ALDs.

The proof is adapted from Ref. [16], noting that for pure state and unitary channel our ALD-based QCRB coincides with the SLD-based QCRB, and it is enough to demonstrate that using the set of POVMs $\{\hat{P}_{m1}\}$ the quantum Fisher information matrix (QFIM) equals the classical Fisher information matrix (FIM), when (2.1) is satisfied. The set of POVMs must be complete, i.e. $\sum_{m1} \hat{P}_{m1} = \mathbb{1}$.

Consider that the initial probe state is $\hat{\rho} = |\psi\rangle\langle\psi|$. Then, we use the short notations

$$|\psi_{\boldsymbol{\theta}}\rangle = \hat{U}(\boldsymbol{\theta})|\psi\rangle, \qquad |\partial_{\theta_k}\psi_{\boldsymbol{\theta}}\rangle = \frac{\partial}{\partial \theta_k}|\psi_{\boldsymbol{\theta}}\rangle = \frac{\partial \hat{U}(\boldsymbol{\theta})}{\partial \theta_k}|\psi\rangle. \tag{5.2}$$

The elements of the quantum Fisher information matrix (QFIM) are given by [16, 17]

$$J_Q^{jk} = 4\mathrm{Re}\left[\langle \partial_{\theta_j}\psi_{\boldsymbol{\theta}}|\partial_{\theta_k}\psi_{\boldsymbol{\theta}}\rangle - \langle \partial_{\theta_j}\psi_{\boldsymbol{\theta}}|\psi_{\boldsymbol{\theta}}\rangle\langle\psi_{\boldsymbol{\theta}}|\partial_{\theta_k}\psi_{\boldsymbol{\theta}}\rangle\right]. \tag{5.3}$$

The elements of the corresponding classical Fisher information matrix (FIM) J_C are given by [16]

$$J_C^{jk} = \sum_{m=0}^{q+1} \frac{\partial_{\theta_j} p(m|\boldsymbol{\theta}) \partial_{\theta_k} p(m|\boldsymbol{\theta})}{p(m|\boldsymbol{\theta})} = \sum_m \frac{4\mathrm{Re}\left[\langle \partial_{\theta_j}\psi_{\boldsymbol{\theta}}|\hat{P}_m|\psi_{\boldsymbol{\theta}}\rangle\right] \mathrm{Re}\left[\langle\psi_{\boldsymbol{\theta}}|\hat{P}_m|\partial_{\theta_k}\psi_{\boldsymbol{\theta}}\rangle\right]}{\langle\psi_{\boldsymbol{\theta}}|\hat{P}_m|\psi_{\boldsymbol{\theta}}\rangle}. \tag{5.4}$$

The component of the FIM corresponding to the POVM element $\hat{P}_0 = |\psi_{\boldsymbol{\theta}}\rangle\langle\psi_{\boldsymbol{\theta}}|$ is

$$4\mathrm{Re}\left[\langle \partial_{\theta_j}\psi_{\boldsymbol{\theta}}|\psi_{\boldsymbol{\theta}}\rangle\right] \mathrm{Re}\left[\langle\psi_{\boldsymbol{\theta}}|\partial_{\theta_k}\psi_{\boldsymbol{\theta}}\rangle\right] = 0. \tag{5.5}$$

The above quantity vanishes because $\mathrm{Re}\left[\langle\partial_{\theta_j}\psi_{\boldsymbol{\theta}}|\psi_{\boldsymbol{\theta}}\rangle\right]=0$ for any parameter θ_k [16, 18].

Next, the component of the FIM corresponding to the POVM element $\hat{P}_n=\hat{P}_{q+1}=|\phi_n\rangle\langle\phi_n|$ is

$$\frac{4\mathrm{Re}\left[\langle\partial_{\theta_j}\psi_{\boldsymbol{\theta}}|\hat{P}_n|\psi_{\boldsymbol{\theta}}\rangle\right]\mathrm{Re}\left[\langle\psi_{\boldsymbol{\theta}}|\hat{P}_n|\partial_{\theta_k}\psi_{\boldsymbol{\theta}}\rangle\right]}{\langle\psi_{\boldsymbol{\theta}}|\hat{P}_n|\psi_{\boldsymbol{\theta}}\rangle}=4\mathrm{Re}\left[\langle\partial_{\theta_j}\psi_{\boldsymbol{\theta}}|\phi_n\rangle\langle\phi_n|\partial_{\theta_k}\psi_{\boldsymbol{\theta}}\rangle\right], \tag{5.6}$$

since $\langle\psi_{\boldsymbol{\theta}}|\hat{P}_n|\psi_{\boldsymbol{\theta}}\rangle$ is, by definition, real.

The remaining components $\hat{P}_k$ for $k=1,\ldots,q$ may be similarly computed, and we get

$$J_C^{jk}=4\sum_{m=1}^{q}\mathrm{Re}\left[\langle\partial_{\theta_j}\psi_{\boldsymbol{\theta}}|\partial_{\theta_m}\psi_{\boldsymbol{\theta}}\rangle\langle\partial_{\theta_m}\psi_{\boldsymbol{\theta}}|\partial_{\theta_k}\psi_{\boldsymbol{\theta}}\rangle\right]+4\mathrm{Re}\left[\langle\partial_{\theta_j}\psi_{\boldsymbol{\theta}}|\phi_n\rangle\langle\phi_n|\partial_{\theta_k}\psi_{\boldsymbol{\theta}}\rangle\right]. \tag{5.7}$$

Now, note that, for the completeness of the set of POVMs, we require

$$\sum_{m=1}^{q}|\partial_{\theta_m}\psi_{\boldsymbol{\theta}}\rangle\langle\partial_{\theta_m}\psi_{\boldsymbol{\theta}}|+|\phi_n\rangle\langle\phi_n|=\mathbb{1}-|\psi_{\boldsymbol{\theta}}\rangle\langle\psi_{\boldsymbol{\theta}}|. \tag{5.8}$$

Substituting (5.8) in (5.7), we get

$$J_C^{jk}=4\mathrm{Re}\left[\langle\partial_{\theta_j}\psi_{\boldsymbol{\theta}}|\partial_{\theta_k}\psi_{\boldsymbol{\theta}}\rangle-\langle\partial_{\theta_j}\psi_{\boldsymbol{\theta}}|\psi_{\boldsymbol{\theta}}\rangle\langle\psi_{\boldsymbol{\theta}}|\partial_{\theta_k}\psi_{\boldsymbol{\theta}}\rangle\right]=J_Q^{jk}. \tag{5.9}$$

VI. POVM TO ATTAIN QCRB FOR MIXED STATE INPUT VIA UNITARY CHANNEL

Here, we prove that, as claimed in the main text, the set of POVMs $\{\hat{P}_{m2}\}$ of cardinality $q+2$, comprising the following $q+1$ elements,

$$\hat{P}_0=\hat{\rho}(\boldsymbol{\theta})=\hat{U}(\boldsymbol{\theta})\hat{\rho}\hat{U}^\dagger(\boldsymbol{\theta}),\qquad \hat{P}_m=\frac{\partial\hat{\rho}(\boldsymbol{\theta})}{\partial\theta_m}=\left[\frac{\partial\hat{U}(\boldsymbol{\theta})}{\partial\theta_m}\hat{\rho}\hat{U}^\dagger(\boldsymbol{\theta})+\hat{U}(\boldsymbol{\theta})\hat{\rho}\frac{\partial\hat{U}^\dagger(\boldsymbol{\theta})}{\partial\theta_m}\right]\quad\forall m=1,\ldots,q, \tag{6.1}$$

together with one element $\hat{P}_n=\hat{P}_{q+1}$ that accounts for the normalisation, saturates the ALD-based QCRB, provided (2.1) is satisfied for every pair of ALDs.

The elements of the QFIM with $\hat{\rho}_{\boldsymbol{\theta}}:=\hat{\rho}(\boldsymbol{\theta})$ and $\hat{U}_{\boldsymbol{\theta}}:=\hat{U}(\boldsymbol{\theta})$ are:

$$J_Q^{jk}=\frac{1}{2}\mathrm{Tr}\left[\left(\hat{L}_j^\dagger\hat{L}_k+\hat{L}_k^\dagger\hat{L}_j\right)\hat{\rho}_{\boldsymbol{\theta}}\right]=4\mathrm{Re}\left[\mathrm{Tr}\left(\hat{U}_{\boldsymbol{\theta}}\partial_{\theta_j}\hat{U}_{\boldsymbol{\theta}}^\dagger\partial_{\theta_k}\hat{U}_{\boldsymbol{\theta}}\hat{U}_{\boldsymbol{\theta}}^\dagger\hat{\rho}_{\boldsymbol{\theta}}\right)+\mathrm{Tr}\left(\hat{U}_{\boldsymbol{\theta}}\partial_{\theta_j}\hat{U}_{\boldsymbol{\theta}}^\dagger\hat{\rho}_{\boldsymbol{\theta}}\right)\mathrm{Tr}\left(\hat{U}_{\boldsymbol{\theta}}\partial_{\theta_k}\hat{U}_{\boldsymbol{\theta}}^\dagger\hat{\rho}_{\boldsymbol{\theta}}\right)\right], \tag{6.2}$$

where we used $\hat{L}_k=2\left[\partial_{\theta_k}\hat{U}_{\boldsymbol{\theta}}\hat{U}_{\boldsymbol{\theta}}^\dagger-\mathrm{Tr}\left(\partial_{\theta_k}\hat{U}_{\boldsymbol{\theta}}\hat{U}_{\boldsymbol{\theta}}^\dagger\hat{\rho}_{\boldsymbol{\theta}}\right)\right]$ (as taken in the main text), that satisfy:

$$2\partial_{\theta_k}\hat{\rho}_{\boldsymbol{\theta}}=\hat{L}_k\hat{\rho}_{\boldsymbol{\theta}}+\hat{\rho}_{\boldsymbol{\theta}}\hat{L}_k^\dagger,\qquad \hat{L}_k^\dagger=-\hat{L}_k, \tag{6.3}$$

noting that $\hat{U}_{\boldsymbol{\theta}}\partial_{\theta_k}\hat{U}_{\boldsymbol{\theta}}^\dagger=-\partial_{\theta_k}\hat{U}_{\boldsymbol{\theta}}\hat{U}_{\boldsymbol{\theta}}^\dagger$, arising from $\hat{U}_{\boldsymbol{\theta}}\hat{U}_{\boldsymbol{\theta}}^\dagger=\mathbb{1}$ upon differentiating both sides with respect to θ_k.

Also, the elements of the FIM J_C, as defined in (1.3), are:

$$J_C^{jk}=\sum_m\frac{1}{p(m|\boldsymbol{\theta})}\frac{\partial}{\partial\theta_j}p(m|\boldsymbol{\theta})\frac{\partial}{\partial\theta_k}p(m|\boldsymbol{\theta})=\sum_m\frac{1}{\mathrm{Tr}\left(\hat{P}_m\hat{\rho}_{\boldsymbol{\theta}}\right)}\frac{\partial}{\partial\theta_j}\mathrm{Tr}\left(\hat{P}_m\hat{\rho}_{\boldsymbol{\theta}}\right)\frac{\partial}{\partial\theta_k}\mathrm{Tr}\left(\hat{P}_m\hat{\rho}_{\boldsymbol{\theta}}\right). \tag{6.4}$$

Consider that we are interested in saturating the bound at a specific point θ_s in the space of $\boldsymbol{\theta}$, as in Ref. [16]. Then, (5.5) here becomes:

$$\frac{\mathrm{Tr}\left(\partial_{\theta_j}\hat{\rho}_{\boldsymbol{\theta}s}\hat{\rho}_{\boldsymbol{\theta}s}\right)\mathrm{Tr}\left(\partial_{\theta_k}\hat{\rho}_{\boldsymbol{\theta}s}\hat{\rho}_{\boldsymbol{\theta}s}\right)}{\mathrm{Tr}\left(\hat{\rho}_{\boldsymbol{\theta}s}^2\right)}=0, \tag{6.5}$$

where $\mathrm{Tr}\left(\partial_{\theta_j}\hat{\rho}_{\boldsymbol{\theta}s}\hat{\rho}_{\boldsymbol{\theta}s}\right)=0$ for any parameter θ_k, as an extension of Refs. [16, 18]. This can be seen as follows. Given that $\hat{\rho}_{\boldsymbol{\theta}s}$ is not necessarily pure, we must have $\mathrm{Tr}\left(\partial_{\theta_j}\hat{\rho}_{\boldsymbol{\theta}s}\hat{\rho}_{\boldsymbol{\theta}s}\right)\leq 0$, arising upon differentiation with respect to θ_j from $\mathrm{Tr}\left(\hat{\rho}_{\boldsymbol{\theta}s}^2\right)\leq 1$, for which $\mathrm{Tr}\left(\hat{\rho}_{\boldsymbol{\theta}s}^2\right)$ is clearly non-decreasing. However, since $\partial_{\theta_j}\hat{\rho}_{\boldsymbol{\theta}s}$ is a POVM element, we must have $\mathrm{Tr}\left(\partial_{\theta_j}\hat{\rho}_{\boldsymbol{\theta}s}\hat{\rho}_{\boldsymbol{\theta}s}\right)=\langle\partial_{\theta_j}\hat{\rho}_{\boldsymbol{\theta}s}\rangle=p(j|\theta_s)$, which being a probability cannot be negative. Here, $\langle\cdot\rangle$ denotes expectation

with respect to $\hat{\rho}_{\boldsymbol{\theta s}}$. Hence, we must have $\mathrm{Tr}\left(\partial_{\theta_j}\hat{\rho}_{\boldsymbol{\theta s}}\hat{\rho}_{\boldsymbol{\theta s}}\right)=0$. For example, when the state $\hat{\rho}_{\boldsymbol{\theta s}}$ is maximally mixed, i.e. $\hat{\rho}_{\boldsymbol{\theta s}}=\mathbb{1}_d/d$, where d is the dimension of the Hilbert space upon which the state $\hat{\rho}_{\boldsymbol{\theta s}}$ is defined, we have $\mathrm{Tr}\left(\hat{\rho}^2_{\boldsymbol{\theta s}}\right)=1/d$, and consequently, $\mathrm{Tr}\left(\partial_{\theta_j}\hat{\rho}_{\boldsymbol{\theta s}}\hat{\rho}_{\boldsymbol{\theta s}}\right)=0$. On the other hand, if $\hat{\rho}_{\boldsymbol{\theta s}}$ is pure, we must have $\mathrm{Tr}\left(\hat{\rho}^2_{\boldsymbol{\theta s}}\right)=1$, and consequently, $\mathrm{Tr}\left(\partial_{\theta_j}\hat{\rho}_{\boldsymbol{\theta s}}\hat{\rho}_{\boldsymbol{\theta s}}\right)=0$ again.

Next, proceeding in a manner similar to Ref. [16] for the terms of the FIM for $m=1,\dots,q$, we take $\hat{\rho}_{\boldsymbol{\theta}}=\hat{\rho}_{\boldsymbol{\theta s}}+\delta\theta_r\partial_{\theta_r}\hat{\rho}_{\boldsymbol{\theta s}}$. Clearly, $\mathrm{Tr}\left(\partial_{\theta_j}\hat{\rho}_{\boldsymbol{\theta s}}\partial_{\theta_m}\hat{\rho}_{\boldsymbol{\theta s}}\right)=0$ (even for $j=m$), arising from $\mathrm{Tr}\left(\partial_{\theta_j}\hat{\rho}_{\boldsymbol{\theta s}}\hat{\rho}_{\boldsymbol{\theta s}}\right)=0$ upon differentiating both sides with respect to θ_m, and noting that $\mathrm{Tr}\left(\partial_{\theta_j}\partial_{\theta_m}\hat{\rho}_{\boldsymbol{\theta s}}\hat{\rho}_{\boldsymbol{\theta s}}\right)=\langle\partial_{\theta_j}\partial_{\theta_m}\hat{\rho}_{\boldsymbol{\theta s}}\rangle=0$, since $\langle\partial_{\theta_j}\hat{\rho}_{\boldsymbol{\theta s}}\rangle=0$. In general, we must have $\mathrm{Tr}\left(\partial_{\theta_j}\hat{\rho}_{\boldsymbol{\theta s}}\hat{P}_m\right)=0$, $\forall m=0,1,\dots,q+1$. Thus, we have $\mathrm{Tr}\left(\partial_{\theta_j}\hat{\rho}_{\boldsymbol{\theta s}}\partial_{\theta_m}\hat{\rho}_{\boldsymbol{\theta}}\right)=\delta\theta_r\mathrm{Tr}\left(\partial_{\theta_j}\hat{\rho}_{\boldsymbol{\theta s}}\partial_{\theta_m}\partial_{\theta_r}\hat{\rho}_{\boldsymbol{\theta s}}\right)$, $\mathrm{Tr}\left(\partial_{\theta_m}\hat{\rho}_{\boldsymbol{\theta}}\partial_{\theta_k}\hat{\rho}_{\boldsymbol{\theta s}}\right)=\delta\theta_r\mathrm{Tr}\left(\partial_{\theta_m}\partial_{\theta_r}\hat{\rho}_{\boldsymbol{\theta s}}\partial_{\theta_k}\hat{\rho}_{\boldsymbol{\theta s}}\right)$, and $\mathrm{Tr}\left(\partial_{\theta_m}\hat{\rho}_{\boldsymbol{\theta}}\hat{\rho}_{\boldsymbol{\theta}}\right)=\delta\theta_r^2\mathrm{Tr}\left(\partial_{\theta_m}\partial_{\theta_r}\hat{\rho}_{\boldsymbol{\theta s}}\partial_{\theta_r}\hat{\rho}_{\boldsymbol{\theta s}}\right)$. Then, we get

$$\begin{aligned}\sum_{m=1}^{q}\frac{\delta\theta_r^2\mathrm{Tr}\left(\partial_{\theta_j}\hat{\rho}_{\boldsymbol{\theta s}}\partial_{\theta_m}\partial_{\theta_r}\hat{\rho}_{\boldsymbol{\theta s}}\right)\mathrm{Tr}\left(\partial_{\theta_m}\partial_{\theta_r}\hat{\rho}_{\boldsymbol{\theta s}}\partial_{\theta_k}\hat{\rho}_{\boldsymbol{\theta s}}\right)}{\delta\theta_r^2\mathrm{Tr}\left(\partial_{\theta_m}\partial_{\theta_r}\hat{\rho}_{\boldsymbol{\theta s}}\partial_{\theta_r}\hat{\rho}_{\boldsymbol{\theta s}}\right)}&=\sum_{m=1}^{q}\frac{\mathrm{Tr}\left(\partial_{\theta_j}\hat{\rho}_{\boldsymbol{\theta s}}\partial_{\theta_m}\partial_{\theta_k}\hat{\rho}_{\boldsymbol{\theta s}}\right)\mathrm{Tr}\left(\partial_{\theta_m}\partial_{\theta_k}\hat{\rho}_{\boldsymbol{\theta s}}\partial_{\theta_k}\hat{\rho}_{\boldsymbol{\theta s}}\right)}{\mathrm{Tr}\left(\partial_{\theta_m}\partial_{\theta_k}\hat{\rho}_{\boldsymbol{\theta s}}\partial_{\theta_k}\hat{\rho}_{\boldsymbol{\theta s}}\right)}\\&=\sum_{m=1}^{q}\mathrm{Tr}\left(\partial_{\theta_j}\hat{\rho}_{\boldsymbol{\theta s}}\partial_{\theta_k}\partial_{\theta_m}\hat{\rho}_{\boldsymbol{\theta s}}\right),\end{aligned}\tag{6.6}$$

since the limiting expression for the elements of the FIM at the point θ_s should be independent of the direction in which the state is expanded to calculate the above [16], such that we can choose $r=j$ or $r=k$ for our convenience.

Also, we get

$$\frac{\mathrm{Tr}\left(\partial_{\theta_j}\hat{\rho}_{\boldsymbol{\theta s}}\hat{P}_{q+1}\right)\mathrm{Tr}\left(\hat{P}_{q+1}\partial_{\theta_k}\hat{\rho}_{\boldsymbol{\theta s}}\right)}{\mathrm{Tr}\left[\hat{P}_{q+1}\left(\hat{\rho}_{\boldsymbol{\theta s}}+\delta\theta_r\partial_{\theta_r}\hat{\rho}_{\boldsymbol{\theta s}}\right)\right]}=\frac{\mathrm{Tr}\left(\partial_{\theta_j}\hat{\rho}_{\boldsymbol{\theta s}}\hat{P}_{q+1}\right)\mathrm{Tr}\left(\hat{P}_{q+1}\partial_{\theta_k}\hat{\rho}_{\boldsymbol{\theta s}}\right)}{\mathrm{Tr}\left(\hat{\rho}_{\boldsymbol{\theta s}}\hat{P}_{q+1}\right)+\delta\theta_r\mathrm{Tr}\left(\partial_{\theta_r}\hat{\rho}_{\boldsymbol{\theta s}}\hat{P}_{q+1}\right)}=0,\tag{6.7}$$

since $\mathrm{Tr}\left(\partial_{\theta_j}\hat{\rho}_{\boldsymbol{\theta s}}\hat{P}_{q+1}\right)=0$ for the normalising element $\hat{P}_{q+1}$.

Thus, (5.7) here becomes:

$$J_C^{jk}=\sum_{m=1}^{q}\mathrm{Tr}\left(\partial_{\theta_j}\hat{\rho}_{\boldsymbol{\theta s}}\partial_{\theta_k}\hat{P}_m\right)=-\mathrm{Tr}\left(\partial_{\theta_j}\partial_{\theta_k}\hat{\rho}_{\boldsymbol{\theta s}}\hat{P}_m\right)=-\sum_{m=1}^{q}\mathrm{Tr}\left(\partial_{\theta_j}\partial_{\theta_k}\hat{\rho}_{\boldsymbol{\theta s}}\partial_{\theta_m}\hat{\rho}_{\boldsymbol{\theta s}}\right),\tag{6.8}$$

where the second equality arises from $\mathrm{Tr}\left(\partial_{\theta_j}\hat{\rho}_{\boldsymbol{\theta s}}\hat{P}_m\right)=0$ upon differentiating both sides with respect to θ_k.

Furthermore, (5.8) here becomes:

$$\sum_{m=1}^{q}\partial_{\theta_m}\hat{\rho}_{\boldsymbol{\theta s}}=\mathbb{1}-\hat{\rho}_{\boldsymbol{\theta s}}-\hat{P}_{q+1}.\tag{6.9}$$

Then, (5.9) here becomes

$$\begin{aligned}J_C^{jk}&=-\mathrm{Tr}\left(\partial_{\theta_j}\partial_{\theta_k}\hat{\rho}_{\boldsymbol{\theta s}}\right)-\mathrm{Tr}\left(\partial_{\theta_j}\hat{\rho}_{\boldsymbol{\theta s}}\partial_{\theta_k}\hat{\rho}_{\boldsymbol{\theta s}}\right)+\mathrm{Tr}\left(\partial_{\theta_j}\partial_{\theta_k}\hat{\rho}_{\boldsymbol{\theta s}}\hat{P}_{q+1}\right)\\&=-\mathrm{Tr}\left(\partial_{\theta_j}\partial_{\theta_k}\hat{\rho}_{\boldsymbol{\theta s}}\right)-\mathrm{Tr}\left(\partial_{\theta_j}\hat{\rho}_{\boldsymbol{\theta s}}\right)\mathrm{Tr}\left(\partial_{\theta_k}\hat{\rho}_{\boldsymbol{\theta s}}\right)\\&=-4\mathrm{Re}\left[\mathrm{Tr}\left(\partial_{\theta_k}\hat{U}^\dagger_{\boldsymbol{\theta}}\partial_{\theta_j}\hat{U}_{\boldsymbol{\theta}}\hat{\rho}\right)+\mathrm{Tr}\left(\partial_{\theta_j}\hat{U}^\dagger_{\boldsymbol{\theta}}\hat{U}_{\boldsymbol{\theta}}\hat{\rho}\right)\mathrm{Tr}\left(\hat{U}^\dagger_{\boldsymbol{\theta}}\partial_{\theta_k}\hat{U}_{\boldsymbol{\theta}}\hat{\rho}\right)\right]\\&=4\mathrm{Re}\left[\mathrm{Tr}\left(\partial_{\theta_j}\hat{U}^\dagger_{\boldsymbol{\theta}}\partial_{\theta_k}\hat{U}_{\boldsymbol{\theta}}\hat{\rho}\right)+\mathrm{Tr}\left(\partial_{\theta_j}\hat{U}^\dagger_{\boldsymbol{\theta}}\hat{U}_{\boldsymbol{\theta}}\hat{\rho}\right)\mathrm{Tr}\left(\partial_{\theta_k}\hat{U}^\dagger_{\boldsymbol{\theta}}\hat{U}_{\boldsymbol{\theta}}\hat{\rho}\right)\right]\\&=4\mathrm{Re}\left[\mathrm{Tr}\left(\hat{U}_{\boldsymbol{\theta}}\partial_{\theta_j}\hat{U}^\dagger_{\boldsymbol{\theta}}\partial_{\theta_k}\hat{U}_{\boldsymbol{\theta}}\hat{U}^\dagger_{\boldsymbol{\theta}}\hat{\rho}_{\boldsymbol{\theta}}\right)+\mathrm{Tr}\left(\hat{U}_{\boldsymbol{\theta}}\partial_{\theta_j}\hat{U}^\dagger_{\boldsymbol{\theta}}\hat{\rho}_{\boldsymbol{\theta}}\right)\mathrm{Tr}\left(\hat{U}_{\boldsymbol{\theta}}\partial_{\theta_k}\hat{U}^\dagger_{\boldsymbol{\theta}}\hat{\rho}_{\boldsymbol{\theta}}\right)\right]=C_Q^{jk}.\end{aligned}\tag{6.10}$$

Here, we used the fact that $\partial_{\theta_k}\hat{U}^\dagger_{\boldsymbol{\theta}}\hat{U}_{\boldsymbol{\theta}}=-\hat{U}^\dagger_{\boldsymbol{\theta}}\partial_{\theta_k}\hat{U}_{\boldsymbol{\theta}}$, arising from $\hat{U}^\dagger_{\boldsymbol{\theta}}\hat{U}_{\boldsymbol{\theta}}=\mathbb{1}$ upon differentiating both sides with respect to θ_k, and that $\mathrm{Tr}\left(\partial_{\theta_k}\hat{U}_{\boldsymbol{\theta}}\hat{\rho}\hat{U}^\dagger_{\boldsymbol{\theta}}\right)=-\mathrm{Tr}\left(\hat{U}_{\boldsymbol{\theta}}\hat{\rho}\partial_{\theta_k}\hat{U}^\dagger_{\boldsymbol{\theta}}\right)$, arising from $\mathrm{Tr}\left(\hat{\rho}_{\boldsymbol{\theta s}}\right)=\mathrm{Tr}\left(\hat{U}_{\boldsymbol{\theta}}\hat{\rho}\hat{U}^\dagger_{\boldsymbol{\theta}}\right)=1$ upon differentiating both sides with respect to θ_k, and that $2\mathrm{Re}\left[\partial_{\theta_j}\hat{U}^\dagger_{\boldsymbol{\theta}}\partial_{\theta_k}\hat{U}_{\boldsymbol{\theta}}\right]=-2\mathrm{Re}\left[\partial_{\theta_k}\hat{U}^\dagger_{\boldsymbol{\theta}}\partial_{\theta_j}\hat{U}_{\boldsymbol{\theta}}\right]$, arising from $\hat{U}^\dagger_{\boldsymbol{\theta}}\hat{U}_{\boldsymbol{\theta}}=\mathbb{1}$ upon differentiating both sides with respect to θ_k and then θ_j. Also, $\mathrm{Tr}\left(\partial_{\theta_j}\partial_{\theta_k}\hat{\rho}_{\boldsymbol{\theta s}}\hat{P}_{q+1}\right)=0$, since $\mathrm{Tr}\left(\partial_{\theta_j}\hat{\rho}_{\boldsymbol{\theta s}}\hat{P}_{q+1}\right)=0$.

Note that $\mathrm{Tr}\left(\hat{\rho}_{\boldsymbol{\theta s}}\hat{P}_m\right)=0$, $\forall m=1,\dots,q$, but $\mathrm{Tr}\left(\hat{\rho}_{\boldsymbol{\theta s}}\hat{P}_{q+1}\right)\geq 0$, such that

$$\sum_{m=1}^{q}\mathrm{Tr}\left[\hat{\rho}_{\boldsymbol{\theta s}}\hat{P}_m\right]=0\Rightarrow\mathrm{Tr}\left[\hat{\rho}_{\boldsymbol{\theta s}}\left(\mathbb{1}-\hat{\rho}_{\boldsymbol{\theta s}}-\hat{P}_{q+1}\right)\right]=0\Rightarrow\mathrm{Tr}\left(\hat{\rho}^2_{\boldsymbol{\theta s}}\right)=1-\mathrm{Tr}\left(\hat{\rho}_{\boldsymbol{\theta s}}\hat{P}_{q+1}\right)\leq 1,\tag{6.11}$$

where the equality holds, when $\mathrm{Tr}\left(\hat{\rho}_{\boldsymbol{\theta}s}\hat{P}_{q+1}\right)=0$, and consequently, $\hat{\rho}_{\boldsymbol{\theta}s}$ is pure. However, we have from above that $\mathrm{Tr}\left(\hat{\rho}_{\boldsymbol{\theta}s}\hat{P}_{q+1}\right)=p\left(q+1|\theta_s\right)=1-\mathrm{Tr}\left(\hat{\rho}^2_{\boldsymbol{\theta}s}\right)$, which upon differentiation with respect to θ_j yields $\mathrm{Tr}\left(\partial_{\theta_j}\hat{\rho}_{\boldsymbol{\theta}s}\hat{P}_{q+1}\right)+\mathrm{Tr}\left[\hat{\rho}_{\boldsymbol{\theta}s}\partial_{\theta_j}\hat{P}_{q+1}\right]=-2\mathrm{Tr}\left(\partial_{\theta_j}\hat{\rho}_{\boldsymbol{\theta}s}\hat{\rho}_{\boldsymbol{\theta}s}\right)=0$. Clearly, from (6.9), upon differentiating both sides with respect to θ_j, multiplying both sides by $\hat{\rho}_{\boldsymbol{\theta}s}$, which is positive definite, and then taking trace of both sides, we get $\mathrm{Tr}\left[\partial_{\theta_j}\hat{P}_{q+1}\hat{\rho}_{\boldsymbol{\theta}s}\right]=-\mathrm{Tr}\left(\partial_{\theta_j}\hat{\rho}_{\boldsymbol{\theta}s}\hat{\rho}_{\boldsymbol{\theta}s}\right)-\sum_{m=1}^{q}\mathrm{Tr}\left(\partial_{\theta_j}\partial_{\theta_m}\hat{\rho}_{\boldsymbol{\theta}s}\hat{\rho}_{\boldsymbol{\theta}s}\right)=0$. Thus, we indeed have $\mathrm{Tr}\left(\partial_{\theta_j}\hat{\rho}_{\boldsymbol{\theta}s}\hat{P}_{q+1}\right)=0$, as used earlier.

VII. CONDITION TO SATURATE UPPER BOUND TO QFIM

Here, we prove that, as claimed in the main text, the following is a necessary and sufficient condition

$$\mathrm{Im}\left[\sum_l \mathrm{Tr}\left\{\left(\frac{\partial\hat{\Pi}_l^\dagger(\boldsymbol{\theta})}{\partial\theta_j}\frac{\partial\hat{\Pi}_l(\boldsymbol{\theta})}{\partial\theta_k}\right)\hat{\rho}\right\}\right]=0 \qquad \forall j,k \tag{7.1}$$

for the following upper bound to the ALD-based QFIM to be saturated:

$$C_Q^{jk}=4\mathrm{Re}\left[\mathrm{Tr}\left(\sum_l\frac{\partial\hat{\Pi}_l^\dagger(\boldsymbol{\theta})}{\partial\theta_j}\frac{\partial\hat{\Pi}_l(\boldsymbol{\theta})}{\partial\theta_k}\hat{\rho}\right)+\mathrm{Tr}\left(\sum_p\frac{\partial\hat{\Pi}_p^\dagger(\boldsymbol{\theta})}{\partial\theta_j}\hat{\Pi}_p(\boldsymbol{\theta})\hat{\rho}\right)\mathrm{Tr}\left(\sum_r\frac{\partial\hat{\Pi}_r^\dagger(\boldsymbol{\theta})}{\partial\theta_k}\hat{\Pi}_r(\boldsymbol{\theta})\hat{\rho}\right)\right]. \tag{7.2}$$

Consider that our initial probe state is pure, i.e. $\hat{\rho}=|\psi\rangle\langle\psi|$. Then, the unitary evolution $\hat{U}_{SB}(\boldsymbol{\theta})$ in the $S+B$ space can be considered equivalent to the output impure state $\sum_l\hat{\Pi}_l(\boldsymbol{\theta})|\psi\rangle\langle\psi|\hat{\Pi}_l^\dagger(\boldsymbol{\theta})$ of the noisy channel in the system S space, subsequently purified by extending the S space, introducing ancillas B. For the sake of clarity, we use the notation $\hat{U}_{\boldsymbol{\theta}}^{(S+B)}:=\hat{U}_{S+B}(\boldsymbol{\theta})$ here, in distinction from $\hat{U}_{SB}(\boldsymbol{\theta})$. The overall output is then a pure state denoted as $\hat{\rho}_{\boldsymbol{\theta}}^{S+B}=|\psi_{\boldsymbol{\theta}}^{S+B}\rangle\langle\psi_{\boldsymbol{\theta}}^{S+B}|$. Then, the QCRB (1.1) in the $S+B$ space can be saturated, when (2.1) leading here to

$$\mathrm{Im}\left[\mathrm{Tr}\left\{\left(\partial_{\theta_j}\hat{U}_{\boldsymbol{\theta}}^{(S+B)\dagger}\partial_{\theta_k}\hat{U}_{\boldsymbol{\theta}}^{(S+B)}\right)\left(|\psi\rangle\langle\psi|\otimes|0_B\rangle\langle 0_B|\right)\right\}\right]=0 \tag{7.3}$$

is satisfied, where $|0_B\rangle$ is a vacuum state ancillary bath. Tracing out B in (7.3), we get (7.1) as a necessary condition for the set of POVMs $\{\hat{P}_{n2}\}$ to result in (8.6) (See Section VIII), since the operators $\frac{\partial\hat{\Pi}_l(\boldsymbol{\theta})}{\partial\theta_k}$ do not act on B.

Now, consider that the initial probe state $\hat{\rho}$ is not pure. It can be purified by extending the system S space, introducing ancillas S'. Then, (7.1) can be applied to the pure state $|\psi^{S+S'}\rangle$ in the initial enlarged $S+S'$ space. Since the operators $\frac{\partial\hat{\Pi}_l(\boldsymbol{\theta})}{\partial\theta_k}$ do not act on S', we get (7.1) again as a necessary condition for the set of POVMs $\{\hat{P}_{n3}\}$ to result in (9.3) (See Section IX).

Next, we assume that the condition (7.1) saturates the upper bound (7.2) to the QFIM. We consider that the initial probe state is pure. Then, the output impure state of the noisy channel in the S space can be purified by extending the final system S space by introducing ancillas B. Since both the input and output states are pure, the channel in the $S+B$ space is unitary $\hat{U}_{\boldsymbol{\theta}}^{(S+B)}$. Then, since the operators $\frac{\partial\hat{\Pi}_l(\boldsymbol{\theta})}{\partial\theta_k}$ do not act on B, (7.1) saturating (7.2) in the S space implies that (7.3) saturates the QCRB (1.1) in the $S+B$ space. Thus, (7.1) is a sufficient condition for the set of POVMs $\{\hat{P}_{n2}\}$ to result in (8.6).

Now, considering that the initial probe state is not pure, it can be purified by extending the initial system S space by introducing ancillas S'. Then, since the operators $\frac{\partial\hat{\Pi}_l(\boldsymbol{\theta})}{\partial\theta_k}$ do not act on S', (7.1) saturating (7.2) in the S space implies that (7.3) saturates the QCRB (1.1) in the $S+B+S'$ space, with $\hat{U}_{\boldsymbol{\theta}}^{(S+B)}$ replaced by $\hat{U}_{\boldsymbol{\theta}}^{(S+B+S')}$ and $|\psi\rangle$ replaced by $|\psi^{S+S'}\rangle$. Thus, we again get (7.1) as a sufficient condition for the set of POVMs $\{\hat{P}_{n3}\}$ to result in (9.3).

VIII. POVM TO ATTAIN QFIM UPPER BOUND FOR PURE STATE INPUT VIA NOISY CHANNEL

Here, we prove that, as claimed in the main text, the set of POVMs $\{\hat{P}_{n2}\}$ of cardinality $q+2$, comprising the following $q+1$ elements,

$$\hat{P}_0=\hat{\rho}(\boldsymbol{\theta})=\sum_l\hat{\Pi}_l(\boldsymbol{\theta})|\psi\rangle\langle\psi|\hat{\Pi}_l^\dagger(\boldsymbol{\theta}),\quad \hat{P}_m=\frac{\partial\hat{\rho}(\boldsymbol{\theta})}{\partial\theta_m}=\sum_l\left[\frac{\partial\hat{\Pi}_l(\boldsymbol{\theta})}{\partial\theta_m}|\psi\rangle\langle\psi|\hat{\Pi}_l^\dagger(\boldsymbol{\theta})+\hat{\Pi}_l(\boldsymbol{\theta})|\psi\rangle\langle\psi|\frac{\partial\hat{\Pi}_l^\dagger(\boldsymbol{\theta})}{\partial\theta_m}\right]\quad\forall m=1,\ldots,q, \tag{8.1}$$

together with one element accounting for normalisation, saturates (7.2), provided (7.1) is satisfied.

We again consider initial pure state $\hat{\rho} = |\psi\rangle\langle\psi|$, and the unitary evolution in the $S+B$ space, $\hat{U}_{\boldsymbol{\theta}}^{(S+B)}$ here, in distinction from $\hat{U}_{SB}(\boldsymbol{\theta})$ used in the main text.

Then, the elements of the QFIM, as in (6.2), in terms of the initial pure state $|\psi\rangle$ here are

$$\begin{aligned} J_Q^{jk,S+B} &= 4\mathrm{Re}\Big[\mathrm{Tr}\left(\partial_{\theta_j}\hat{U}_{\boldsymbol{\theta}}^{(S+B)\dagger}\partial_{\theta_k}\hat{U}_{\boldsymbol{\theta}}^{(S+B)}\left(|\psi\rangle\langle\psi|\otimes|0_B\rangle\langle 0_B|\right)\right) \\ &\quad+\mathrm{Tr}\left(\partial_{\theta_j}\hat{U}_{\boldsymbol{\theta}}^{(S+B)\dagger}\hat{U}_{\boldsymbol{\theta}}^{(S+B)}\left(|\psi\rangle\langle\psi|\otimes|0_B\rangle\langle 0_B|\right)\right)\mathrm{Tr}\left(\partial_{\theta_k}\hat{U}_{\boldsymbol{\theta}}^{(S+B)\dagger}\hat{U}_{\boldsymbol{\theta}}^{(S+B)}\left(|\psi\rangle\langle\psi|\otimes|0_B\rangle\langle 0_B|\right)\right)\Big], \end{aligned} \tag{8.2}$$

where $|0_B\rangle$ is a vacuum state ancillary bath.

Tracing out B from above, we get the upper bound (7.2) to the QFIM in terms of the initial pure state $|\psi\rangle$ in the S space:

$$C_Q^{jk} = 4\mathrm{Re}\left[\mathrm{Tr}\left(\sum_l \partial_{\theta_j}\hat{\Pi}_{\boldsymbol{\theta}l}^{\dagger}\partial_{\theta_k}\hat{\Pi}_{\boldsymbol{\theta}l}|\psi\rangle\langle\psi|\right)+\mathrm{Tr}\left(\sum_p \partial_{\theta_j}\hat{\Pi}_{\boldsymbol{\theta}p}^{\dagger}\hat{\Pi}_{\boldsymbol{\theta}p}|\psi\rangle\langle\psi|\right)\mathrm{Tr}\left(\sum_r \partial_{\theta_k}\hat{\Pi}_{\boldsymbol{\theta}r}^{\dagger}\hat{\Pi}_{\boldsymbol{\theta}r}|\psi\rangle\langle\psi|\right)\right], \tag{8.3}$$

where we used the short notations $\hat{\Pi}_{\boldsymbol{\theta}l} = \hat{\Pi}_l(\boldsymbol{\theta})$ and $\partial_{\theta_k}\hat{\Pi}_{\boldsymbol{\theta}l} = \frac{\partial\hat{\Pi}_l(\boldsymbol{\theta})}{\partial\theta_k}$.

Now, since the operators, $\hat{\Pi}_{\boldsymbol{\theta}l}$ and $\partial_{\theta_k}\hat{\Pi}_{\boldsymbol{\theta}l}$, do not act on B, when (7.1) is satisfied, the bound C_Q is the actual QFIM J_Q in the S space, with the set of POVMs $\{\hat{P}_{n2}\}$ saturating the corresponding QCRB for unital channel (see Section X).

Also, the elements of the FIM J_C, as defined in (1.3), are again as in (6.4). Consider again that we are interested in saturating the bound at a specific point θ_s in the space of $\boldsymbol{\theta}$, as in Ref. [16]. Then, (6.5) and (6.6) remain the same. But, (6.7) becomes

$$\frac{\mathrm{Tr}\left(\partial_{\theta_j}\hat{\rho}_{\boldsymbol{\theta}s}|\Phi_n\rangle\langle\Phi_n|\right)\mathrm{Tr}\left(|\Phi_n\rangle\langle\Phi_n|\partial_{\theta_k}\hat{\rho}_{\boldsymbol{\theta}s}\right)}{\mathrm{Tr}\left[|\Phi_n\rangle\langle\Phi_n|\left(\hat{\rho}_{\boldsymbol{\theta}s}+\delta\theta_r\partial_{\theta_r}\hat{\rho}_{\boldsymbol{\theta}s}\right)\right]} = \frac{\langle\Phi_n|\partial_{\theta_j}\hat{\rho}_{\boldsymbol{\theta}s}|\Phi_n\rangle\langle\Phi_n|\partial_{\theta_k}\hat{\rho}_{\boldsymbol{\theta}s}|\Phi_n\rangle}{\langle\Phi_n|\hat{\rho}_{\boldsymbol{\theta}s}|\Phi_n\rangle+\delta\theta_r\langle\Phi_n|\partial_{\theta_r}\hat{\rho}_{\boldsymbol{\theta}s}|\Phi_n\rangle} = 0, \tag{8.4}$$

since $\langle\Phi_n|\partial_{\theta_j}\hat{\rho}_{\boldsymbol{\theta}s}|\Phi_n\rangle = 0$. Here, we used the normalising element $|\Phi_n\rangle\langle\Phi_n|$, in distinction from $|\phi_n\rangle\langle\phi_n|$ used in (5.6). Then, (6.8) remains the same. But, (6.9) here becomes:

$$\sum_{m=1}^{q}\partial_{\theta_m}\hat{\rho}_{\boldsymbol{\theta}s} = \mathbb{1} - \hat{\rho}_{\boldsymbol{\theta}s} - |\Phi_n\rangle\langle\Phi_n|. \tag{8.5}$$

Then, (6.10) here becomes

$$\begin{aligned} J_C^{jk} &= -\mathrm{Tr}\left(\partial_{\theta_j}\partial_{\theta_k}\hat{\rho}_{\boldsymbol{\theta}s}\right) - \mathrm{Tr}\left(\partial_{\theta_j}\hat{\rho}_{\boldsymbol{\theta}s}\partial_{\theta_k}\hat{\rho}_{\boldsymbol{\theta}s}\right) + \mathrm{Tr}\left(\partial_{\theta_j}\partial_{\theta_k}\hat{\rho}_{\boldsymbol{\theta}s}|\Phi_n\rangle\langle\Phi_n|\right) \\ &= -\mathrm{Tr}\left(\partial_{\theta_j}\partial_{\theta_k}\hat{\rho}_{\boldsymbol{\theta}s}\right) - \mathrm{Tr}\left(\partial_{\theta_j}\hat{\rho}_{\boldsymbol{\theta}s}\right)\mathrm{Tr}\left(\partial_{\theta_k}\hat{\rho}_{\boldsymbol{\theta}s}\right) \\ &= -4\mathrm{Re}\left[\mathrm{Tr}\left(\sum_l \partial_{\theta_k}\hat{\Pi}_{\boldsymbol{\theta}l}^{\dagger}\partial_{\theta_j}\hat{\Pi}_{\boldsymbol{\theta}l}|\psi\rangle\langle\psi|\right)\right] - 4\mathrm{Tr}\left(\sum_p \partial_{\theta_j}\hat{\Pi}_{\boldsymbol{\theta}p}^{\dagger}\hat{\Pi}_{\boldsymbol{\theta}p}|\psi\rangle\langle\psi|\right)\mathrm{Tr}\left(\sum_r \hat{\Pi}_{\boldsymbol{\theta}r}^{\dagger}\partial_{\theta_k}\hat{\Pi}_{\boldsymbol{\theta}r}|\psi\rangle\langle\psi|\right) \\ &= 4\mathrm{Re}\left[\mathrm{Tr}\left(\sum_l \partial_{\theta_j}\hat{\Pi}_{\boldsymbol{\theta}l}^{\dagger}\partial_{\theta_k}\hat{\Pi}_{\boldsymbol{\theta}l}|\psi\rangle\langle\psi|\right)+\mathrm{Tr}\left(\sum_p \partial_{\theta_j}\hat{\Pi}_{\boldsymbol{\theta}p}^{\dagger}\hat{\Pi}_{\boldsymbol{\theta}p}|\psi\rangle\langle\psi|\right)\mathrm{Tr}\left(\sum_r \partial_{\theta_k}\hat{\Pi}_{\boldsymbol{\theta}r}^{\dagger}\hat{\Pi}_{\boldsymbol{\theta}r}|\psi\rangle\langle\psi|\right)\right] = C_Q^{jk}, \end{aligned} \tag{8.6}$$

noting that $\langle\psi|\hat{O}|\psi\rangle$ is, by definition, real for some operator $\hat{O}$.

Here, we used the fact that $\sum_l \partial_{\theta_k}\hat{\Pi}_{\boldsymbol{\theta}l}^{\dagger}\hat{\Pi}_{\boldsymbol{\theta}l} = -\sum_l \hat{\Pi}_{\boldsymbol{\theta}l}^{\dagger}\partial_{\theta_k}\hat{\Pi}_{\boldsymbol{\theta}l}$, arising from $\sum_l \hat{\Pi}_{\boldsymbol{\theta}l}^{\dagger}\hat{\Pi}_{\boldsymbol{\theta}l} = \mathbb{1}$ upon differentiating both sides with respect to θ_k, and that $\sum_l \mathrm{Tr}\left(\partial_{\theta_k}\hat{\Pi}_{\boldsymbol{\theta}l}|\psi\rangle\langle\psi|\hat{\Pi}_{\boldsymbol{\theta}l}^{\dagger}\right) = -\sum_l \mathrm{Tr}\left(\hat{\Pi}_{\boldsymbol{\theta}l}|\psi\rangle\langle\psi|\partial_{\theta_k}\hat{\Pi}_{\boldsymbol{\theta}l}^{\dagger}\right)$, arising from $\mathrm{Tr}\left(\hat{\rho}_{\boldsymbol{\theta}s}\right) = \sum_l \mathrm{Tr}\left(\hat{\Pi}_{\boldsymbol{\theta}l}|\psi\rangle\langle\psi|\hat{\Pi}_{\boldsymbol{\theta}l}^{\dagger}\right) = 1$ upon differentiating both sides with respect to θ_k, and that $2\mathrm{Re}\left[\sum_l \partial_{\theta_j}\hat{\Pi}_{\boldsymbol{\theta}l}^{\dagger}\partial_{\theta_k}\hat{\Pi}_{\boldsymbol{\theta}l}\right] = -2\mathrm{Re}\left[\sum_l \partial_{\theta_k}\hat{\Pi}_{\boldsymbol{\theta}l}^{\dagger}\partial_{\theta_j}\hat{\Pi}_{\boldsymbol{\theta}l}\right]$, arising from $\sum_l \hat{\Pi}_{\boldsymbol{\theta}l}^{\dagger}\hat{\Pi}_{\boldsymbol{\theta}l} = \mathbb{1}$ upon differentiating both sides with respect to θ_k and then θ_j. Also, $\mathrm{Tr}\left(\partial_{\theta_j}\partial_{\theta_k}\hat{\rho}_{\boldsymbol{\theta}s}|\Phi_n\rangle\langle\Phi_n|\right) = \langle\Phi_n|\partial_{\theta_j}\partial_{\theta_k}\hat{\rho}_{\boldsymbol{\theta}s}|\Phi_n\rangle = 0$, since $\langle\Phi_n|\partial_{\theta_j}\hat{\rho}_{\boldsymbol{\theta}s}|\Phi_n\rangle = 0$.

IX. POVM TO ATTAIN QFIM UPPER BOUND FOR MIXED STATE INPUT VIA NOISY CHANNEL

Here, we prove that, as claimed in the main text, the set of POVMs $\{\hat{P}_{n3}\}$ of cardinality $q+2$, comprising the following $q+1$ elements,

$$\hat{P}_0 = \hat{\rho}(\boldsymbol{\theta}) = \sum_l \hat{\Pi}_l(\boldsymbol{\theta})\hat{\rho}\hat{\Pi}_l^\dagger(\boldsymbol{\theta}), \qquad \hat{P}_m = \frac{\partial\hat{\rho}(\boldsymbol{\theta})}{\partial\theta_m} = \sum_l \left[\frac{\partial\hat{\Pi}_l(\boldsymbol{\theta})}{\partial\theta_m}\hat{\rho}\hat{\Pi}_l^\dagger(\boldsymbol{\theta}) + \hat{\Pi}_l(\boldsymbol{\theta})\hat{\rho}\frac{\partial\hat{\Pi}_l^\dagger(\boldsymbol{\theta})}{\partial\theta_m}\right] \quad \forall m = 1,\ldots,q, \tag{9.1}$$

together with one element accounting for normalisation, saturates (7.2), provided (7.1) is satisfied.

Consider that the initial probe state $\hat{\rho}$ is impure. It can be purified by extending the system S space, introducing ancillas S'. Then, proceeding in a similar manner as in the previous section for the pure state $|\psi^{S+S'}\rangle$ in the initial enlarged $S+S'$ space, the upper bound (7.2) to the QFIM in terms of the initial state $\hat{\rho}$ in the S space is given by

$$C_Q^{jk} = 4\text{Re}\left[\text{Tr}\left(\sum_l \partial_{\theta_j}\hat{\Pi}_{\boldsymbol{\theta}l}^\dagger\partial_{\theta_k}\hat{\Pi}_{\boldsymbol{\theta}l}\hat{\rho}\right) + \text{Tr}\left(\sum_p \partial_{\theta_j}\hat{\Pi}_{\boldsymbol{\theta}p}^\dagger\hat{\Pi}_{\boldsymbol{\theta}p}\hat{\rho}\right)\text{Tr}\left(\sum_r \partial_{\theta_k}\hat{\Pi}_{\boldsymbol{\theta}r}^\dagger\hat{\Pi}_{\boldsymbol{\theta}r}\hat{\rho}\right)\right], \tag{9.2}$$

since the operators, $\hat{\Pi}_{\boldsymbol{\theta}l}$ and $\partial_{\theta_k}\hat{\Pi}_{\boldsymbol{\theta}l}$ do not act on S'. Moreover, when (7.1) is satisfied, the bound C_Q is the actual QFIM J_Q in the S space, with the set of POVMs $\{\hat{P}_{n3}\}$ saturating the corresponding QCRB for unital channel (see Section X).

Also, again the elements of the FIM J_C, as defined in (1.3), are as in (6.4). Consider again that we are interested in saturating the bound at a specific point θ_s in the space of $\boldsymbol{\theta}$, as in Ref. [16]. Then, (6.5), (6.6), (6.7), (6.8), (6.9) remain the same, with the normalising element again being $\hat{P}_{q+1}$. But, (6.10), which was (8.6) in the last section, becomes:

$$J_C^{jk} = 4\text{Re}\left[\text{Tr}\left(\sum_l \partial_{\theta_j}\hat{\Pi}_{\boldsymbol{\theta}l}^\dagger\partial_{\theta_k}\hat{\Pi}_{\boldsymbol{\theta}l}\hat{\rho}\right) + \text{Tr}\left(\sum_p \partial_{\theta_j}\hat{\Pi}_{\boldsymbol{\theta}p}^\dagger\hat{\Pi}_{\boldsymbol{\theta}p}\hat{\rho}\right)\text{Tr}\left(\sum_r \partial_{\theta_k}\hat{\Pi}_{\boldsymbol{\theta}r}^\dagger\hat{\Pi}_{\boldsymbol{\theta}r}\hat{\rho}\right)\right] = C_Q^{jk}, \tag{9.3}$$

which is as in (9.2).

X. NOISE IN CHANNEL CAN ALLOW TO BEAT THE HEISENBERG LIMIT

Here, we prove that noise in the quantum channel can allow to beat the Heisenberg precision limit, as claimed in the main text, when the following condition is satisfied by the Kraus operators $\hat{\Pi}_{\boldsymbol{\theta}l} = \hat{\Pi}_l(\boldsymbol{\theta})$ of the quantum channel:

$$\text{Im}\left[\sum_l \text{Tr}\left\{\left(\partial_{\theta_j}\hat{\Pi}_{\boldsymbol{\theta}l}^\dagger\partial_{\theta_k}\hat{\Pi}_{\boldsymbol{\theta}l}\right)\hat{\rho}\right\}\right] = 0, \qquad \forall\, j,k. \tag{10.1}$$

We have

$$\hat{\rho}_{\boldsymbol{\theta}} = \sum_l \hat{\Pi}_{\boldsymbol{\theta}l}\hat{\rho}\hat{\Pi}_{\boldsymbol{\theta}l}^\dagger \Rightarrow \partial_{\theta_k}\hat{\rho}_{\boldsymbol{\theta}} = \sum_l \left[\partial_{\theta_k}\hat{\Pi}_{\boldsymbol{\theta}l}\hat{\rho}\hat{\Pi}_{\boldsymbol{\theta}l}^\dagger + \hat{\Pi}_{\boldsymbol{\theta}l}\hat{\rho}\partial_{\theta_k}\hat{\Pi}_{\boldsymbol{\theta}l}^\dagger\right]. \tag{10.2}$$

Next, (10.1) saturates an ALD-based QCRB, corresponding to:

$$\partial_{\theta_k}\hat{\rho}_{\boldsymbol{\theta}} = \frac{1}{2}\left[\hat{O}_k\hat{\rho} + \hat{\rho}\hat{O}_k^\dagger\right] = \sum_l \left[\partial_{\theta_k}\hat{\Pi}_{\boldsymbol{\theta}l}\hat{\rho} + \hat{\rho}\partial_{\theta_k}\hat{\Pi}_{\boldsymbol{\theta}l}^\dagger\right], \tag{10.3}$$

where the ALDs are chosen to be:

$$\hat{O}_k = 2\sum_l \partial_{\theta_k}\hat{\Pi}_{\boldsymbol{\theta}l}. \tag{10.4}$$

Note that the choice of ALD need not be unique. Our purpose here is that it is enough to find one instance where the Heisenberg limit can be beaten. Also, strictly speaking, the above is not a valid ALD, since it is not a function of the probe state, hence our choice of the ALDs in the main text. Moreover, (10.3) is expressed in terms of the initial probe state and not the evolved probe state. However, for the purposes of our proof here, it suffices to consider the above for simplicity without loss of generality.

We start with assuming that when (10.1) is satisfied, the upper bound (7.2) to the QFIM equals the actual QFIM. In other words, the corresponding lower bound to the Heisenberg limit equals the Heisenberg limit, when (10.1) is satisfied, which is possible, when we have:

$$\sum_l \left[\partial_{\theta_k}\hat{\Pi}_{\boldsymbol{\theta}l}\hat{\rho}\hat{\Pi}^\dagger_{\boldsymbol{\theta}l} + \hat{\Pi}_{\boldsymbol{\theta}l}\hat{\rho}\partial_{\theta_k}\hat{\Pi}^\dagger_{\boldsymbol{\theta}l}\right] = \sum_l \left[\partial_{\theta_k}\hat{\Pi}_{\boldsymbol{\theta}l}\hat{\rho} + \hat{\rho}\partial_{\theta_k}\hat{\Pi}^\dagger_{\boldsymbol{\theta}l}\right], \tag{10.5}$$

following from (10.2) and (10.3). Now, the above is possible only when the following condition is satisfied:

$$\begin{aligned}
\sum_l \partial_{\theta_k}\hat{\Pi}_{\boldsymbol{\theta}l}\hat{\rho}\hat{\Pi}^\dagger_{\boldsymbol{\theta}l} &= \sum_l \partial_{\theta_k}\hat{\Pi}_{\boldsymbol{\theta}l}\hat{\rho} \\
\Rightarrow \mathrm{Tr}\left(\sum_l \partial_{\theta_k}\hat{\Pi}_{\boldsymbol{\theta}l}\hat{\rho}\hat{\Pi}^\dagger_{\boldsymbol{\theta}l}\right) &= \mathrm{Tr}\left(\sum_l \partial_{\theta_k}\hat{\Pi}_{\boldsymbol{\theta}l}\hat{\rho}\right) \\
\Rightarrow \mathrm{Tr}\left(\sum_l \hat{\Pi}^\dagger_{\boldsymbol{\theta}l}\partial_{\theta_k}\hat{\Pi}_{\boldsymbol{\theta}l}\hat{\rho}\right) &= \mathrm{Tr}\left(\sum_l \partial_{\theta_k}\hat{\Pi}_{\boldsymbol{\theta}l}\hat{\rho}\right) \\
\Rightarrow \mathrm{Tr}\left(\sum_l \partial_{\theta_k}\hat{\Pi}^\dagger_{\boldsymbol{\theta}l}\hat{\Pi}_{\boldsymbol{\theta}l}\hat{\rho}\right) &= \mathrm{Tr}\left(\sum_l \partial_{\theta_k}\hat{\Pi}^\dagger_{\boldsymbol{\theta}l}\hat{\rho}\right) \\
\Rightarrow \sum_l \mathrm{Tr}\left[\left(\partial_{\theta_j}\hat{\Pi}^\dagger_{\boldsymbol{\theta}l}\hat{\Pi}_{\boldsymbol{\theta}l}\hat{\Pi}^\dagger_{\boldsymbol{\theta}l}\partial_{\theta_k}\hat{\Pi}_{\boldsymbol{\theta}l}\right)\hat{\rho}\right] &= \sum_l \mathrm{Tr}\left[\left(\partial_{\theta_j}\hat{\Pi}^\dagger_{\boldsymbol{\theta}l}\partial_{\theta_k}\hat{\Pi}_{\boldsymbol{\theta}l}\right)\hat{\rho}\right],
\end{aligned} \tag{10.6}$$

which is possible when we have $\sum_l \hat{\Pi}_{\boldsymbol{\theta}l}\hat{\Pi}^\dagger_{\boldsymbol{\theta}l} = \mathbb{1}$, i.e. when the channel is unital. Thus, for unital channels the upper bound (7.2) to the QFIM equals the actual QFIM. Hence, if the channel is non-unital, then the upper bound (7.2) to the QFIM can be strictly larger than the actual QFIM, so that the Heisenberg limit may be beaten. Note that (10.6) modifies (10.1) to:

$$\mathrm{Im}\left[\sum_l \mathrm{Tr}\left\{\left(\partial_{\theta_j}\hat{\Pi}^\dagger_{\boldsymbol{\theta}l}\hat{\Pi}_{\boldsymbol{\theta}l}\hat{\Pi}^\dagger_{\boldsymbol{\theta}l}\partial_{\theta_k}\hat{\Pi}_{\boldsymbol{\theta}l}\right)\hat{\rho}\right\}\right] = 0, \qquad \forall\, j,k. \tag{10.7}$$

The fact that the channel indeed needs to be unital for the last line in (10.6) to hold may not be evident without an extra summation index. Let us, therefore, reconfirm this.

First, note that (10.7) saturates an ALD-based QCRB, corresponding to:

$$\partial_{\theta_k}\hat{\rho}_{\boldsymbol{\theta}} = \frac{1}{2}\left[\hat{L}_k\hat{\rho} + \hat{\rho}\hat{L}^\dagger_k\right] = \sum_l \left[\hat{\Pi}^\dagger_{\boldsymbol{\theta}l}\partial_{\theta_k}\hat{\Pi}_{\boldsymbol{\theta}l}\hat{\rho} + \hat{\rho}\partial_{\theta_k}\hat{\Pi}^\dagger_{\boldsymbol{\theta}l}\hat{\Pi}_{\boldsymbol{\theta}l}\right], \tag{10.8}$$

where the ALDs are chosen to be:

$$\hat{L}_k = 2\sum_l \hat{\Pi}^\dagger_{\boldsymbol{\theta}l}\partial_{\theta_k}\hat{\Pi}_{\boldsymbol{\theta}l}. \tag{10.9}$$

Now, in terms of the evolved probe state $\hat{\rho}_{\boldsymbol{\theta}} = \hat{\rho}(\boldsymbol{\theta})$, the condition (10.1) becomes:

$$\mathrm{Im}\left[\sum_l \mathrm{Tr}\left\{\left(\hat{\Pi}_{\boldsymbol{\theta}l}\partial_{\theta_j}\hat{\Pi}^\dagger_{\boldsymbol{\theta}l}\partial_{\theta_k}\hat{\Pi}_{\boldsymbol{\theta}l}\hat{\Pi}^\dagger_{\boldsymbol{\theta}l}\right)\hat{\rho}_{\boldsymbol{\theta}}\right\}\right] = 0, \qquad \forall\, j,k. \tag{10.10}$$

This is obtained from the saturability condition corresponding to $\hat{\rho}^{(S+B)}_{\boldsymbol{\theta}}$ in the $S+B$ space, by tracing out the bath B. And this is equivalent to the saturability condition (10.1).

Next, (10.10) saturates an ALD-based QCRB, corresponding to:

$$\partial_{\theta_k}\hat{\rho}_{\boldsymbol{\theta}} = \frac{1}{2}\left[\hat{Q}_k\hat{\rho}_{\boldsymbol{\theta}} + \hat{\rho}_{\boldsymbol{\theta}}\hat{Q}^\dagger_k\right] = \sum_l \left[\partial_{\theta_k}\hat{\Pi}_{\boldsymbol{\theta}l}\hat{\Pi}^\dagger_{\boldsymbol{\theta}l}\hat{\rho}_{\boldsymbol{\theta}} + \hat{\rho}_{\boldsymbol{\theta}}\hat{\Pi}_{\boldsymbol{\theta}l}\partial_{\theta_k}\hat{\Pi}^\dagger_{\boldsymbol{\theta}l}\right], \tag{10.11}$$

where the ALDs are chosen to be:

$$\hat{Q}_k = 2\sum_l \partial_{\theta_k}\hat{\Pi}_{\boldsymbol{\theta}l}\hat{\Pi}^\dagger_{\boldsymbol{\theta}l}. \tag{10.12}$$

Then, (10.5) holds, when the following holds:

$$\sum_l \left[\hat{\Pi}_{\boldsymbol{\theta}l}^\dagger \partial_{\theta_k} \hat{\Pi}_{\boldsymbol{\theta}l} \hat{\rho} + \hat{\rho} \partial_{\theta_k} \hat{\Pi}_{\boldsymbol{\theta}l}^\dagger \hat{\Pi}_{\boldsymbol{\theta}l}\right] = \sum_l \left[\partial_{\theta_k} \hat{\Pi}_{\boldsymbol{\theta}l} \hat{\Pi}_{\boldsymbol{\theta}l}^\dagger \hat{\rho}_{\boldsymbol{\theta}} + \hat{\rho}_{\boldsymbol{\theta}} \hat{\Pi}_{\boldsymbol{\theta}l} \partial_{\theta_k} \hat{\Pi}_{\boldsymbol{\theta}l}^\dagger\right], \tag{10.13}$$

that follows from (10.8) and (10.11).

Now, let us consider both $\hat{\rho}$ and $\hat{\rho}_{\boldsymbol{\theta}}$ to be maximally mixed. Then, (10.13) becomes:

$$\begin{aligned} \sum_l \left[\hat{\Pi}_{\boldsymbol{\theta}l}^\dagger \partial_{\theta_k} \hat{\Pi}_{\boldsymbol{\theta}l} + \partial_{\theta_k} \hat{\Pi}_{\boldsymbol{\theta}l}^\dagger \hat{\Pi}_{\boldsymbol{\theta}l}\right] &= \sum_l \left[\partial_{\theta_k} \hat{\Pi}_{\boldsymbol{\theta}l} \hat{\Pi}_{\boldsymbol{\theta}l}^\dagger + \hat{\Pi}_{\boldsymbol{\theta}l} \partial_{\theta_k} \hat{\Pi}_{\boldsymbol{\theta}l}^\dagger\right] \\ \Rightarrow \sum_l \partial_{\theta_k} \left[\hat{\Pi}_{\boldsymbol{\theta}l}^\dagger \hat{\Pi}_{\boldsymbol{\theta}l}\right] &= \sum_l \partial_{\theta_k} \left[\hat{\Pi}_{\boldsymbol{\theta}l} \hat{\Pi}_{\boldsymbol{\theta}l}^\dagger\right] \\ \Rightarrow \mathbb{1} &= \sum_l \hat{\Pi}_{\boldsymbol{\theta}l} \hat{\Pi}_{\boldsymbol{\theta}l}^\dagger \qquad \because \sum_l \hat{\Pi}_{\boldsymbol{\theta}l}^\dagger \hat{\Pi}_{\boldsymbol{\theta}l} = \mathbb{1}, \end{aligned} \tag{10.14}$$

where the last line follows from the previous line without an additional constant, since we must also have:

$$\hat{\rho}_{\boldsymbol{\theta}} = \sum_l \hat{\Pi}_{\boldsymbol{\theta}l} \hat{\rho} \hat{\Pi}_{\boldsymbol{\theta}l}^\dagger \Rightarrow \mathbb{1} = \sum_l \hat{\Pi}_{\boldsymbol{\theta}l} \hat{\Pi}_{\boldsymbol{\theta}l}^\dagger, \tag{10.15}$$

when both $\hat{\rho}$ and $\hat{\rho}_{\boldsymbol{\theta}}$ are maximally mixed. Indeed, both the initial and evolved probe states can be maximally mixed, only if the noisy channel is unital. Thus, the channel indeed needs to be unital for (10.13), and therefore, (10.6) to hold.

---

[1] M. Tsang, H. M. Wiseman, and C. M. Caves, Physical Review Letters **106**, 090401 (2011).
[2] S. Ragy, M. Jarzyna, and R. Demkowicz-Dobrzański, Physical Review A **94**, 052108 (2016).
[3] H. Nagaoka, in *Asymptotic Theory of Quantum Statistical Inference*, Vol. 1, edited by M. Hayashi (World Scientific, 1989) Chap. 8.
[4] S. L. Braunstein and C. M. Caves, Physical Review Letters **72**, 3439 (1994).
[5] M. A. Nielsen and I. L. Chuang, *Quantum Computation and Quantum Information* (Cambridge University Press, 2002).
[6] M. G. A. Paris, International Journal of Quantum Information **7**, 125 (2009).
[7] J.-D. Yue, Y.-R. Zhang, and H. Fan, Scientific Reports **4**, 5933 (2014).
[8] M. Hayashi, *Quantum Information - An Introduction* (Springer, 2006).
[9] J. Dittmann, Journal of Physics A: Mathematical and General **32**, 2663 (1999).
[10] A. D. Pasquale, D. Rossini, P. Facchi, and V. Giovannetti, Physical Review A **88**, 052117 (2013).
[11] O. Fawzi and R. Renner, Communications in Mathematical Physics **340**, 575 (2015).
[12] J. L. Dodd and M. A. Nielsen, Physical Review A **66**, 044301 (2002).
[13] R. Demkowicz-Dobrzański and L. Maccone, Physical Review Letters **113**, 250801 (2014).
[14] D. Petz and C. Ghinea, "Introduction to quantum Fisher information," in *Quantum Probability and Related Topics* (World Scientific, 2011) pp. 261–281.
[15] B. M. Escher, R. L. de Matos Filho, and L. Davidovich, Nature Physics **7**, 406 (2011).
[16] P. C. Humphreys, M. Barbieri, A. Datta, and I. A. Walmsley, Physical Review Letters **111**, 070403 (2013).
[17] T. Baumgratz and A. Datta, Physical Review Letters **116**, 030801 (2016).
[18] S. L. Braunstein, C. M. Caves, and G. J. Milburn, Annals of Physics **247**, 135 (1996).
